# Importin-8 silencing reduces Ataxin-3 aggregation and alleviates Machado-Joseph disease/spinocerebellar ataxia type 3

**DOI:** 10.1016/j.isci.2026.116446

**Published:** 2026-08-26

**Authors:** Inês Morgado Martins, Anna Sergeevna Sowa, David Rufino-Ramos, Diana Duarte Lobo, Kevin Leandro, Ana Vasconcelos-Ferreira, Dina Pereira, Olaf Rieβ, Thorsten Schmidt, Luís Pereira de Almeida

**Affiliations:** 1 CNC-Center for Neuroscience and Cell Biology, Coimbra, Portugal; 2 CIBB – Center for Innovative Biomedicine and Biotechnology, University of Coimbra, Coimbra, Portugal; 3 IIIUC – Institute for Interdisciplinary Research, University of Coimbra, Coimbra, Portugal; 4 Institute of Medical Genetics and Applied Genomics, Eberhard Karls Universität Tübingen, Tübingen, Germany; 5 Center for Rare Diseases (ZSE), University Hospital Tübingen, Tübingen, Germany; 6 Faculty of Pharmacy, University of Coimbra, Coimbra, Portugal; 7 GeneT - Gene Therapy Center of Excellence, Coimbra, Portugal

**Keywords:** Nucleocytoplasmic transport, Importin-8, Ataxin-3 aggregation, Machado-Joseph disease, NF-kB/p65 translocation, Argonaute-2

## Abstract

Machado-Joseph disease (MJD) is a polyglutamine neurodegenerative disorder characterized by the formation of neuronal intranuclear Ataxin-3 inclusions. The nucleocytoplasmic transport is known to be profoundly dysregulated from early stages of neurodegeneration, further aggravating its progressive nature. To understand the contribution of these proteins in MJD, we screened the effect of 34 transport proteins on Ataxin-3 aggregation. Importin-8 (*Ipo8* gene) was selected for further neuropathology and behavior analysis to evaluate its modulatory effect *in vitro* and *in vivo*. Our results showed that *Ipo8* mRNA expression is increased in MJD and that, upon *Ipo8* downregulation, Ataxin-3 aggregation and neuronal dysfunction are reduced *in vitro* and *in vivo*. Furthermore, *Ipo8* silencing was demonstrated to alleviate ataxic traits *in vivo*. Collectively, our findings indicate that Importin-8 may exert its protective effect by modulating NF-kb/p65 and Argonaute-2 proteins, also previously linked to MJD, standing as a promising therapeutic target to delay disease progression.

## Highlights

• Imp8 downregulation reduces Atxn3 aggregation in MJD cellular and animal models
• Ipo8 silencing improves motor performance and gait in Machado-Joseph disease mice
• Imp8 modulates MJD pathogenesis by possibly regulating NF-kB/p65 and Ago2 expression
• Reducing Ipo8 levels may be a promising strategy to delay MJD disease progression

## Graphical abstract

**Figure:**
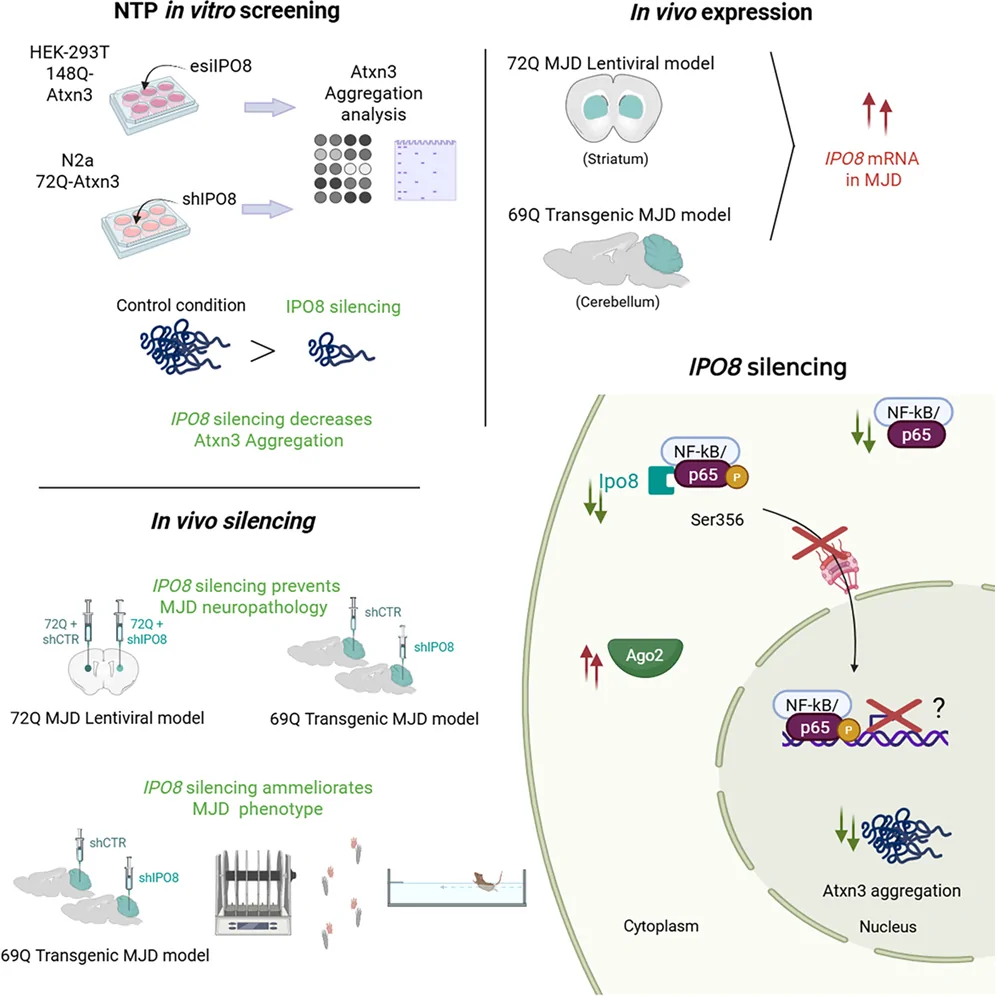

## Introduction

Machado-Joseph disease (MJD), also known as spinocerebellar ataxia type 3, is the most common form of autosomal dominantly inherited ataxias worldwide. It belongs to the group of polyglutamine (polyQ) neurodegenerative disorders.^1^^,^^2^^,^^3^^,^^4^ Despite being rare on a global scale, the disease reaches a particularly high prevalence of 1:239 on the Azorean island of Flores.^1^^,^^2^^,^^5^^,^^6^ The causative mutation, located in the *ATXN3* gene on the chromosome 14q32.1, is characterized by an unstable over-repetition of a CAG sequence that translates into an abnormal polyQ tract within the resulting protein – ataxin-3 (Atxn3).^7^^,^^8^ The polyQ expansion confers toxic properties to Atxn3, turning it prone to form nuclear aggregates in neuronal cells and to induce dysfunction of multiple cellular mechanisms. This sequence of events ultimately results in neuronal death within specific regions of the nervous system.^9^^,^^10^ Some of the most affected brain regions are the cerebellum, brainstem, and striatum, causing clinical hallmarks such as motor incoordination, postural instability, dysarthria, and dysphagia, among other symptoms.^11^^,^^12^^,^^13^ The precise pathological mechanisms underlying MJD remain to be clarified; however, a direct correlation between toxicity and Atxn3 protein aggregates has been demonstrated. Intriguingly, the nuclear localization of the protein appears to be a requisite for the aggregation process.^9^^,^^14^^,^^15^^,^^16^ Other mechanisms such as protein cleavage, autophagy disturbances, neuroinflammation, and transcription dysregulation have also been found to contribute to the disease.^17^^,^^18^^,^^19^^,^^20^^,^^21^

Our previous studies, in conjunction with other parallel research, have already established a link between the modulation of the subcellular localization of Atxn3 and specific nucleocytoplasmic transport proteins.^22^^,^^23^^,^^24^ Nevertheless, various studies have exposed other perspectives of the role of nucleocytoplasmic transport machinery in other neurodegenerative disorders such as amyotrophic lateral sclerosis, frontotemporal dementia, Alzheimer’s disease, and Huntington’s disease, as well as MJD, apart from the localization of the affected protein itself.^22^^,^^25^^,^^26^^,^^27^^,^^28^^,^^29^^,^^30^ These studies demonstrate that a general dysregulation of nucleocytoplasmic transport can not only exacerbate the formation of pathological aggregates but also disrupt the subcellular localization of other macromolecules, including transcription factors and components of other signaling pathways.^31^ The nuclear translocation is a highly regulated process that controls the exchange of protein and RNA molecules between the nucleus and the cytoplasm. This active transport is achieved by karyopherins, also known as importins or exportins, depending on the direction of the transport. These proteins act as receptors that bind to their cargos via nuclear export signals (NESs) and nuclear localization signals (NLSs), and subsequently translocate them through the nuclear pore complex over an active Ran-dependent cycle.^32^^,^^33^ NLS and NES domains have been previously identified in the structure of the Atxn3 protein, reinforcing the hypothesis that it interacts with components of the nucleocytoplasmic transport machinery.^10^^,^^15^^,^^23^

Given the potential for impaired nucleocytoplasmic transport to contribute to the development of various neurodegenerative disorders, our study focused on identifying nucleocytoplasmic proteins that could regulate the aggregation of Atxn3 and potentially reverse the subcellular impairments observed in MJD neuropathology. We performed a screen of 34 proteins based on the overall and up-to-date list of nucleocytoplasmic transport proteins known by the scientific community. The list includes the already known proteins of the two main classes of nucleocytoplasmic transport proteins: importins and exportins. The list also includes co-factors, such as the well-described karyopherin-β1 (KPNB1) and other β-family karyopherins, as well as proteins responsible for the maintenance of the nucleocytoplasmic transport cycle, such as Ran, RanGAP1, and Ran Binding Proteins (RanBP1, 2, or 3).

In this study, we report Importin-8 (IPO8, also known as Ran Binding Protein 8) as an effective modulator of the aggregation process of Atxn3, with no detectable alterations of its subcellular localization. Interestingly, the expression of expanded Atxn3 forms in mouse models appears to induce the expression of *Ipo8* (mouse IPO8 coding gene), while the expression of this gene is relatively lower in wild-type animals, especially in regions affected in MJD, such as the cerebellum. Our *in vitro* and *in vivo* studies demonstrate that *Ipo8* silencing reduces the aggregation of Atxn3, thereby alleviating neuropathological dysfunction, motor coordination, and balance in a transgenic mouse model of severe ataxia. Furthermore, our findings demonstrate that *Ipo8* silencing can not only hinder the development of MJD neuropathological processes associated with Atxn3 aggregation but also potentially modulate its already reported cargos: the Transcription factor p65 (subunit p65 of the NF-kB complex)^34^ associated with neuroinflammation, and the Argonaute RISC catalytic component 2 (Ago2) associated with mRNA silencing and metabolism.^35^^,^^36^ These mechanisms have been previously reported to be dysregulated in MJD.^37^^,^^38^^,^^39^^,^^40^ Our results provide evidence that the nucleocytoplasmic transport mechanisms are indeed relevant in neurodegenerative disorders such as MJD in ways other than just the localization of the affected protein, and that reestablishment of nucleocytoplasmic transport homeostasis may prevent dysfunctions in multiple cellular pathways. Consequently, we emphasize a new contributor to the pathophysiology of MJD and propose a promising therapeutic target to delay disease progression.

## Results

### *IPO8* modulation induces significant changes in Atxn3 aggregation

Previous studies have demonstrated that nucleocytoplasmic transport proteins have a pronounced impact on multiple neurodegenerative disorders.^22^^,^^25^^,^^26^^,^^27^^,^^28^^,^^29^^,^^30^ To investigate whether this class of proteins modulates Atxn3 aggregation and plays a role in MJD pathogenesis, we performed an Ataxin-3 aggregation assay in an HEK-293T MJD cell model using siRNA technology (Figure S1A) to decrease the expression of a selection of nucleocytoplasmic proteins. An siRNA targeting GFP was first used to test its efficiency on the HEK-293T cell model (Figures S1B and S1C), and siCRM1, siTNPO1, and siIPO13 were used for selectivity studies (Figure S1D). A collection of 34 importins, exportins, and other proteins involved in the overall maintenance of the import/export cycle^41^^,^^42^ was then selected for Atxn3 aggregation analysis in a 148Q-Atxn3 transiently expressing HEK-293T cell model (Table S1).

**Figure 1:**
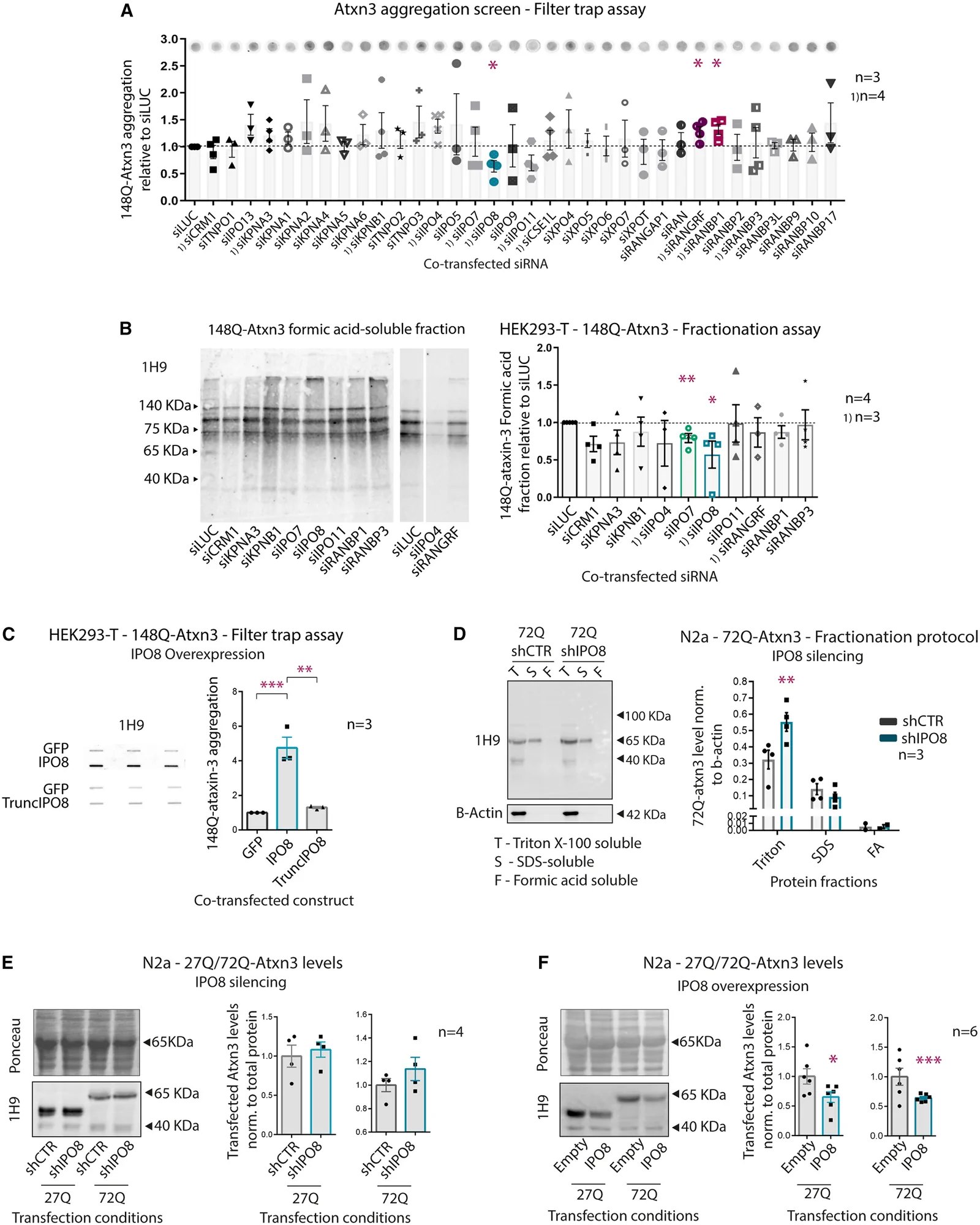
*IPO8* modulation induces significant changes in ataxin-3 aggregation levels (A) Filter trap analysis in HEK-293T cells expresses 148Q-Atxn3 silenced for 34 different transport proteins. siIPO8 (0.6369 ± 0.1058, *p* = 0.0337) significantly decreases 148Q-Atxn3 aggregation while siRANGRF (1.284 ± 0.090, *p* = 0.0449) and siRANBP1 (1.321 ± 0.091, *p* = 0.0312) were shown to increase it. (B) Membrane shows the formic acid fraction of the fractionation protocol to analyze 148Q-Atxn3 aggregation in HEK-293T cells silenced for 11 different transport proteins, highlighting a significant reduction of formic acid-soluble 148Q-Atxn3 when co-transfected with siIPO8 (0.5706 ± 0.1809, *p* = 0.0305) or siIPO7 (0.7918 ± 0.0609, *p* = 0.0059). (C) Filter trap analysis for the quantification of 148Q-Atxn3 aggregation in HEK-293T cells overexpressing IPO8 shows a significant increase of 148Q-Atxn3 aggregation (4.747 ± 0.6185, *p* = 0.0008) in cells co-transfected with full-length IPO8 when compared to cells co-transfected with GFP. When co-transfecting cells with a truncated form of IPO8, the aggregation of 148Q-Atxn3 is significantly reduced (1.300 ± 0.0528, *p* = 0.0012) when compared to full-length IPO8 and closer to the control GFP condition. (D) Fractionation protocol, showing the signal intensities of the protein fractions of N2a cells transfected with 72Q-Atxn3 showing a significant increase in the Triton X-100-soluble levels of ataxin-3 in cells co-transfected with shIPO8 (0.552 ± 0.057, *p* = 0.0068) versus shCTR (0.323 ± 0.058). (E) SDS-PAGE analysis of N2a protein lysates transfected with 27Q or 72Q-Atxn3 and co-transfected with shCTR or shIPO8 for the quantification of Atxn3 levels, showing no statistical difference. (F) SDS-PAGE analysis of N2a protein lysates transfected with 27Q or 72Q-Atxn3 and co-transfected with LV-Empty or LV-IPO8 overexpressing construct, showing a statistically significant decrease of soluble 27Q (IPO8: 0.5095 ± 0.08577; Empty:1.000 ± 0.1196, *p* = 0.0158) and 72Q-Atxn3 levels (IPO8: 0.5135 ± 0.1031; Empty: 1.000 ± 0.1001, *p* = 0.0148) when co-transfected with IPO8. Values are normalized to the respective controls and displayed as mean ± SEM for A to F. ∗*p* < 0.05, ∗∗*p* < 0.01, and ∗∗∗*p* < 0.001; [unpaired *t* test in A, B, E, and F; one-way ANOVA with Tukey’s post hoc test in C; two-way ANOVA with Sidak's Post Hoc test in D].

A filter trap analysis was performed to evaluate the modulatory effects of the chosen siRNAs in 148Q-Atxn3 aggregation (Figure 1A), from some of which the corresponding mRNA silencing efficiency was confirmed and shown in Figure S2. We observed that siRNA-mediated silencing of *IPO8* significantly reduced Atxn3 aggregation, while silencing of RAN guanine nucleotide release factor (*RANGRF)* or RAN binding protein 1 (*RANBP1)* increased it (Figure 1A).

The same samples were prepared to proceed with fractionation analysis previously described by Koch et al.^43^ As previously observed for the filter trap measurements, *IPO8* silencing proved to be a robust strategy to decrease the formic acid-soluble fraction of Atxn3, corresponding to larger and late-stage aggregates (Figure 1B). Moreover, we observed a strong tendency for an increased cellular viability in this model when cells were co-transfected with siIPO8 (Figure S3).

To explore whether *IPO8* overexpression would induce the opposite outcome, the filter trap analysis was performed using the 148Q-expressing HEK-293T cell model co-transfected with a full-length *IPO8* construct (pCMV-SPORT6-IPO8). As expected, *IPO8* overexpression induced a significant increase in 148Q-Atxn3 aggregation (Figure 1C). Interestingly, when co-transfecting the same cell model with a truncated form of *IPO8* lacking its IBB/cargo-recognition domain, this effect was no longer observed (Figure 1C), demonstrating that the modulation of Atxn3 aggregation is specific for IPO8 and dependent on its cargo recognition domain. To investigate *Ipo8* silencing effects in a neuronal model, we used a 72Q-Atxn3 transient expressing N2a cell model co-transfected with shRNAs to specifically silence mouse *Ipo8* (LV-PGK-EGFP-H1-shIPO8). In the N2a MJD cell model, shIPO8 was able to increase the content of Triton X-100-soluble 72Q-Atxn3 in the cells without interfering with the SDS or formic acid-soluble fractions (Figure 1D).

To evaluate whether the effect was due to changes in Atxn3 protein levels, N2a cells were transfected with 27Q or 72Q forms of Atxn3 together with shCTR or shIPO8 and the total protein levels were analyzed by SDS-PAGE (Figure 1E). Upon *Ipo8* silencing, both transfected (27Q or 72Q) and endogenous forms of Atxn3 were unaltered when compared to the control conditions, confirming that *Ipo8* downregulation is not influencing Atxn3 total protein levels (Figure 1E). In addition, when looking at *Atxn3* mRNA levels, we observed no differences neither on its endogenous expression, nor in the N2a cells silenced for *Ipo8*. In addition, no changes were observed *in vivo* using Wt or 69Q Tg animals injected with shCTR or shIPO8 in the cerebellum (Figure S4), supporting that *Ipo8* silencing is not inducing changes in Atxn3 expression.

When overexpressing *IPO8* in the N2a cell model, soluble 27Q and 72Q-Atxn3 protein levels decreased significantly (Figure 1F). We hypothesize that this effect is due to a possible increase in Atxn3 oligomerization or clearance that might prevent its detection on its expected molecular weight. This hypothesis is also supported by the fact that the endogenous mouse Atxn3 protein levels remained unaltered on both *Ipo8* downregulation and upregulation (Figure S5).

Overall, our results suggest that, despite not inducing a clear change in total Atxn3 protein levels and mRNA expression, *Ipo8* downregulation and overexpression modulate Atxn3 solubility significantly. Moreover, results suggest that *Ipo8* silencing may also impact the cellular viability of *in vitro* MJD models positively.

### *Ipo8* mRNA expression is dysregulated in different MJD *in vivo* models

**Figure 2:**
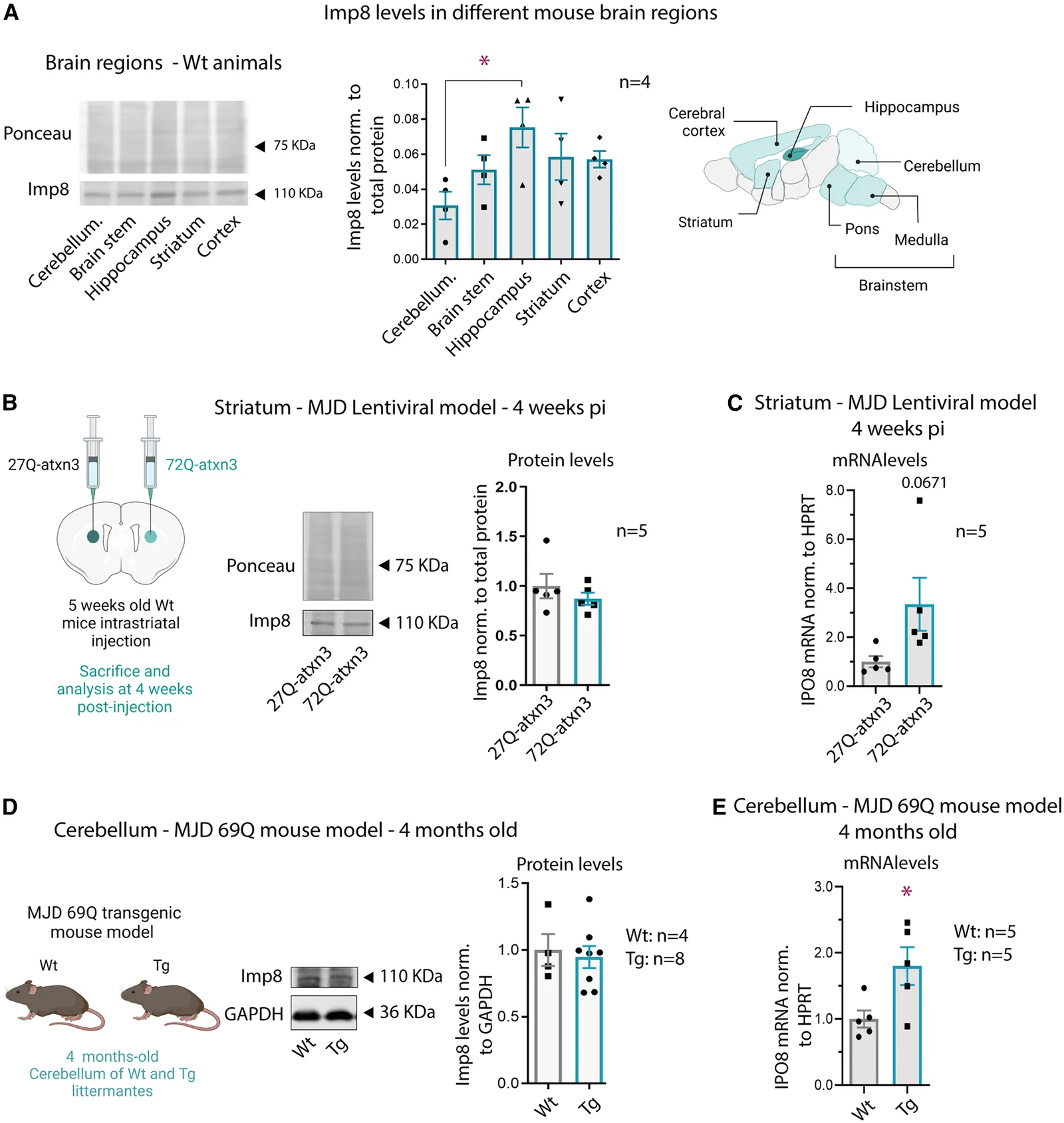
IPO8 is expressed differently in the brain, and IPO8 mRNA is increased in MJD animal models (A) Immunoblot for IPO8 detection in different mouse brain regions and respective histogram, showing increased IPO8 protein levels in the hippocampus when compared to the cerebellum (hippocampus: 0.0753 ± 0.0114 versus cerebellum: 0.0307 ± 0.0079, *p* = 0.0345). (B) Immunoblot for the quantification of IPO8 in the Lentiviral MJD mouse model, comparing protein lysates from one hemisphere injected with lentivirus coding for 27Q-Atxn3 and from the contralateral hemisphere injected with lentivirus coding for 72Q-Atxn3; no significant changes in IPO8 protein levels were found between the two conditions analyzed. (C) qRT-PCR quantification of *Ipo8* mRNA in Lentiviral MJD mouse model injected in the same conditions as in B, showing a tendency for the increase in *Ipo8* expression in the hemisphere injected with 72Q (72Q: 3.344 ± 1.083 versus 27Q: 1.000 ± 0.2297, *p* = 0.0671). (D) Immunoblot analysis of Wt and Tg 69Q mice littermates to quantify IPO8 protein levels in the cerebellum; no change in the IPO8 levels in Tg animals was observed when compared with Wt littermates. (E) qRT-PCR quantification of *Ipo8* mRNA in Wt and 69Q Tg mice, showing a significant increase in *Ipo8* expression in the 69Q Tg animals (Tg: 1.800 ± 0.285 versus Wt: 1.000 ± 0.128, *p* = 0.034); Values are normalized to the respective average of the controls and displayed as mean ± SEM for A-E. One-way ANOVA with Tukey's post hoc test in A; Unpaired *t* test in B, C, D, and E. ∗*p* < 0.05.

We demonstrated that IPO8 stands for an important Atxn3 aggregation modulator. However, to this date, little is known about the expression and distribution of IPO8 in the adult brain and whether its levels are altered in the MJD context. Taking this into account, we evaluated the expression of this protein in different mouse brain regions, namely cerebellum, brainstem, hippocampus, striatum, and cortex of 4-month-old Wt mice by SDS-PAGE. We observed that IPO8 presents relatively higher expression levels in the hippocampus, moderate expression in the brain stem, striatum, and cortex, and reduced expression in the cerebellum (Figure 2A). This data suggests that IPO8 may play different roles throughout the different brain regions and that its expression, at least at its canonical size, may be lower in the cerebellum, one of the most affected brain regions in MJD.

To evaluate whether IPO8 expression is dysregulated in MJD, we quantified differences in IPO8 expression in the lentiviral-induced MJD mouse model expressing human 27Q and 72Q-Atxn3 expression in the striatum,^44^ as well as in the cerebellar expressing Tg 69Q mouse model.^45^^,^^46^ When quantifying IPO8 levels in the lentiviral MJD model, we observed that even showing no changes regarding protein levels (Figure 2B), *Ipo8* mRNA was tendentially increased in the hemisphere injected with 72Q-Atxn3 when compared to the contralateral hemisphere injected with the normal 27Q form of Atxn3, 4 weeks after injection (Figure 2C). Regarding the Tg 69Q mouse model, IPO8 protein was also found to be unaltered in the cerebellum of four months old Tg animals comparing with their Wt littermates (Figure 2D). However, the Tg animals presented a significant increase of *Ipo8* mRNA in the cerebellum at the age of 4 months (Figure 2E) corroborating the results observed in the lentiviral model. Altogether, these results confirm that IPO8 protein is being expressed differently in distinct structures of the adult mouse brain, and its expression is dysregulated in MJD at least at the mRNA level.

### *Ipo8* silencing prevents ataxin-3 aggregation and ameliorates neuropathology in animal models of MJD

Our results showed that *IPO8* silencing reduces Atxn3 aggregation *in vitro*. We further investigated whether this protective effect would be observed *in vivo*, preventing not only Atxn3 aggregation itself, but also neuronal dysfunction and ultimately neuronal death.

**Figure 3:**
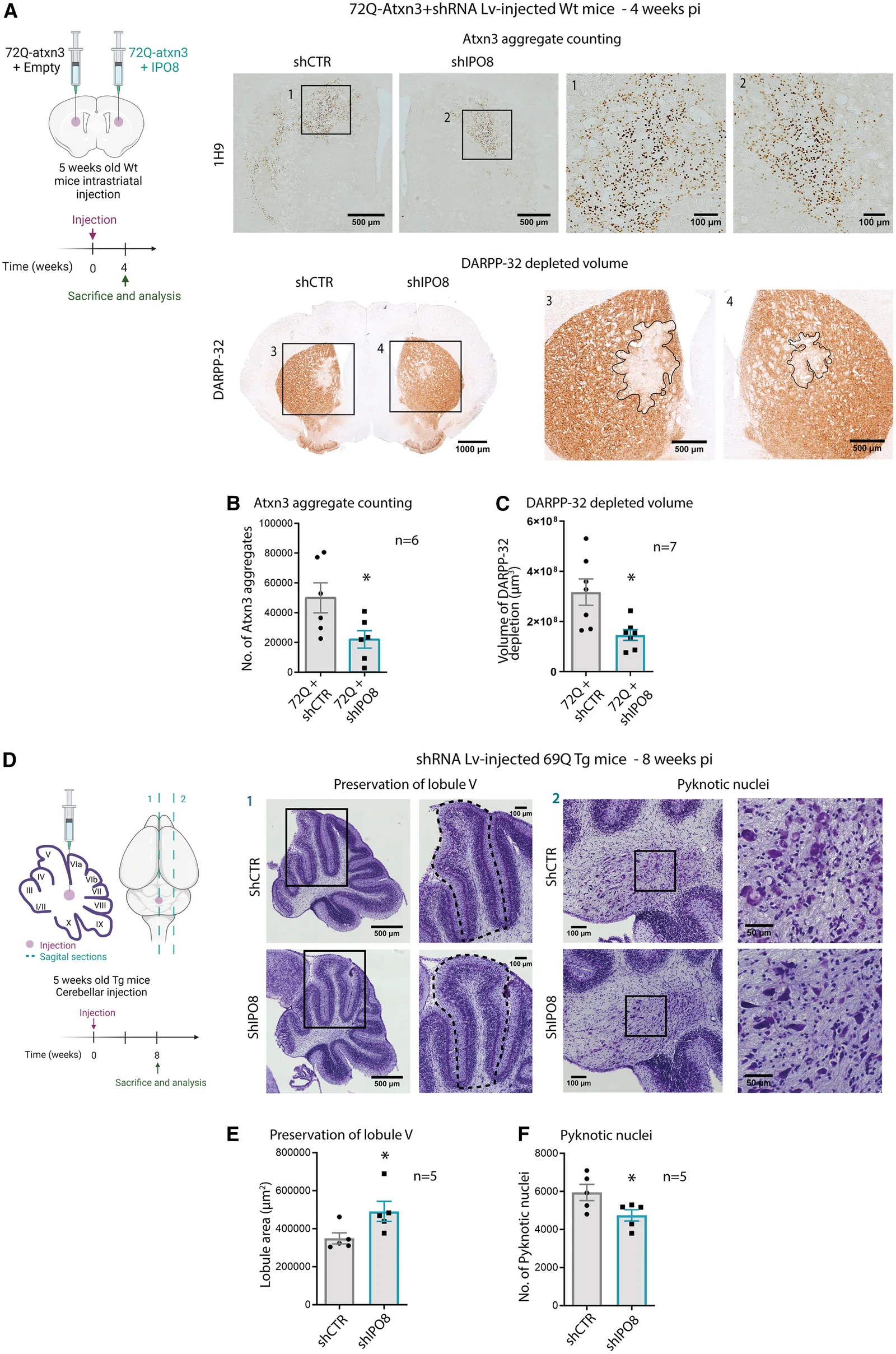
*Ipo8* silencing prevents Atxn3 aggregation and ameliorates neuronal dysfunction *in vivo* (A) Wt animals were stereotaxically co-injected with lentivirus encoding for 72Q-Atxn3 and for shCTR or shIPO8 in the striatum; animals were sacrificed and analyzed at 4 weeks post-injection. Scale bars for 1H9 represent 500 μm, and respective zooms are shown with a scale bar representing 100 μm. Scale bars for DARPP-32 represent 1000 μm, and respective zooms are shown with a scale bar representing 500 μm. (B) Immunostaining of Atxn3 on coronal sections of the striatum of the lentiviral MJD model for the quantification of the number of Atxn3 aggregates - shIPO8 significantly reduced Atxn3 aggregation (shIPO8: 22044 ± 5845 vs. shCTR: 49944 ± 10029, *p* = 0.0371). (C) Immunostaining of the DARPP-32 marker on coronal sections of the striatal lentiviral MJD model for the quantification of its loss of reactivity, showing a significant preservation of the DARPP-32 staining volume on the hemisphere co-injected with shIPO8 lentivirus (shIPO8: 1.466×108 ± 2.123 × 107μm^3^ vs. shCTR: 3.176×108 ± 5.246 × 107μm^3^, *p* = 0.0106). (D) Tg animals were injected in the cerebellum with lentivirus encoding for shCTR or shIPO8 centrally in lobules V-IV and were sacrificed for sagittal brain sectioning 8 weeks post-injection. Sections from two different sagittal planes were selected for cresyl violet staining and analysis: 1 - medial sections covering the injection site for the cerebellar layer measurements (scale bars represent 500 μm and respective zooms are shown with a scale bar of 100 μm) and 2 -sections covering the DCN region for pyknotic nuclei counting (scale bars represent 100 μm and respective zooms are shown with a scale bar of 50 μm). (E) Animals injected with lentivirus encoding for shIPO8 have a significant preservation of the lobule V area when compared to the control animals (shIPO8: 491621 ± 52755 μm^2^ vs. shCTR: 349098 ± 29084 μm^2^, *p* = 0.0455). (F) Animals injected with lentivirus coding for shIPO8 presented a significantly lower number of pyknotic nuclei when compared to control animals (shIPO8: 4747 ± 298 vs. shCTR: 5952 ± 427, *p* = 0.0494). Values are displayed as mean ± SEM for B, C, E, and F. ∗*p* < 0.05 and ∗∗*p* < 0.01; unpaired t-tests.

To do so, we used the lentiviral MJD mouse model expressing the expanded Atxn3 in the striatum, co-injected with lentivirus encoding for shIPO8 or shCTR in the different hemispheres (Figure 3A). Both hemispheres were then compared regarding Atxn3 aggregation rates and expression of the striatal dopamine and CAMP-regulated neuronal phosphoprotein 32 (DARPP-32) as a marker for neuronal dysfunction. We observed a significant reduction in the number of Atxn3 aggregates in the hemisphere co-injected with the shIPO8-encoding lentivirus, compared to the contralateral hemisphere (Figure 3B). Moreover, the same hemisphere showed a significant preservation of the neuronal dysfunction marker DARPP-32, suggesting that *Ipo8* silencing can prevent the loss and maintain functionality of neurons in the striatum (Figure 3C).

We then investigated whether *Ipo8* silencing would preserve neurons in the cerebellum of 69Q transgenic mice, a model characterized by a severe cerebellar atrophy.^45^^,^^46^ 69Q transgenic mice were injected with lentivirus encoding for shCTR or shIPO8 within the cerebellum, and cresyl violet staining was used to highlight the cerebellar structures and to quantify the pyknotic nuclei (Figure 3D). When measuring the area of lobule V, a significant preservation of lobular area was observed in animals injected with shIPO8 coding lentivirus (Figure 3E) indicating a possible cell protecting effect. Additionally, animals silenced for *Ipo8* in the cerebellum showed a significant reduction in the number of pyknotic nuclei in the deep cerebellar nuclei (DCN), demonstrating its capacity to prevent neuronal loss (Figure 3F). These results suggest that *Ipo8* silencing is also neuroprotective *in vivo*, reducing aggregation formation, preventing neuronal dysfunction and neurodegeneration in two different MJD mouse models.

### *Ipo8* silencing alleviates motor deficits in a transgenic mouse model

*Ipo8* silencing was shown to be a relevant strategy to reduce MJD neuropathologic hallmarks both in the mouse striatum and cerebellum. To investigate whether neuropathology recovery would also ameliorate phenotype and delay disease progression, we performed *in vivo* behavioral tests in Wt and 69Q transgenic mice, characterized by profound motor deficits.^45^^,^^46^

**Figure 4:**
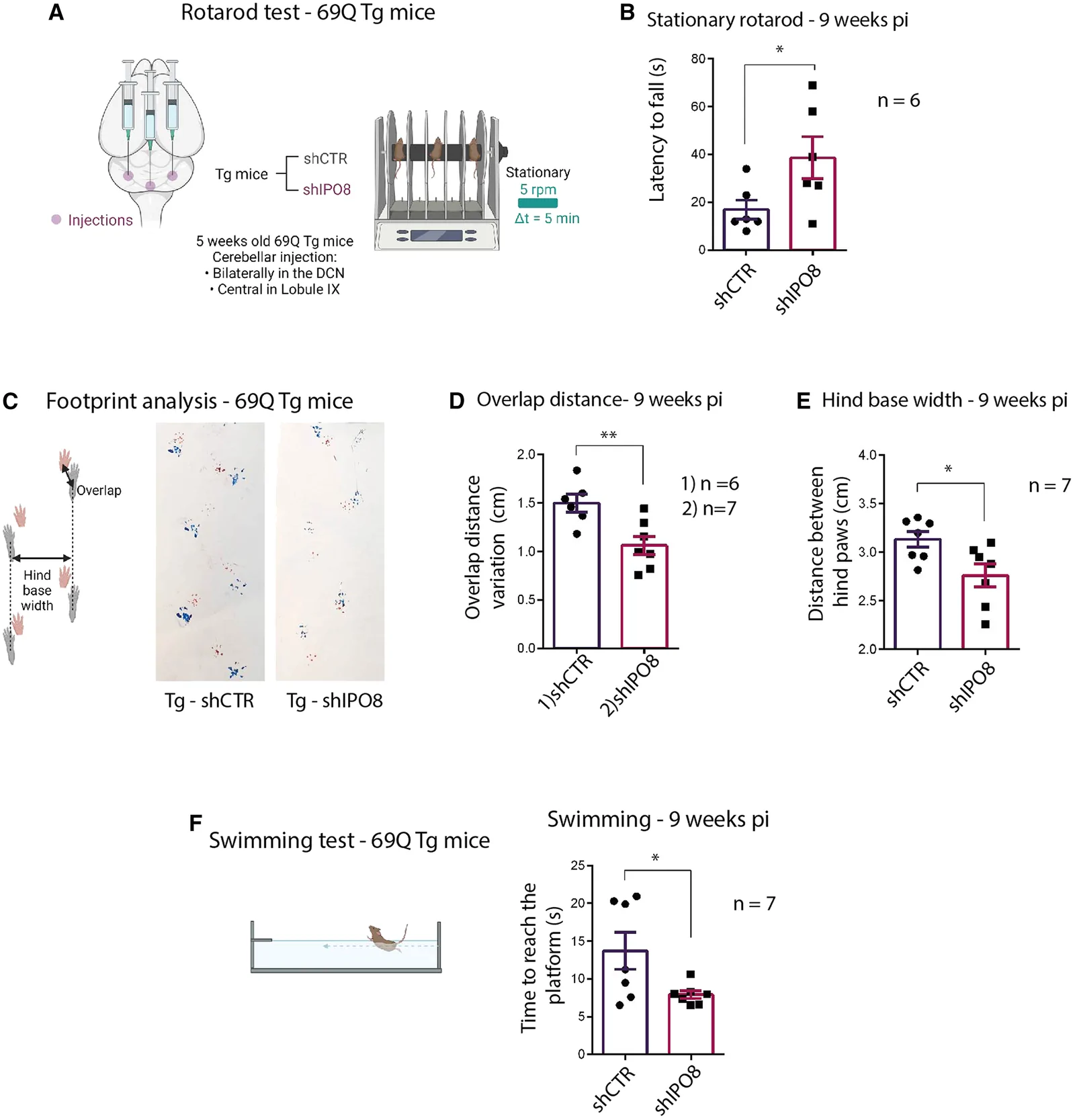
*Ipo8* silencing in the cerebellum alleviates MJD phenotype (A) 69Q Tg animals were injected in the cerebellum (bilaterally in the DCN and centrally in lobules IX-X) with either shCTR or shIPO8-coding lentivirus. Stationary rotarod was performed at 9 weeks post-injection. (B) At 9 weeks post-injection, transgenic animals transduced with shIPO8 held on the rotarod significantly longer compared with Tg animals transduced with shCTR in the stationary mode of the test (shIPO8: 38.67 ± 8.774 s vs. shCTR: 17.00 ± 3.967 s, *p* = 0.0482). (C) Footprint analysis was performed to investigate gait performance (hind and fore paw print overlap and hind base width). (D) 69Q Tg animals transduced with shIPO8 presented a significantly reduced distance between the prints of the fore and hind paws (overlap) (shIPO8: 1.063 ± 0.09364 cm vs. shCTR: 1.498 ± 0.09033 cm, *p* = 0.0069). (E) 69Q Tg animals transduced with shIPO8 presented a significantly reduced distance between the two hind paws (hind base width) (shIPO8: 2.761 ± 0.1188 cm vs. shCTR: 3.134 ± 0.0804 cm, *p* = 0.0235). (F) Performing the swimming test at 9 weeks post-injection, shIPO8-transduced 69Q Tg mice took significantly less time to cross a water tank and reach a safe platform (shIPO8: 7.953 ± 0.5179 s vs. shCTR: 13.73 ± 2.422 s, *p* = 0.0380). Data are represented as mean ± SEM. ∗*p* < 0.05 and ∗∗*p* < 0.01; unpaired *t* test in B, D, E, and F].

We injected Wt and Tg littermates with lentiviral vectors encoding for shCTR or shIPO8 in three regions of the cerebellum (the two DCN and lobule IX, Figure 4A) to potentiate its therapeutic effect (*Ipo8* silencing in Tg animals was verified by qRT-PCR and is shown in Figure S6). We analyzed motor coordination and balance of the Wt animal using the stationary rotarod test, the day before the injections and every three weeks, up to 12-week post-injection. No difference in the performance of the Wt animals was observed throughout the course of the experiment, proving that there is no toxicity associated with the injections, nor with the expression of shIPO8 in the cerebellum (Figure S7).

Tg animals were used to perform the stationary rotarod at 9 weeks post-injection. At this time-point, 69Q Tg mice expressing shIPO8 presented a significantly increased latency to fall compared to the Tg animals expressing shCTR, holding on the rod for twice as long (Figure 4B).

Gait was also assessed by the analysis of the mice footprint pattern (Figure 4C). Transgenic animals injected with shIPO8 presented a significant reduction of the overlapping distance of the paws (Figure 4D) and of the hind base width (Figure 4E), thus demonstrating an improvement of gait performance upon IPO8 silencing. Regarding the swimming test, Tg mice that were injected with shIPO8 lentivirus could cross the water tank and find a safe platform in significantly less time when compared to control animals, showing an improvement of motor coordination (Figure 4F).

### IPO8 is modulating the protein levels of NF-kB/p65 and preventing the nuclear shuttling of its phosphorylated form

We have so far proven that *Ipo8* silencing prevents ataxin-3 aggregation and MJD progression, but the exact mechanism behind this positive effect remains elusive.

Since Atxn3 aggregation has been associated with its nuclear localization, we investigated if the downregulation of *Ipo8* was preventing its nuclear shuttling. 72Q-Atxn3-expressing N2a cells were transfected with shCTR or shIPO8 plasmids, and a stably expressing pEGFP-N2-MJD148 HEK-293T cell model was co-transfected with siLUC or siIPO8 to measure and compare Atxn3 signal intensities in the nuclear and cytoplasmic compartments of the cells (Figures S8A and S8B, respectively). Both MJD *in vitro* models showed no differences in Atxn3 subcellular localization under *Ipo8* silencing conditions. Additionally, a nucleocytoplasmic fractionation protocol was performed to quantify the levels of 72Q-Atxn3 in the nuclear and cytoplasmic compartments. As previously observed in our microscopy analysis, no differences in Atxn3 localization were found upon shIPO8 co-transfection (Figure S9). These results indicate that IPO8 does not influence Atxn3 subcellular localization.

**Figure 5:**
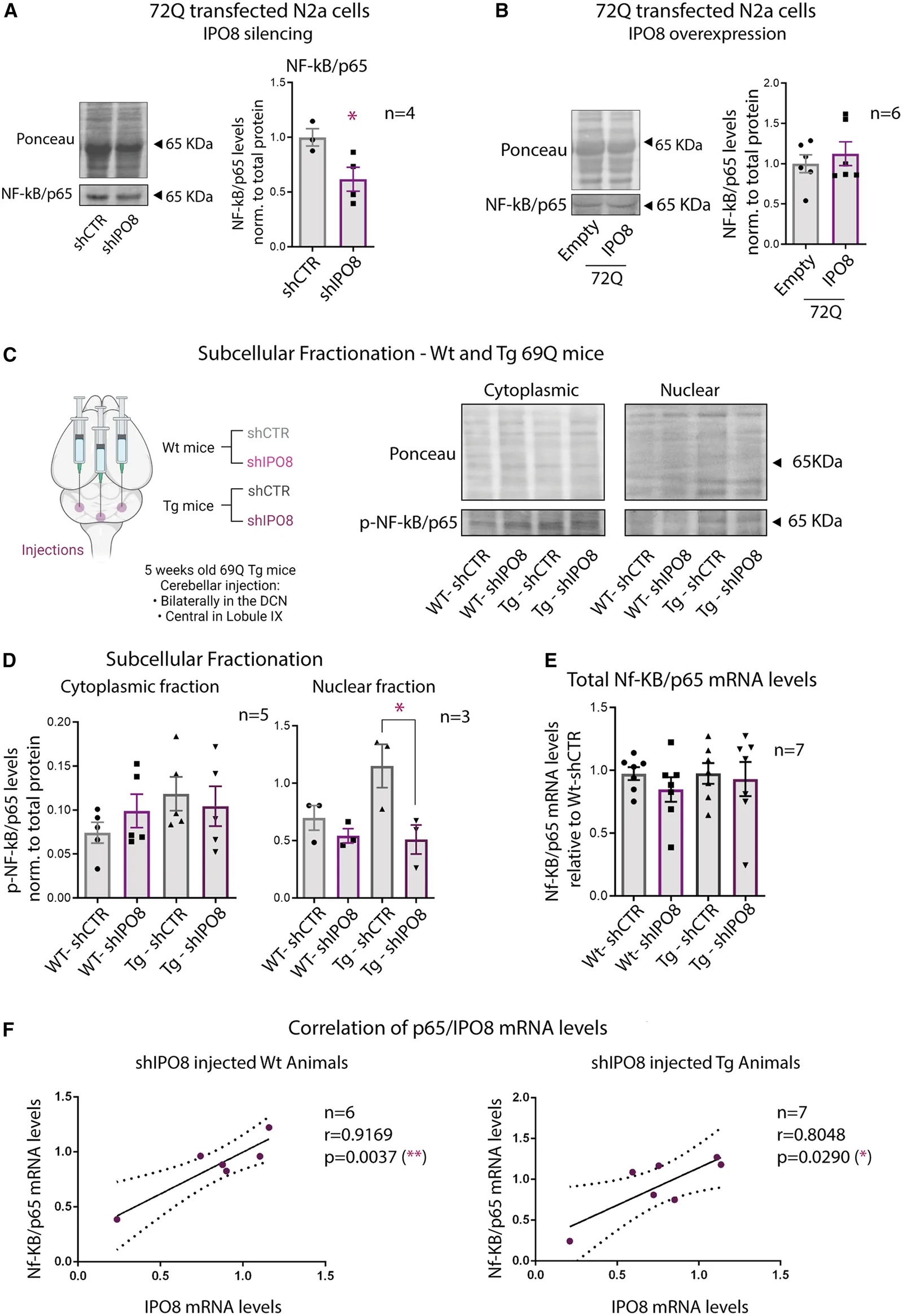
*Ipo8* silencing reduces NF-kB/p65 protein levels and prevents the nuclear shuttling of p-NF-kB/p65 (A) Immunoblot for the detection of NF-kB/p65 total protein levels in 72Q-transfected N2a cells co-transfected with shIPO8 or shCTR. A significant decrease (shIPO8: 0.6178 ± 0.1093 vs. shCTR: 1.000 ± 0.0790, *p* = 0.0464) in NF-kB/p65 protein levels under *Ipo8* silencing conditions was observed. (B) Immunoblot for the detection of total NF-kB/p65 protein levels in 72Q-transfected N2a cells co-transfected with LV-IPO8 or LV-Empty (control). Statistical analysis showed no significant difference in NF-kB/p65 protein levels under these conditions. (C) Immunoblotting analysis of 69Q Tg and Wt animals injected in cerebellum (bilaterally in the DCN and centrally in lobules IX-X) with either shCTR or shIPO8-coding lentivirus. Cerebellar samples collected 12 weeks post-injection were used for the quantification of cytoplasmic/nuclear fractions of Ser356 phosphorylated NF-kB/p65. (D) Results from the subcellular fractionation protocol show no change in the cytoplasmic fraction of p-NF-kB/p65 but a remarkable decrease in the amount of its phosphorylated form in the nuclear compartment of 69Q Tg animals treated with shIPO8 versus Tg animals treated with shCTR (Tg shIPO8: 0.5094 ± 0.1261; Tg shCTR: 1.151 ± 0.1887, *p* = 0.0326). (E) qRT-PCR quantification of NF-kB/p65 mRNA levels in Tg and Wt animals injected in cerebellum (bilaterally in the DCN and centrally in lobules IX-X) with either shCTR or shIPO8-coding lentivirus. No significant differences were found in NF-kB/p65 expression levels among the animal groups. (F) Correlation between *Ipo8* and NF-kB/p65 mRNA levels in animals injected in cerebellum (bilaterally in the DCN and centrally in lobules IX-X) with shIPO8-coding lentivirus, showing a significant and positive correlation for both Wt (*r* = 0.9169; *p* = 0.0037) and 69Q Tg animals (*r* = 0.8048; *p* = 0.0290). Data are represented as mean ± SEM in A, B, D, and E and normalized to the mean of controls for A, B, and E. Unpaired *t* test in A and B; one-way ANOVA with Tukey's post hoc test in D and E; Pearson’s correlation in F. ∗*p* < 0.05 and ∗∗*p* < 0.01.

Our results suggested that IPO8 may be interacting with other molecules, influencing MJD neuropathology. It was previously reported that IPO8 can promote the nuclear transport of the transcription factor p65 (subunit p65 of the NF-kB complex, also known as RelA).^34^ NF-kB/p65 is a transcription factor that, upon activation of the upstream NF-kB pathway, is transported to the nucleus to activate the transcription of other pro-inflammatory cytokines.^34^^,^^47^ Using the same N2a cellular model expressing 72Q-Atxn3 or 27Q-Atxn3, *Ipo8* silencing led to a significant decrease in NF-kB/p65 total protein levels in cells expressing the expanded form of Atxn3 (Figure 5A). This effect was milder in cells expressing the 27Q form of Atxn3 (Figures S10A and S10B).

Assuming that an *in vivo* MJD model would be a better strategy to analyze NF-kB pathway activation, we further evaluated the nucleocytoplasmic distribution of the phosphorylated form of NF-kB/p65 at serine 356, the most studied NF-kB/p65 activating post-translational modification,^48^^,^^49^ using Wt and Tg mice injected with lentivirus encoding for shCTR or shIPO8 (Figure 5B, *in vivo IPO8* silencing efficiency was previously shown in Figure S6). Tg animals presented tendentially more p-NF-kB/p65 accumulated in the nucleus. Importantly, p-NF-kB/p65 levels were significantly reduced in the nuclear fraction of Tg animals injected with shIPO8, when compared to Tg-shCTR mice (Figures 5C and 5D), to levels closer to those observed in Wt animals. To understand if IPO8 silencing would induce a decrease in NF-kB/p65 expression at the mRNA level, we performed qRT-PCR analysis in samples collected from the same animals used for the subcellular fractionation protocol to quantify NF-kB/p65 levels. Despite not being statistically significant, we observed that both Wt and Tg animals injected with shIPO8 lentiviral vectors presented a lower mean NF-kB/p65 expression value (Figure 5E) that positively and significantly correlates with their respective *Ipo8* mRNA expression levels (Figure 5F). These results suggest that *Ipo8* silencing reduces the total protein levels of NF-kBp65 *in vitro*, but also that even a mild *Ipo8 in vivo* silencing would induce a significant and proportional effect on the NF-kB/p65 expression. Moreover, *in vivo Ipo8* silencing can significantly prevent the translocation of the S356 phosphorylated form of NF-kB/p65 to the nucleus.

Overall, our results suggest that the benefits induced by *Ipo8* silencing in MJD neuropathology and behavior might not be due to the prevention of direct transportation of Atxn3 nuclear accumulation, but rather to another neuropathological pathway. We propose that *Ipo8* silencing prevents overactivation of NF-kB-associated neuroinflammation pathways.

### IPO8 is modulating the protein levels of Ago2

Our results suggest that *Ipo8* expression may be dysregulated in the context of MJD, and so, it may also be affecting the expression, function, and/or localization of its interaction partners. We show in the previous section that NF-kB/p65 expression and localization is affected upon silencing *Ipo8*, possibly explaining the positive effects observed *in vitro* and *in vivo* models of the disease. Yet, other IPO8 interacting partners have been described, including argonaute-2 (Ago2). The formation of the IPO8-Ago2 complex is necessary for miRNA recruitment and targeting to specific mRNAs in the cytoplasm.^35^ IPO8 is also believed to interplay with the Ago2 nuclear re-entering of specific miRNAs, where they can regulate the biogenesis and function of other miRNAs or even their own expression.^36^ This transport of mature miRNAs was also reported to take place in a complex with Ago2.

**Figure 6:**
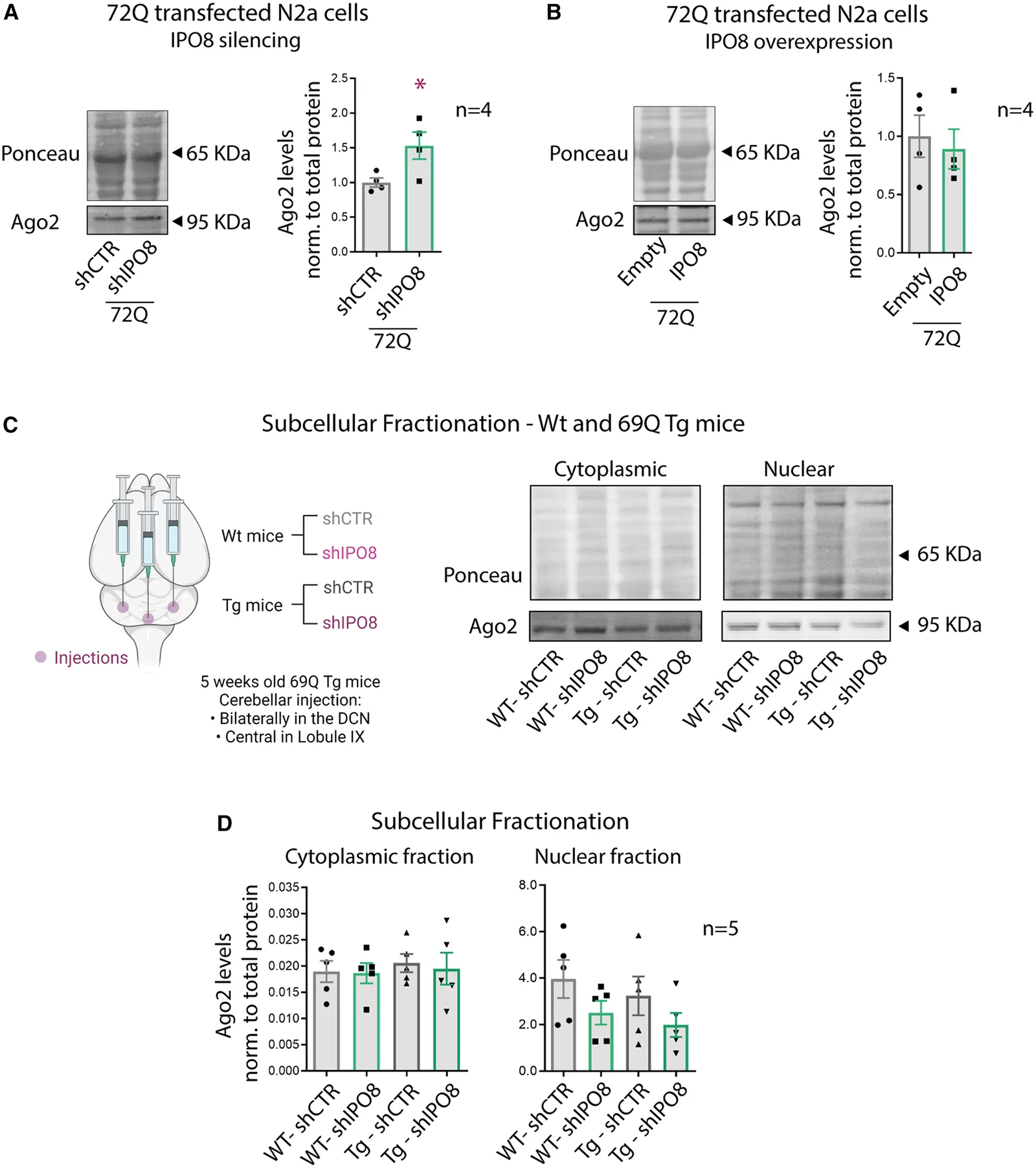
*Ipo8* silencing increases Ago2 protein levels (A) Immunoblot for the detection of Ago2 protein levels in 72Q-transfected N2a cells co-transfected with shIPO8 or shCTR as control. Statistical analysis showed a significant increase in Ago2 protein levels under *Ipo8* silencing conditions (shIPO8: 1.531 ± 0.1941 vs. shCTR: 1.000 ± 0.0667, *p* = 0.0413). (B) Immunoblot for the detection of total protein levels of Ago2 in 72Q-transfected N2a cells co-transfected with LV-IPO8 or LV-Empty construct as control. Statistical analysis showed no significant difference in Ago2 protein levels under *IPO8* overexpression conditions. (C) 69Q Tg and Wt animals were injected in the cerebellum (bilaterally in the DCN and centrally in lobules IX-X) with either shCTR or shIPO8-coding lentivirus. Immunoblotting analysis was performed in cerebellar samples collected 12 weeks post-injection for the quantification of cytoplasmic and nuclear Ago2 levels in the Wt and Tg mice. (D) Histograms obtained by analyzing the subcellular fractionation protocol show no change either in the cytoplasmic or the nuclear fractions of Ago2 protein. Data are represented as mean ± SEM in A, B, and D and normalized to the mean of controls for A and B. Unpaired *t* test in A and B; One-way ANOVA with Tukey's Post Hoc test in D; ∗*p* < 0.05.

We investigated whether the downregulation of Ipo8 modulates the expression of Ago2 and prevents its nuclear shuttling. Using the N2a cellular model expressing 72Q-Atxn3 or 27Q-Atxn3, *Ipo8* silencing led to a significant increase in Ago2 total protein levels in cells expressing the expanded form of Atxn3 (Figure 6A). These effects were milder in cells expressing the 27Q form of Atxn3 (Figure S16). In the same cell models, *IPO8* overexpression appears not to have an effect regarding Atxn3 aggregation rates (Figures 6B and S11).

We then used the *in vivo* MJD model to evaluate the nucleocytoplasmic distribution of Ago2, using Wt and 69Q Tg mice injected with lentivirus encoding for shCTR or shIPO8 (Figure 6C). Neither Wt nor Tg animals presented statistically significantly reduced levels of Ago2 in the nucleus upon the injection of shIPO8. (Figures 6C and 6D).

Our results suggest that the benefits induced by *Ipo8* silencing in MJD neuropathology and behavior may also be associated with the increased levels of Ago2 that are reported to be reduced in MJD models and, thus, possibly establishing important miRNA processing pathways.

## Discussion

In this study, we present evidence showing that the nucleocytoplasmic transport protein IPO8 regulates Atxn3 aggregation and MJD pathology. Specifically, *Ipo8* downregulation prevented Atxn3 aggregation in both MJD cellular and animal models. This led to an amelioration of the neuropathological dysfunction and the ataxic phenotype induced by the expanded PolyQ tract in MJD mouse models. However, further investigation is necessary to elucidate the precise molecular mechanism through which IPO8 influences Atxn3 aggregation. Our findings suggest that IPO8 modulates the expression and translocation of NF-kB/p65 to the nucleus, as well as Ago2 expression, thereby indirectly contributing to the amelioration of MJD pathogenesis.

This study also found that IPO8 protein levels vary in distinct regions of the adult mouse brain. Previous studies have identified the presence of IPO8 in the mouse brain during embryonic development, underscoring its functional significance in the development of the mouse cortex,^50^ but little is known about its function in the adult brain. Databases such as the Human Protein Atlas, GTEx, and Allen describe IPO8 expression overall as ubiquitous and at a low level, with a low region specificity in the adult brain. In parallel, the overall protein level of IPO8 was found to be relatively low in mouse brain lysates in our studies, even though regional differences were significant.

Considering that the cerebellum is among the brain regions most severely affected in MJD and exhibits lower basal levels of IPO8 expression compared to other brain regions, it is pertinent to investigate a potential direct association between IPO8 expression and the modulation of MJD pathogenesis. In our future studies, brain samples from both healthy human and patients with MJD will be available and tested for basal and regional IPO8 protein expression.

In both the lentiviral and the 69Q transgenic MJD models, an increase in *Ipo8* mRNA levels was observed in the striatum and cerebellum, respectively, while the protein level remained unaltered. One potential explanation for this phenomenon is that, in addition to presenting increased *Ipo8* mRNA levels, it may be trapped in mRNA processing bodies, such as stress granules or P-bodies. These structures have been reported to be chronically present and to be part of the pathophysiology of multiple neurodegenerative disorders, including PolyQ disorders.^51^^,^^52^^,^^53^ Increased IPO8 protein turnover may also occur in Tg animals when compared to their wild-type animals, or some IPO8 protein isoforms that are more relevant to the pathology may not be detected on their expected full-length molecular weight. Further research is necessary to comprehend this issue and investigate the potential underlying cause for the dysregulation of *Ipo8* mRNA, and, eventually, of IPO8 protein levels in MJD.

Our *in vivo* MJD neuropathology data indicate that *Ipo8* silencing can reduce Atxn3 aggregation and prevent neuronal dysfunction and death. These parameters were evaluated in two MJD models: the lentiviral model expressing expanded full-length Atxn3 in the striatum, in which dysfunction and loss of DARPP-32-positive cells were prevented; and the 69Q transgenic model expressing an expanded truncated form of Atxn3 in the cerebellum, in which the amelioration of cerebellar atrophy was observed, and the number of apoptotic cells was reduced. A behavioral analysis of 69Q transgenic animals revealed a substantial improvement in motor performance and gait in mice silenced for *Ipo8* in the cerebellum. These findings suggest that reducing *Ipo8* expression in MJD-affected brain regions may serve as a promising strategy to delay the onset and progression of the disease. Our next step will involve employing a more clinically relevant model that better resembles MJD neuropathology, the YAC84Q mouse model. This model expresses the full-length sequence of human Atxn3, including its endogenous regulatory elements, thereby providing an improved platform to elucidate the molecular mechanisms underlying Ipo8 silencing in the context of MJD pathogenesis.

Our subsequent investigation focused on determining whether IPO8 plays a direct role in the transport of Atxn3 through the nuclear membrane. As previously mentioned, the findings of this study suggest that this is not the case. The localization of Atxn3 remained constant in conditions of *Ipo8* downregulation, suggesting that the observed reductions in aggregation levels are not contingent on Atxn3 localization, as previously reported for other transport proteins.^22^^,^^23^ Based on these observations, we postulated that IPO8 might be involved in the transportation of other protein cargos, which exert effects on protein quality control mechanisms, protein aggregation clearance, or the regulation of transcription of other genes. However, the present work can still not rule out the possibility of a direct interaction between IPO8 and Atxn3. Future studies on protein-protein interactions will take place to explore this possibility.

As has been previously documented, IPO8 might be involved in the nuclear transportation of the p65 subunit (also known as RelA) of the NF-kB complex.^34^ NF-κB represents a family of inducible transcription factors that regulate a large collection of genes involved in different processes of the immune and inflammatory responses.^54^^,^^55^ The NF-κB/p65 transcription factor (in complex with other NF-kB subunits) is held in an inactive state in the cytoplasm by binding to its inhibitor IκB. Upon activation, IκB is targeted for degradation, allowing for the phosphorylation of p65 and facilitating its shuttling into the nucleus, where it binds to the promoter regions of other proinflammatory genes.^56^^,^^57^

Previous studies have indicated the involvement of the NF-kB signaling pathways in the pathogenesis of MJD. Ashraf and colleagues have identified multiple genes involved with the NF-kB signaling pathway as regulators of Atxn3 protein expression and, thus, modulating disease pathology.^58^ An interesting link between the alteration of NF-kB/p65 phosphorylation state in MJD was also recently established in our group.^39^ In this study, we demonstrate that *Ipo8* silencing not only reduces NF-kB/p65 total protein levels in the cells, but also significantly reduces the translocation of its Ser356 phosphorylated form to the nucleus, reaching levels that are comparable to those observed in Wt animals.

A previous study on an MJD *Drosophila* model has already reported that the selective inhibition of *Relish*, the fly orthologue of NF-kB, in astrocytes can prevent neuronal degeneration induced by the expression of expanded Atxn3 and increase life span in these animals. This finding indicates that the inflammatory process is an important component of MJD pathology.^59^ A subsequent investigation by our group revealed that the mitigation of neuroinflammation through the administration of ibuprofen can induce neuroprotection, thereby alleviating MJD-associated neuropathology and motor impairments *in vivo*. This finding corroborates the hypothesis that the prevention of neuroinflammation constitutes an efficacious therapeutic strategy to delay disease progression.^38^ Nonetheless, further experiments need to take place to elaborate in detail on the mechanistic causal effects of IPO8 in both the NF-kB signaling pathways and MJD.

To further confirm the effect of IPO8 downregulation in neuroinflammation, forthcoming studies will include the assessment of markers indicative of astrogliosis and microgliosis. For this purpose, the transgenic 84Q YAC mouse model will be utilized in combination with AAV-based vectors encoding shIPO8, thereby enabling a more widespread and constitutive expression of the silencing sequence. This experimental strategy will provide more suitable conditions to detect alterations within the neuroinflammatory context, which intensifies in parallel with the progression of MJD characteristic of this animal model.

It has been documented that IPO8 plays a role in the targeting and function of miRNAs. IPO8 complexes with Ago2 to target miRNAs to their mRNA targets, to direct those mRNAs into mRNA processing bodies (p-bodies) or even to translocate them into the nuclear space, where some mRNAs have been reported to act.^35^^,^^36^ Our findings indicate that silencing *Ipo8* can also increase the expression of Ago2 *in vitro*. Ago2 was previously reported to be downregulated in the 69Q transgenic mouse model.^40^ Therefore, it is hypothesized that *Ipo8* silencing can induce beneficial neuropathology and behavioral effects by restoring Ago2 expression levels and possibly reinstating the normal mRNA silencing activity. With regard to the nucleocytoplasmic translocation of Ago2, our findings were not conclusive since statistical significance was not achieved. Subsequent studies need to be conducted to further investigate the impact of Ago2 subcellular localization on normal and MJD cellular functions.

**Figure 7:**
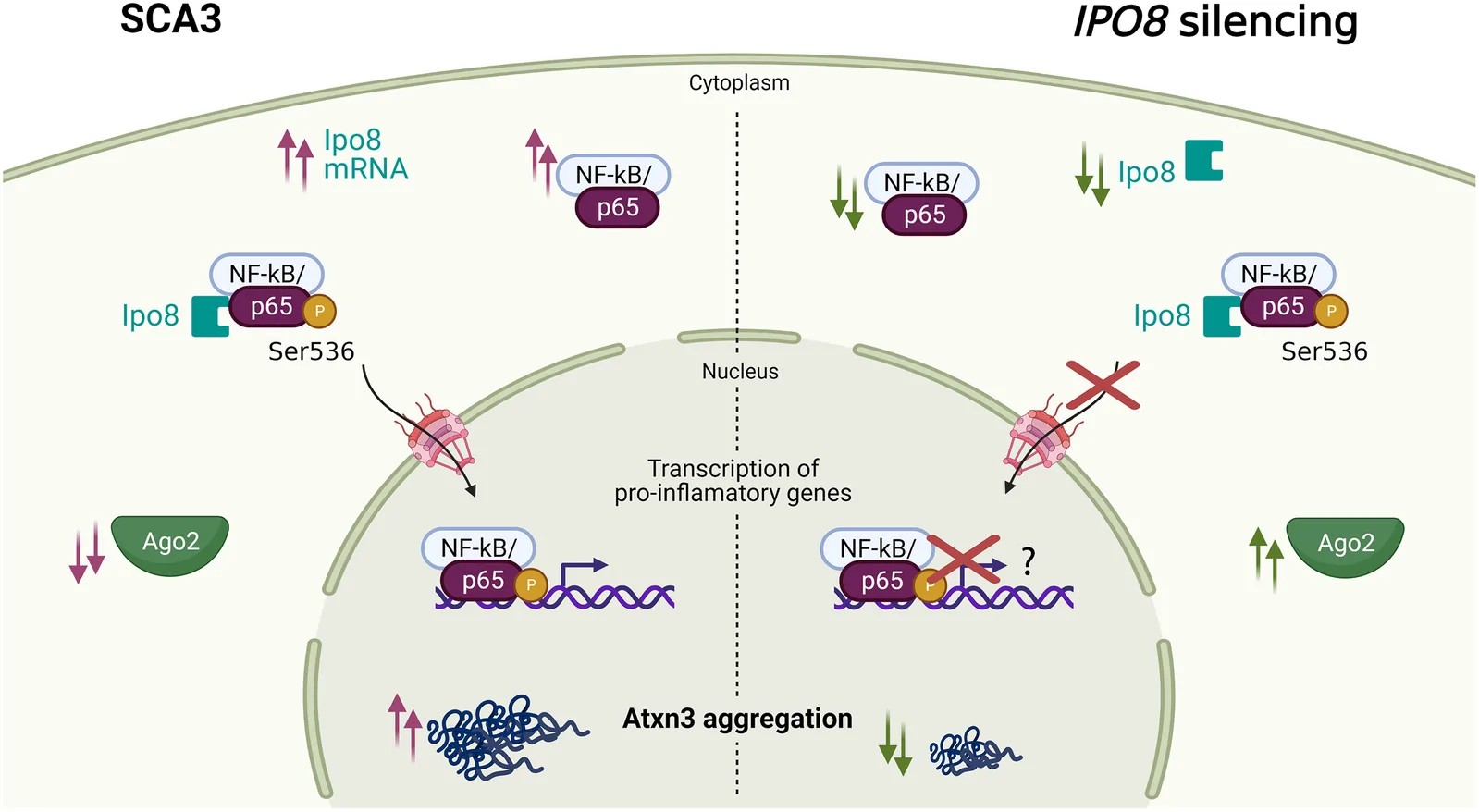
Proposed model for *IPO8* silencing effects on MJD In MJD, *IPO8* mRNA is overexpressed, and IPO8 protein may be contributing to the translocation of the phosphorylated form of NF-kB/p65 to the nucleus to activate the expression of other pro-inflammatory genes, aggravating Atxn3 aggregation and disease pathology. Additionally, Ago2 is reported to be downregulated in MJD. When silencing *IPO8,* the total expression of NF-kB/p65 is decreased, and the translocation of its phosphorylated form to the nuclear space is reduced, possibly preventing the overactivation of the NF-kB pathway. *IPO8* silencing also increases Ago2 protein levels, possibly counteracting the downregulation observed in the disease context. Both mechanisms can account for the amelioration of Atxn3 aggregation and disease progression observed upon *IPO8* silencing in MJD models.

As illustrated in Figure 7, the mechanisms that might contribute to the IPO8-mediated beneficial effects observed in our disease models are outlined.

A comprehensive review of the present work reveals new perspectives in investigating the molecular underpinnings of MJD neuropathology, highlighting IPO8 as a new intervenient in multiple aspects of the disease pathogenesis. This work demonstrated that the suppression of *Ipo8* appears to be an effective strategy for delaying the progression of MJD by means of preventing Atxn3 aggregation and exacerbation of inflammatory processes associated with the disease. Thus, our research offers significant insights into the identification of novel MJD therapeutic targets that have the potential to be extrapolated to other neurodegenerative conditions.

### Limitations of the study

While this study establishes IPO8 as a key regulator of Atxn3 aggregation in MJD, several limitations must be addressed to fully elucidate its therapeutic potential. A primary constraint remains the lack of definitive evidence regarding a direct protein-protein interaction between IPO8 and Atxn3; it is currently unclear whether the observed reduction in aggregation is a direct consequence of their interaction or a secondary effect mediated by the transport of other protein cargos.

Specifically, while our findings suggest that IPO8 modulates the NF-κB/p65 signaling pathway and Ago2 expression, the precise causal mechanisms and the functional impact of Ago2’s subcellular localization require further investigation to achieve statistical and mechanistic clarity. Furthermore, the reliance on lentiviral and truncated 69Q transgenic models necessitates validation in a more physiologically representative platform, such as the YAC84Q mouse model, which better mimics human MJD neuropathology. Finalizing these molecular details and confirming regional protein expression patterns in human MJD brain samples will be critical steps in translating these insights into a viable clinical strategy for delaying disease progression.

## Resource availability

### Lead contact

Luís Pereira de Almeida (luispa@cnc.uc.pt; CNC-Center for Neuroscience and Cell Biology, University of Coimbra, Rua Larga, Coimbra 3004-504, Portugal).

### Materials availability

All unique/stable reagents generated in this study are available from the lead contact upon the completion of a materials transfer agreement.

### Data and code availability

• Data: Data reported in this paper will be shared by the lead contact upon request.
• Code: This paper does not report original code.
• Other Items: Any additional information required to reanalyze the data reported in this paper is available from the lead contact upon request. A list of supplementary data and methods is provided in the following Mendeley Data: https://data.mendeley.com/datasets/s2ng8g5745/1.

## Acknowledgments

European Commission Seventh Framework Programme FP7/2010 under TreatPolyQ -grant no. 264508 (TS, LPA). Federal Ministry of Education and Research - PPPT-MJD Grant 01GM1309B (LPA). COMPETE 2020 - Operational Programme for Competitiveness, and National Funds (LPA). 10.13039/501100001871Foundation for Science and Technology (FCT) - BrainHealth2020 projects CENTRO-01-0145-FEDER-000008 (LPA). Foundation for Science and Technology (FCT) - UID/NEU/04539/2020 (LPA). Foundation for Science and Technology (FCT) - ViraVector CENTRO-01-0145-FEDER-022095 (LPA). Foundation for Science and Technology (FCT) - CortaCAGs PTDC/NEU-NMC/0084/2014 (LPA). Foundation for Science and Technology (FCT) - SpreadSilencing POCI-01-0145-FEDER-029716 (LPA). Foundation for Science and Technology (FCT) - Imagene POCI-01-0145- FEDER-016807 (LPA). Foundation for Science and Technology (FCT) - CancelStem POCI-01-0145-FEDER-016390 (LPA). Foundation for Science and Technology (FCT) - POCI-01-0145-FEDER-032309 (LPA). EU Joint Program-Neurodegenerative Disease Research (JPND) – SynSpread (LPA). EU Joint Program-Neurodegenerative Disease Research (JPND) – ESMI (LPA). EU Joint Program-Neurodegenerative Disease Research (JPND) ModelPolyQ (LPA). EU Joint Program-Neurodegenerative DIsease Research (JPND) – NEURODIET (TS). E-Rare4/0003/2012 Joint Call for European Research Projects on Rare Diseases JTC 2012 (LPA). German Federal Ministry of Education and Research (BMBF, E-Rare project SCA-CYP, grant no. 01GM1803; EuSAge project, grant no. 01DN18020; ELAPSE-ND project, grant no. 01DN23016) (TS). National Ataxia Foundation - USA (TS, LPA). American Portuguese Biomedical Research Fund - APBRF (LPA). Richard Chin and Lily Lock Machado-Joseph Disease Research Fund (LPA). UID/04539/2025. LA/P/0058/2020 (LPA). Foundation for Science and Technology (FCT) - PhD fellowship - PD/BD/114171/2016 (IMM). European Union’s through HORIZON Coordination and Support Actions grants: GeneT- Gene Therapy Center of Excellence Portugal Teaming Project ID:101059981, GeneH Excellence Hub ID:101186939 and GCure Era-Chair ID: 101186929. We thank The Microscopy Imaging Center of Coimbra, at the Center for Neuroscience and Cell Biology (MICC-CNC), for the help and guidance during image acquisitions and Dr. Rosário Faro for the technical support in molecular biology techniques.

## Author contributions

Conceptualization: T.S., L.P.A., and I.M.M.; methodology: T.S., L.P.A., I.M.M., and A.S.S.; investigation: I.M.M., A.S.S., D.R.R., D.D.L., K.L., A.V.F., and D.P.; supervision: T.S. and L.P.A.; funding: T.S., L.P.A., and O.R.; writing: I.M.M.; manuscript revision: I.M.M., A.S.S., D.R.R., D.D.L., K.L., A.V.F., D.P., O.R., T.S., and L.P.A.

## Declaration of interests

The authors declare no competing interests.

## Declaration of generative AI and AI-assisted technologies in the writing process

During the preparation of this work, the author used DeepL and Google Gemini to improve language and readability. After using this tool/service, the authors reviewed and edited the content as needed and take full responsibility for the content of the publication.

## STAR★Methods

### Key resources table

**Table:** 

REAGENT or RESOURCE	SOURCE	IDENTIFIER
**Antibodies**		

Mouse monoclonal anti-Ataxin-3 (clone 1H9)	Merck Millipore	Cat# MAB5360; RRID: AB_2129339
Mouse anti-Importin-8	Santa Cruz	Cat# sc-398854; No RRID assigned
Beta-actin, clone AC-74	Sigma- Aldrich	Cat# A5316; RRID: AB_476743
GAPDH	Merck Millipore	Cat# MAB374; RRID: AB_2107445
Rabbit anti-DARPP32	Merck Millipore	Cat# AB10518; RRID: AB_10807019
Nf-kB/p65, F-6	Santa Cruz	Cat# sc-8008; RRID: AB_628017
phospho Nf-kB/p65	Invitrogen	Cat# MA5-15160; RRID: AB_10983078
Ago-2	Cell Signaling	Cat# 2897; RRID: AB_2096291
Additional Antibodies and dilutions mentioned in supplementary methods	N/A	See Table S2 of supplementary data.

**Chemicals, peptides, and recombinant proteins**		

Attractene Transfection Reagent	Qiagen	Cat# 301005
Polyethylenimine (PEI, MW40000)	Polysciences	Cat# 24885
PrestoBlue Cell Viability Reagent	Thermo Fisher	Cat# A13261

**Critical commercial assays**		

Subcellular Protein Fractionation Kit	Thermo Fisher	Cat# 87790
NucleoSpin RNA Mini Kit	Macherey-Nagel	Cat# 740955.50

**Deposit Data**		

Supplementary and raw data	This paper	https://data.mendeley.com/datasets/s2ng8g5745/1 https://doi.org/10.17632/s2ng8g5745.1

**Experimental models: Cell lines**		

HEK-293T	ATCC	Cat# CRL-3216; RRID: CVCL_0063
Mouse Neuroblastoma (N2a)	ATCC	Cat# CCL-131; RRID: CVCL_0470

**Experimental models: Organisms/strains**		

Mouse: C57BL/6J	Charles River	N/A
Mouse: Tg 69Q (Machado-Joseph Disease model)	Colony established at CNC, University of Coimbra	Torashima et al.^45^ and Oue et al.^46^

**Oligonucleotides**		

shCTR (Oligonucleotide pair)	This study	See Table S2 of supplementary data.
shIPO8_1 (Oligonucleotide pair)	This study	See Table S2 of supplementary data.
shIPO8_2 (Oligonucleotide pair)	This study	See Table S2 of supplementary data.
Primers for qRT-PCR (Human/Mouse)	This study	See Tables S4 and S5 of supplementary data.

**Recombinant DNA**		

Plasmid: *p*-ENTR/pSUPER	AddGene	Cat# 575-1; RRID: Addgene_575-1
Plasmid: SIN-cPPT-PGK-EGFP-WHV-LTR (Gateway destination vector)	This study/Previously described	Alves et al.^44^
Plasmid: pEGFP-N2-148Q-Atxn3	Previously described	Sowa et al.^22^
Plasmid: pCMV-SPORT6-IPO8 (pENTR223.1-IPO8)	Horizon	Cat# OHS5893-202503637
Plasmid: pCMV-SPORT6-TruncIPO8	Horizon	Cat# MHS1010-202699950
Plasmid: PGK-72Q-Atxn3	Previously described	Alves et al.^44^
Plasmid: PGK-27Q-Atxn3	Previously described	Alves et al.^44^
Lentiviral vector: LV-PGK-72Q-Atxn3	Previously described	Alves et al.^44^
Lentiviral vector: LV-PGK-EGFP-H1-shCTR	This study	N/A
Lentiviral vector: LV-PGK-EGFP-H1-shIPO8	This study	N/A

**Software and algorithms**		

GraphPad Prism	GraphPad Software	https://www.graphpad.com/
Image Studio Software	LI-COR	https://www.licorbio.com/image-studio
Zen Lite Blue Edition	Zeiss Microscopy	https://www.zeiss.com/microscopy/en/products/software/zeiss-zen-lite.html
CellProfiler	Broad Institute	https://cellprofiler.org/
BioRender	BioRender.com	https://www.biorender.com/

### Experimental model and study participant details

#### Cell lines

HEK-293T cells were grown at 37°C and 5% CO2 in Dulbecco’s modified Eagle’s medium supplemented with 10% fetal bovine serum, 1% penicillin/streptomycin and 1% nonessential amino acids. Mouse neuroblastoma cells (N2a) were grown at 37°C and 5% CO2 in Dulbecco’s modified Eagle’s medium supplemented with 10% fetal bovine serum and 1% penicillin/streptomycin. To obtain the HEK-293T stably expressing line (data from supplementary section), cells transfected with pEGFP-N2-148Q-Atxn3 were FACS-sorted for highly expressing cells as previously described.^22^ Both cell line were acquired and authenticated from ATCC, and were regularly tested for mycoplasma in our facilities.

#### Animal models

For the MJD lentiviral model, 4 weeks old C57BL/6 female and male mice were injected in the striatum at 5 weeks of age. For cerebellar injections, analysis of neuronal dysfunction and behavioral analysis, 5 weeks old C57Bl/6-background transgenic (Tg) 69Q mice and age-matched wild-type (Wt) mice were used and as previously described.^45^^,^^46^ This 69Q mouse model expresses a 69Q truncated form of ataxin-3 in the purkinje cells of the cerebellum. A colony of the 69Q Tg mice was established at the Center for Neuroscience and Cell Biology of the University of Coimbra and genotyped as described in the Supplementary Methods. Mice were housed under conventional 12 h light–dark cycle in a temperature-controlled room with food and water provided *ab libitum*. For both models, female and male animals were used and the experimental groups were selected to maintain the number littermates and male/female ratio as even as possible. The experiments were carried out in accordance with the European Community directive (2010/63/EU) for the care and use of laboratory animals and were approved by the Responsible Organisation of Animal Welfare of the Faculty of Medicine and Center for Neuroscience and Cell Biology of the University of Coimbra: ORBEA _66_2015/22062015 and ORBEA_289_2021/10122021. The researchers received adequate training (as according to FELASA) and certification to perform the experiments from Portuguese authorities (Direcção Geral da Alimentação e Veterinária, Lisboa, Portugal). All efforts were made to minimize suffering.

### Method details

#### siRNA and shRNA design

MISSION esiRNA (endoribonuclease prepared siRNA) technology was used to silence the expression of targeting NTPs. siGFP (EHUEGFP, Sigma) was used to test efficiency in HEK-293T cells and siCRM1, siTNPO1, siIPO13 and siKPNA3 were used for target selectivity validations. The collection of siRNAs targeting 34 different human transport proteins is listed in Table S1 and was based on the overall up to date list of nucleocytoplasmic transport proteins known by the scientific community. siRNA targeting luciferase was used as control. Further studies were performed using siRNA targeting human *IPO8*.

Regarding shRNA, a control non-targeting short hairpin RNA (shCTR) and two shRNA targeting mouse *Ipo8* mRNA were generated (shIPO8_1 and shIPO8_2). For each, a pair of oligonucleotides was designed. The sequences of each pair of oligonucleotides can be found in Table S3. Each pair of oligonucleotides were annealed and inserted in linearized *p*-ENTR/pSUPER. The H1-shRNA cassette was then transferred with the LR clonase recombination system into SIN-cPPT-PGK-EGFP-WHV-LTR gateway destination vector. Details on shRNA testing are shown in Figure S12. The shIPO8_2 was selected to proceed with further experiments.

#### Aggregate analysis by filter trap and fractionation protocol

For HEK-293T, 1.5x10^5^ cells were plated in six well plates and transfected 24h later with 1 μg of pEGFP-N2-MJD148 Atxn3 + 1 μg siRNA using Attractene Transfection Reagent according to the manufacturer’s instructions. For overexpression studies, 148Q-Atxn3 was co-transfected with 1 μg of the full length expressing human IPO8, that was cloned into a pCMV-SPORT6 backbone through Gateway cloning, or pCMV-SPORT6-TruncIPO8. For N2a, 2x10^5^ cells were plated in six well plates and transfected 24h later with 1 μg PGK-72Q-Atxn3 + 1 μg shRNA using 1 mg/mL polyethylenimine as transfection reagent. An illustration of the used plasmids can be found in Figure S13. Cells from both models were collected 72 h post-transfection in 1x phosphate solution (PBS) containing cOmpleteTM Mini proteinase inhibitor, sonicated 2 × 30 s and protein quantification was determined by Bradford assay according to the manufacturer’s instructions. Filter trap was performed using a cellulose acetate membrane and a filter trap apparatus, as previously described.^22^^,^^60^ The Fractionation protocol was performed as previously described^61^ and adapted to be performed using the same protein samples (Figure S14). Both filter trap and fractionation membranes were detected using the anti-Atxn3, 1H9 primary antibody and the anti-mouse IgG, IRDYE 680RD secondary antibody. Membranes were detected using an Odyssey Fc Imager and quantified using the Image Studio Software.

#### Gene expression

Total RNA from mouse samples was isolated using the NucleoSpin RNA, Mini kit for RNA purification according to the manufacturer’s instructions. cDNA for quantification was obtained by conversion of total RNA with iScript cDNA Synthesis kit according to the manufacturer’s instructions. qRT-PCR was performed in the StepOne Plus Real-Time PCR System (Applied Biosystems) and SsoAdvanced SYBR Green Supermix (Bio-Rad). Primers to detect mouse *Ipo8* cDNA were designed using the ProbeFinder tool version 2.53 (Roche). *Hprt* cDNA quantification was used for sample normalizations. Reactions were performed using a StepOnePlus Real-Time PCR System (Applied Biosystems), using the following cycling conditions: 95°C for 30 s, followed by 45 cycles at 95°C for 5 s and 58°C for 30 s. The amplification rate for each target was evaluated from the cycle threshold (Ct) numbers and differences were calculated using the 2-ΔΔCt method.

#### Immunoblotting analysis

HEK-293T cells were plated at 1,50x10^5^ cells/plate in six well-plates and transfected 24 h after with 1 μg of selected siRNAs using Attractene Transfection Reagent according to the manufacturer’s instructions. Cells were collected 72 h post-transfection in RIPA buffer (RIPA buffer: 50 mM Tris-base; 150 mM NaCl; 5 mM EGTA; 1% Triton X-100; 0.5% sodium deoxycholate; 0.1% SDS) containing cOmpleteTM Mini proteinase inhibitor. Protein quantification was performed by Bradford assay according to manufacture instructions. Western blot was performed as previously described.^62^ Membranes were detected using an Odyssey Fc Imager and quantified using the Image Studio Software. N2a cells were plated at 2.0x10^5^ cells/plate in six well-plates and transfected 24 h later with 1 μg PGK-72Q or 27Q-Atxn3 + 1 μg shRNA using PEI (MW40000, Polysciences). Cells were collected 72 h post-transfection in RIPA buffer containing cOmpleteTM Mini proteinase inhibitor and supplemented with 0.2 mM PMSF (phenylmethylsulphonyl fluoride), 1 mM DTT (dithiothreitol), 1 mM Sodium Orthovanadate and 5 mM Sodium Fluoride. Protein quantification was performed by Bradford assay according to the manufacturer’s instructions. Immunoblots were performed as described.^17^ Membranes were detected using a ChemiDoc Imaging System. All the antibodies and dilutions used for the immunostaining are listed in the key resources table and in de Table S3.

#### Lentiviral vectors production

Lentiviral vectors (Lv) encoding human mutant *ATXN3* (LV-PGK-72Q-Atxn3), negative shRNA (LV-PGK-EGFP-H1-shCTR) and shRNA targeting mouse *Ipo8* mRNA (LV-PGK-EGFP-H1-shIPO8) were produced in HEK-293T cell line with a four-plasmid system, as previously described.^44^^,^^63^ Lentiviral particles were re-suspended in 0.5% BSA in sterile 1x PBS. and quantified by assessing HIV-1 p24 antigen levels by ELISA. Concentrated viral stocks were stored at −80°C until use. Striatal injections of each viral production were performed to confirm transgene expression and evaluate the presence of toxicity (Figure S4). The silencing efficiency of the lentivirus coding for shIPO8 was tested performing stereotaxic cerebellar injections in Wt mice and quantifying mouse *Ipo8* mRNA levels by qRT-PCR (Figure S6).

#### Stereotaxic injection into the striatum and cerebellum

For all the injections, 5 weeks old mice were anesthetized with Ketamine (75 mg/kg) + medetomidine (1 mg/kg) intraperitoneally, with reversion with Antipamezol (2.5 mg/kg) at the end of the surgery. For striatal injections in Wt mice, 400 ng of lentivirus encoding 72Q Atxn3 + 400 ng of lentivirus encoding for shCTR or shIPO8, in a final volume of 2 μL, were stereotaxically co-injected into the striatum in the following coordinates relative to bregma: anteroposterior 0.6 mm, lateral: ±1.8 mm, ventral: 3.3 mm and tooth bar: 0. For cerebellar injections centrally in Lobule V-VI in Wt animals, 800 ng of lentiviral vectors in a final volume of 6 μL encoding shCTR or shIPO8 were stereotaxically injected in the following coordinates relative to Lambda: anteroposterior: −2.4 mm, lateral: 0 mm, ventral: −2.9 mm. Due to reduced brain volume, 69Q Tg animals were injected at: anteroposterior: −2.3 mm, lateral: 0 mm, ventral: −3.0 mm. For behavioral analysis, three injections of 400 ng of shCTR or shIPO8 lentivirus, in a final volume of 6 μL, were injected in the cerebellum of Wt animals using the following coordinates relative to bregma: DCN - anteroposterior: −6.5 mm, lateral: ±0.75 mm, ventral: −2.1 mm; Lobule IX - anteroposterior: −7.75 mm, lateral: 0 mm, ventral: −3.5 mm. For 69Q transgenic mice, the coordinates were the following: DCN - anteroposterior: −6.4 mm, lateral: ±0.7 mm, ventral: −2.0 mm; Lobule IX: anteroposterior: −7.55 mm, lateral: 0 mm, ventral: −3.3 mm.

#### Tissue preparation for histological processing

Mice were anesthetized with an overdose of Ketamine + medetomidine (2.5 × 75 mg/kg + 1 mg/kg, respectively) intraperitoneally. Transcardial perfusion with 1x PBS and fixation with 4% paraformaldehyde (PFA) were performed. The brain was collected and postfixed in 4% paraformaldehyde for 24h and cryoprotected by incubation in 30% sucrose/PBS buffer. Dried brains were then frozen at −80°C. For Wt animals stereotaxically injected in the striatum, 25 μm coronal sections were cut using a cryostat at −21°C. For animals injected in the cerebellum, 30 μm sagittal sections from Wt or Tg mice were cut using the same equipment and conditions. All the sections were stored in 48-well plates containing 1x PBS + Sodium azide to pursue with free-floating immunohistochemistry protocols.

#### Histology

We performed colorimetric immunohistochemistry on serial coronal sections spanning the rostrocaudal extent of the striatum of Wt animals injected with lentivirus encoding 72Q-Ataxin and shCTR/shIPO8. In Wt and Tg animals injected in the cerebellum, cresyl violet staining on sagittal sections through the mediolateral extent of the cerebellum was performed. Details of the histologic procedures, antibodies used for the immunolabeling and for description of the quantifications can be found in the Supplementary Methods. All equivalent sections between the groups were evaluated and treated/imaged under the same conditions. The cresyl violet staining imaging and analysis was performed blindly regarding the injection administered to each animal.

#### Behavior assays

Female and male five-weeks-old mice were submitted to a series of motor tests starting one day before the beginning of the injections and every three weeks until 12 weeks post injection. All tests were performed in a dark room with, at least, one day of acclimatization to the experimental room and at the same period of the day (mouse dark time schedule). Details of each test can be found in the Supplementary Methods. All behavioral tests were performed blindly regarding the genotype and the injection administered to each animal.

##### Stationary rotarod

A Rotarod apparatus Letica Scientific Instruments, model LE 8200 (Panlab, Barcelona, Spain) was used. Rotarod tests were performed as we previously described1. Mice were placed on the rotarod at a constant velocity (5 r.p.m. for a maximum of 5 min). The time during which mice remain running in the rotation roll was recorded. For each test and time point, each animal performed two sets of two trials with at least 30-min inter-trial interval.

##### Swimming test

Mice were placed in the border of a rectangular aquarium (length: 70 cm; height: 20 cm; width: 15 cm), with a platform located at 9 cm above the bottom (length: 7 cm; width: 6 cm). The aquarium was filled up to the area of the platform with water at 24°C–26°C. The time in seconds that mice took to reach the platform was recorded. The time of four trials with an inter-trial interval of at least 30 min were recorded. The first trial was considered as an exploratory trial and the results were expressed as the average of the other three trials.

##### Footprint test

Gait analysis was assessed by the footprint test. To obtain footprints, the hind paws and fore paws of the mice were coated with red and blue non-toxic paints, respectively. Mice were allowed to run along a 100 × 10 × 15 cm blank paper in an apparatus with 15 cm high walls. A fresh sheet of paper was used for each new mouse. Stride length was measured as the average distance of forward movement between each stride. This parameter was determined by the measurement of the perpendicular distance of a given step to a line connecting its opposite preceding and proceeding steps. The distance from left or right front footprint/hind footprint overlap was measured. A perfect overlap, recorded as zero, was considered when the center of the hind footprint fell on the top of the center of the preceding front footprint. When the footprint did not overlap, the distance between the center of footprints was registered. A sequence of five consecutive steps for both hind and forefeet was chosen for evaluation.

#### Nucleocytoplasmic fractionation

For Wt and 69Q Tg mice transduced with either shCTR or shIPO8 coding lentivirus, half of the cerebellum was used to proceed with the nucleocytoplasmic fractionation protocol using the Subcellular Protein Fractionation Kit for Tissues, according to the manufacturer’s instructions. The analysis of fraction purity is shown in Figure S6.

#### cDNA synthesis for NTP mRNA quantification

For real-time RT-PCR quantifications, total RNA was isolated using commercially available RNAeasy kit (Qiagen) according to the manufacturer’s instructions. Complementary DNA (cDNA) was then obtained by conversion of total RNA with QuantiTect Reverse Transcription Kit (Qiagen) according to the manufacturer’s instructions and stored at-20ᵒC. Gene expression was determined by quantitative real-time RT-PCR as described.(*63*) See Table S4 for details regarding the primer sequences for cDNA amplification. Appropriate negative controls were also included. qRT-PCR reactions were carried out in triplicates using SYBR Green according to the manufacturer’s instructions in 384 well plates. A Light Cycler 480 was used to perform the real-time PCR analysis using the following protocol: 45 cycles of 15 s at 94°C (denaturation), 30 s at 55°C (annealing) and 30 s at 72°C (extension); Signal acquisitions were recorded during the extension phase, one time per cycle. Gene expression levels were normalized to the expression of PDH, β-actin and YWHAZ housekeeping genes.

#### Immunohistochemistry

After the blockage of endogenous peroxidases with 0.1% phenylhidrazyne/PBS, free flotting sections were incubated with blocking solution containing 0.1% Triton X-100 in 1% PBS supplemented with 10% normal goat serum. Sections were then incubated overnight at 4°C in blocking solution with primary antibodies: mouse monoclonal anti-Atxn3 antibody (1H9; 1:5000; #5360 Merck Millipore), rabbit anti-dopamine and cyclic AMP-regulated neuronal phosphoprotein 32 (DARPP32) antibody (1:1000; AB#10518 Merck Millipore); followed by incubation with respective biotinylated secondary goat anti-mouse or anti-rabbit antibodies (1:250; Vector Laboratoires). Bound antibodies were visualized using the VECTASTAIN ABC kit, with 3′,3′-diaminobenzidine tetrahydrocloride (DAB metal concentrate; Pierce) as substrate. Sections were then mounted in gelatin-coated slides, dehydrated with ethanol 70%, 96% and 100%, 3 min each solution, xylene solution for 5 min and mounted using EprediaTM mounting medium (Fisher Scientific, no. 4112). Immunoreactivity of mouse sections was analyzed as previously described.3 Staining was visualized with on a Zeiss Axio Imager 2 using the AxioVision 4.7 software package (Carl Zeiss Microimaging).

#### Viability assay

HEK-293T cells were plated in 96-well plates at 1x10^4^ cells/plate and transfected 24h later with 0.1 μg of pEGFP-N2-MJD148 + 0.1 μg siRNA using Attractene Transfection Reagent (Qiagen) according to the manufacturer’s instructions. 72h after cell transfection, cells were washed with pre-warmed 1x PBS and incubated with PrestoBlue™ Cell Viability Reagent (Life Technologies) diluted 1:10 in pre-warmed cell culture medium and incubated 10 min at 37°C, as suggested by the manufacturer’s instructions. Fluorescence values were read using a Synergy HT Microplate Reader under an excitation of 530 nm and 25 nm bandwidth and Emission of 590 nm and 35 nm bandwidth.

### Quantification and statistical analysis

Statistical significance was determined using Student’s *t* test or ANOVA (one-way or two-way) followed by the adequate post hoc test via GraphPad Prism. Individual data points are displayed in all figures to ensure transparency. Results are expressed as mean ± SEM. Significant thresholds were set at *p* < 0.05 (∗), *p* < 0.01 (∗∗)and *p* < 0.001 (∗∗∗) and *p*-values are specified in the figure legend descriptions.
