# Supplementary material for: Importin-8 silencing reduces Ataxin-3 aggregation and alleviates Machado-Joseph disease/spinocerebellar ataxia type 3
Source: iScience. 2026 Aug 26;29(9):116446. doi: 10.1016/j.isci.2026.116446 (PMC13544303; doi:10.1016/j.isci.2026.116446)

**iScience, Volume *29***

## Supplemental information

# Importin-8 silencing reduces Ataxin-3 aggregation

# and alleviates Machado-Joseph

# disease/spinocerebellar ataxia type 3

**Inês Morgado Martins, Anna Sergeevna Sowa, David Rufino-Ramos, Diana Duarte Lobo, Kevin Leandro, Ana Vasconcelos-Ferreira, Dina Pereira, Olaf Rieβ, Thorsten Schmidt, and Luís Pereira de Almeida**

# Supplementary Data S1/Methods S1

This document contains the supplementary figures, tables, and experimental data supporting the optimization, validation, and functional analysis of IPO8 modulation in models of Machado-Joseph Disease (MJD). The document concludes with the complete, uncropped raw images of all gels and Western blots.

**Optimization and validation of siRNAs**

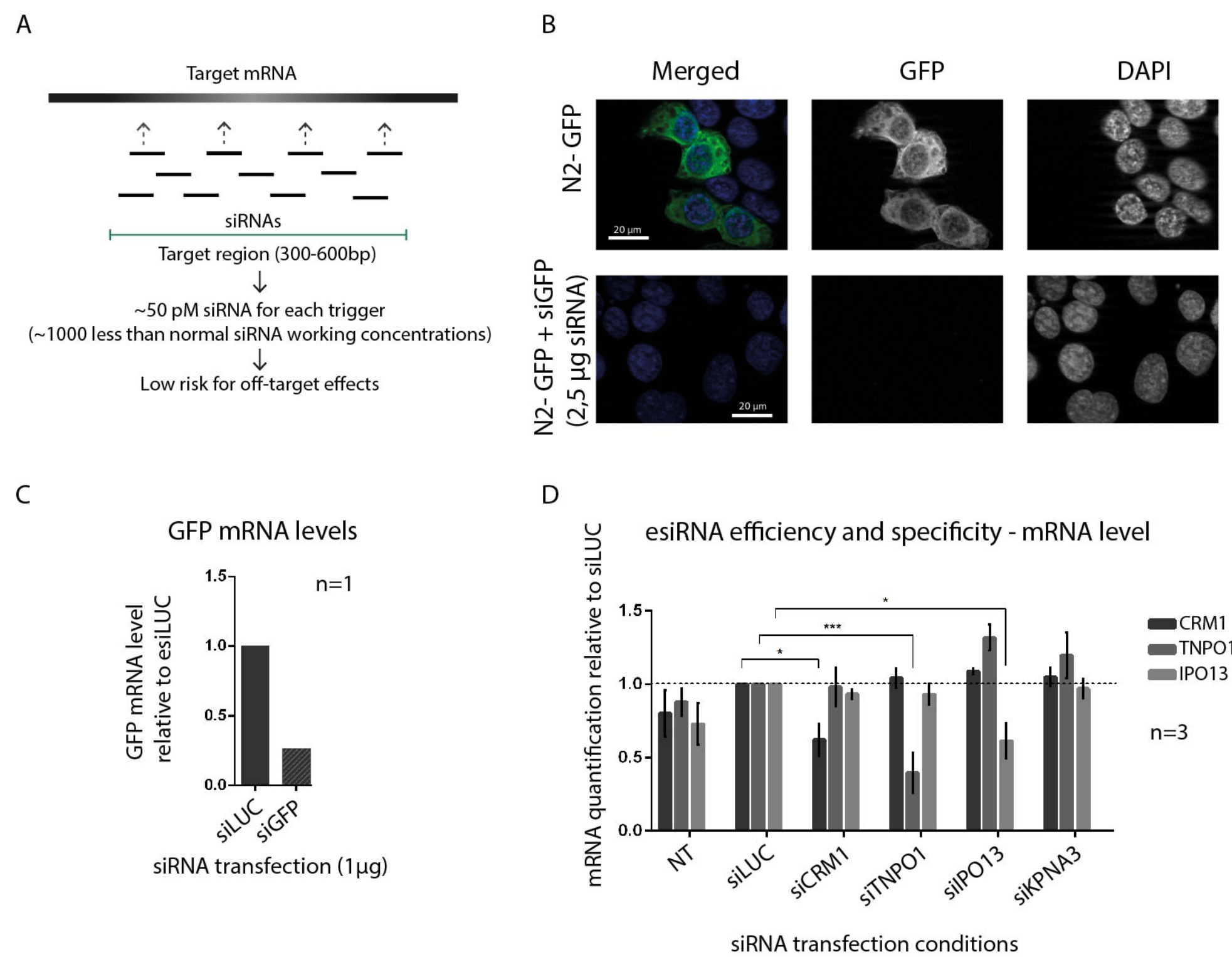


**Fig. S1 – Optimization of siRNAs:** (A) schematic illustration of the siRNA mechanism – each siRNA consists of a pool of several siRNAs targeting the same mRNA but in different regions of that mRNA, allowing the use of lower concentration of each siRNA, lower off-target effects and a very efficient silencing; illustration adapted from Sigma; (B) For siRNA testing, we first used siGFP in HEK-293T cells transfected with an empty N2-eGFP expressing plasmid, to preform microscopy analysis; at a dose of 2,5µg, siGFP showed to be an efficient silencer; Scale bar representing 20µm; (C) We then tested a lower dose of 1µg siGFP in the same cells expressing GFP. By quantitative qRT-PCR, the

expression of GFP was shown to be approximately 75% decreased. (D) To test specificity, HEK-293T cells were transfected with siRNAs targeting CRM1, TNPO1, IPO13 and the endogenous mRNA levels of these proteins were quantified; The mRNA levels of these targets were significantly lower only in the conditions where the respective siRNA was used. Data are represented as mean +/- SEM.

**Table S1 - List of the selected siRNA to perform the filter trap aggregation screen, respective targeting mRNA and ID references.**

| siRNA | Targeting Protein/mRNA | Other names | Ref.ID | Ref.ID |
|---|---|---|---|---|
| *siCRM1* | Chromosome Region Maintenance 1 Homolog (Yeast) | Exportin-1 (XPO1) | HU-03475-1 | ENSG00000082898 |
| *siTNPO1* | Transportin-1 (*TNPO1*) | Importin-B2 (IPO2), Kariopherin Beta 2 (KPNB2) | HU-11314-1 | ENSG00000083312 |
| *siIPO13* | Importin-13 (*IPO13*) | Ran-binding protein 13 (RanBP13) | HU-03029-1 | ENSG00000117408 |
| *siKPNA3* | Karyopherin Alpha 3 (*KPNA3*) | Importin Alpha 4 (IPOA4) | HU-03874-1 | ENSG00000102753 |
| *siKPNA1* | Karyopherin Alpha 1 (*KPNA1*) | Importin Alpha 5 (IPOA5) | HU-01771-1 | ENSG00000114030 |
| *siKPNA2* | Karyopherin Alpha 2 (*KPNA2*) | Importin Alpha 1 (IPOA1) | HU-11118-1 | ENSG00000182481 |
| *siKPNA4* | Karyopherin Alpha 4 (*KPNA4*) | Importin Alpha 3 (IPOA3) | HU-11179-1 | ENSG00000186432 |
| *siKPNA5* | Karyopherin Alpha 5 (*KPNA5*) | Importin Alpha 6 (IPOA6) | HU-01687-1 | ENSG00000196911 |
| *siKPNA6* | Karyopherin Alpha 6 (*KPNA6*) | Importin Alpha 7 (IPOA7) | HU-01702-1 | ENSG00000025800 |
| *siKPNB1* | Karyopherin Beta 1 (*KPNB1*) | Importin-1 (IPO1) | HU-04315-1 | ENSG00000108424 |
| *siTNPO2* | Transportin-2 (*TNPO2*) | Importin-3 (IPO3), Karyopherin Beta 2 (KPNB2) | HU-08652-1 | ENSG00000105576 |
| *siTNPO3* | Transportin-3 (*TNPO3*) | Importin-12 (IPO12) | HU-15803-1 | ENSG00000064419 |
| *siIPO4* | Importin-4 (*IPO4*) | Ran-binding protein 4 (RanBP4) | HU-08329-1 | ENSG00000196497 |
| *siIPO5* | Importin-5 (*IPO5*) | Ran-binding protein 5 (RanBP5), Karyopherin Beta 3 (KPNB3) | HU-05973-1 | ENSG00000065150 |
| *siIPO7* | Importin-7 (*IPO7*) | Ran-binding protein 7 (RanBP7) | HU-11835-1 | ENSG00000205339 |
| *siIPO8* | Importin-8 (*IPO8*) | Ran-binding protein 8 (RanBP8) | HU-00026-1 | ENSG00000133704 |
| *siIPO9* | Importin-9 (*IPO9*) | Ran-binding protein 9 (RanBP9) | HU-10233-1 | ENSG00000198700 |
| *siIPO11* | Importin-11 (*IPO11*) | Ran-binding protein 11 (RanBP11) | HU-13072-1 | ENSG00000086200 |
| *siCSE1L* | Chromosome Segregation 1 Like | Exportin-2 (XPO2) | HU-13369-1 | ENSG0000012420 |
| *siXPO4* | Exportin-4 (*XPO4*) | - | HU-05379-1 | ENSG00000132953 |
| *siXPO5* | Exportin-5 (*XPO5*) | Ran-binding protein 21 (RanBP21) | HU-05399-1 | ENSG00000124571 |
| *siXPO6* | Exportin-6 (*XPO6*) | Ran-binding protein 20 (RanBP20) | HU-03442-1 | ENSG00000169180 |
| *siXPO7* | Exportin-7 (*XPO7*) | Ran-binding protein 16 (RanBP16) | HU-02589-1 | ENSG00000130227 |
| *siXPOT* | Exportin-T (*XPOT*) | Exportin-3 (XPO3) | HU-10641-1 | ENSG00000184575 |
| *siRAN* | Ras-related nuclear protein (*RAN*) | Androgen Receptor-Associated Protein 24 (ARA24) | HU-10457-1 | ENSG00000132341 |
| *siRANGAP1* | Ran GTPase Activating Protein 1 | - | HU-09172-1 | ENSG00000100401 |
| *siRANGRF* | RAN Guanine Nucleotide Release Factor (*RanGRF*) | MOG1 Homolog | HU-12776-1 | ENSG00000108961 |
| *siRANBP1* | Ran-binding protein 1 (*RanBP1*) | Ran-Specific GTPase-Activating Protein | HU-14719-1 | ENSG00000099901 |
| *siRANBP2* | Ran-binding protein 2 (*RanBP2*) | Nuclear Pore Complex Protein Nup358 (NUP358) | HU-01326-1 | ENSG00000153201 |
| *siRANBP3* | Ran-binding protein 3 (*RanBP3*) | - | HU-07616-1 | ENSG00000031823 |
| *siRANBP3L* | Ran-binding protein 3 like (*RanBP3L*) | - | HU-05422-1 | ENSG00000164188 |
| *siRANBP9* | Ran-binding protein 9 (*RanBP9*) | Ran Binding Protein In The Microtubule Organizing Center, Ran-Binding Protein M (RanBPM) | HU-09054-1 | ENSG00000010017 |
| *siRANBP10* | Ran-binding protein 10 (*RanBP10*) | - | HU-02358-1 | ENSG00000141084 |
| *siRANBP17* | Ran-binding protein 17 (*RanBP17*) | - | HU-14202-1 | ENSG00000204764 |

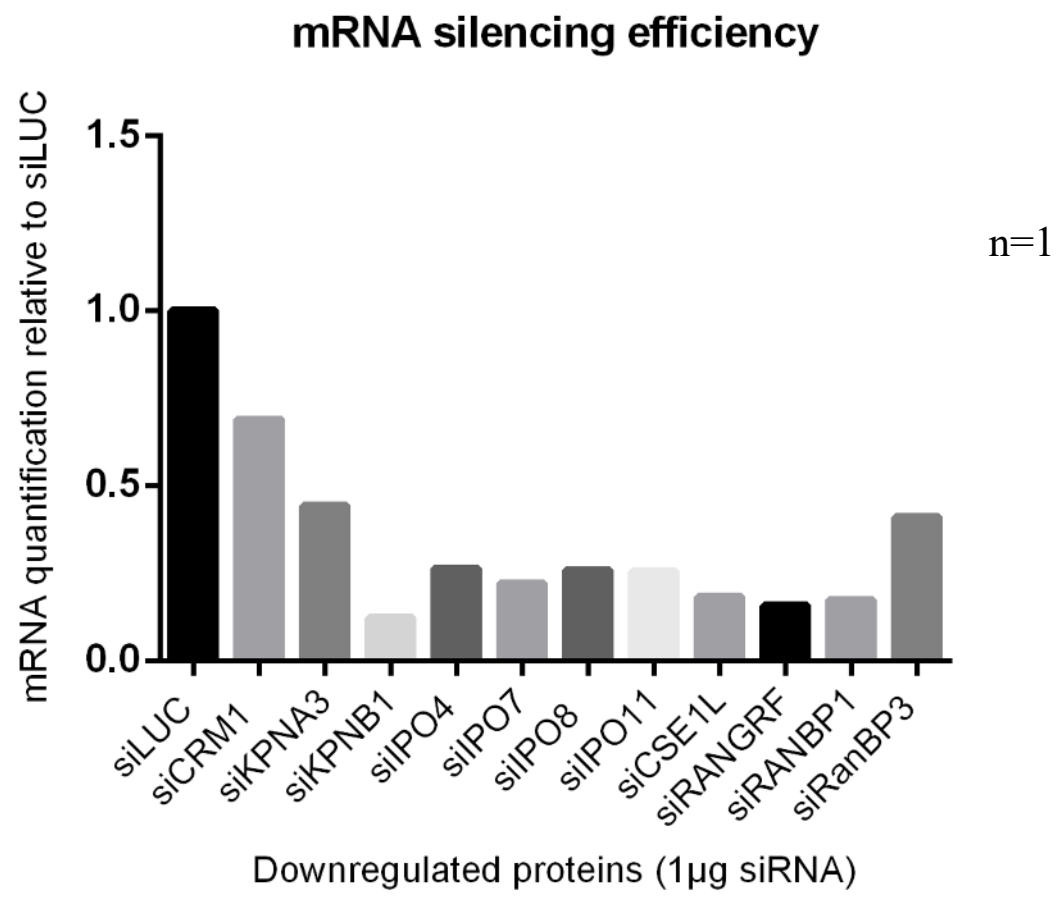


**Fig. S2 – Quantification of the mRNA silencing rate achieved by selected NTP-targeting siRNA transfection in HEK-293T cells:** HEK-293T cells were transfected with 1µg of siLUC or siRNAs targeting 9 of the 12 selected transport proteins from the screen (KPNB1, IPO4, IPO7, IPO8, IPO8, IPO11, CSE1L, RANGRF, RANBP1 and RANBP3; siCRM1 and siKPNA3 testing was performed while testing general siRNA efficiency and specificity and is illustrated in Supplementary Figure 1); Cells were collected 48h post-transfection to be analyzed by real time qRT-PCR for the expression of each nucleocytoplasmic transport-targeting mRNA. Each quantification was calculated relative to the respective transport protein mRNA, on the siLUC (control) condition.

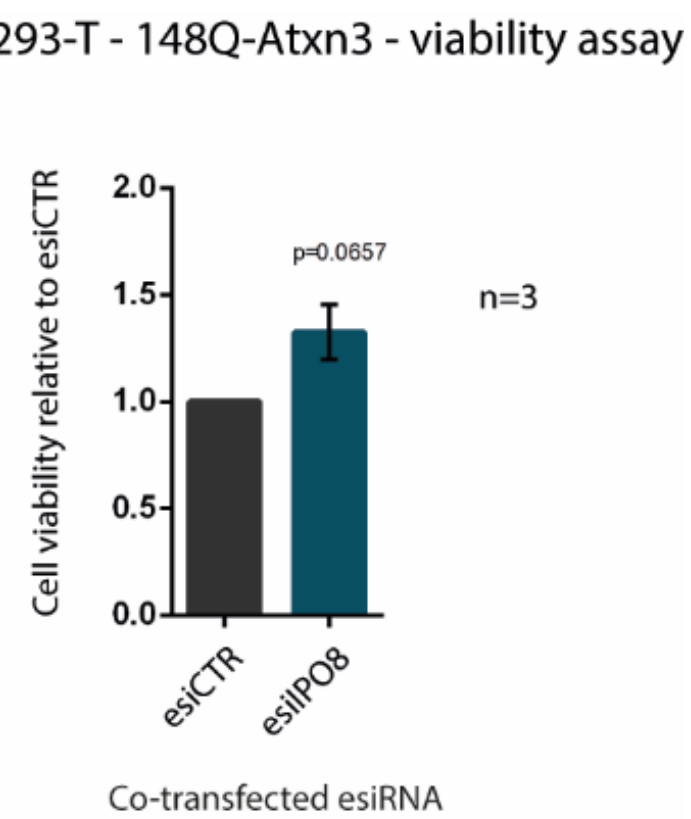


**Fig. S3 - PrestoBlue viability assay for analysis of cell proliferation and survival in 148Q-trasnfected HEK-293T:** cells treated with either siIPO8 or siLUC, showing a tendency for increased viability in the siIPO8 treated cells (siIPO8: 1.326±0.1297, *p*=0.0657) relative to cells treated with siLUC. PrestoBlue procedures are described in the Supplementary Methods. Data is represented as mean +/- SEM.

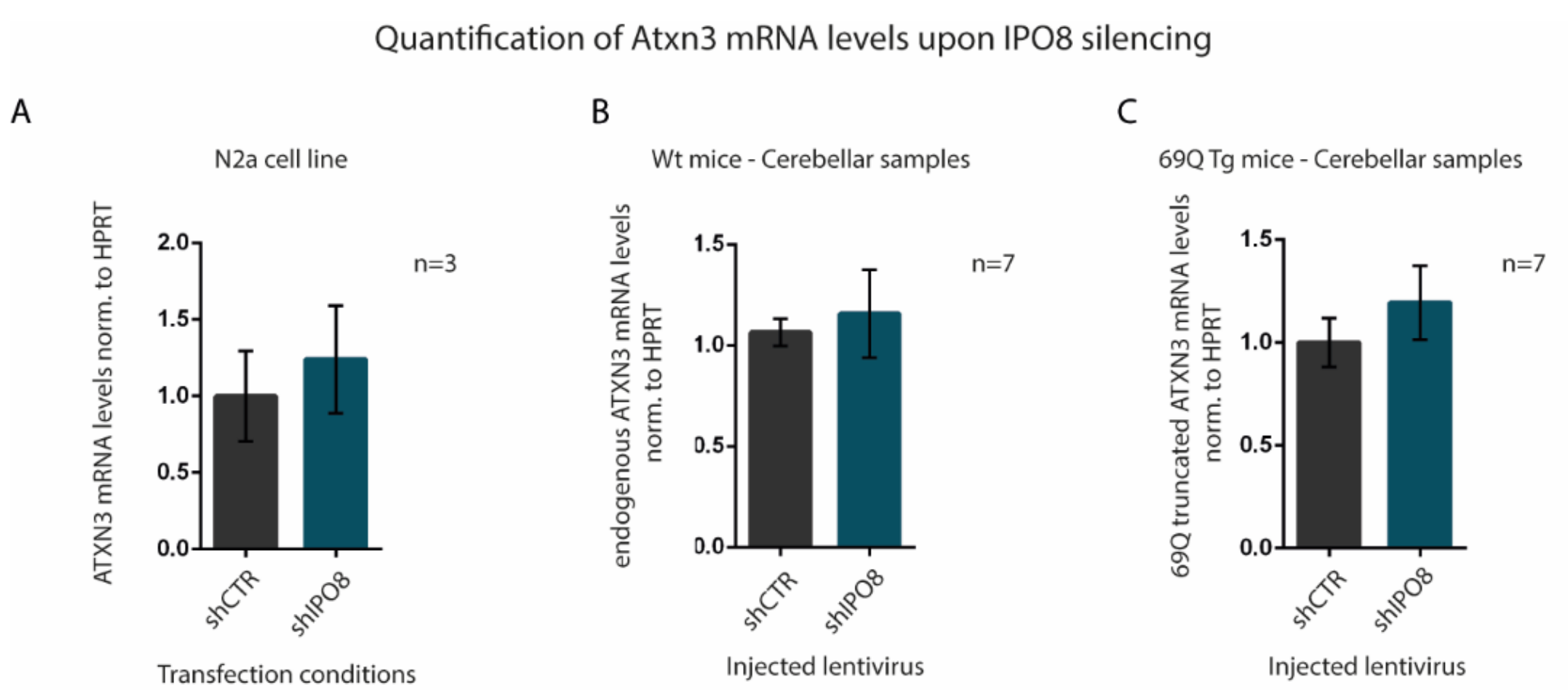


**Fig. S4 – Quantification of *ATXN3* mRNA levels in vitro and in vivo showing no significant differences in *ATXN3* expression upon *Ipo8* silencing conditions:** (**A**) N2a cells transfected with either shCTR or shIPO8 were analyzed by qRT-PCR at 72h post transfection to quantify mouse endogenous ATXN3 mRNA; (**B**) Wt mice stereotaxically injected in the cerebellum (bilaterally in the

DCN and centrally in lobules IX-X) with either shCTR or shIPO8 codifying lentivirus were analyzed by qRT-PCR at 12 weeks post injection to quantify mouse endogenous ATXN3 mRNA; (**C**) 69Q Tg mice stereotaxically injected in the cerebellum (bilaterally in the DCN and centrally in lobules IX-X) with either shCTR or shIPO8 codifying lentivirus were analyzed by qRT-PCR at 12 weeks post injection to quantify human 69Q truncated ATXN3 mRNA. Data is represented as mean +/- SEM.

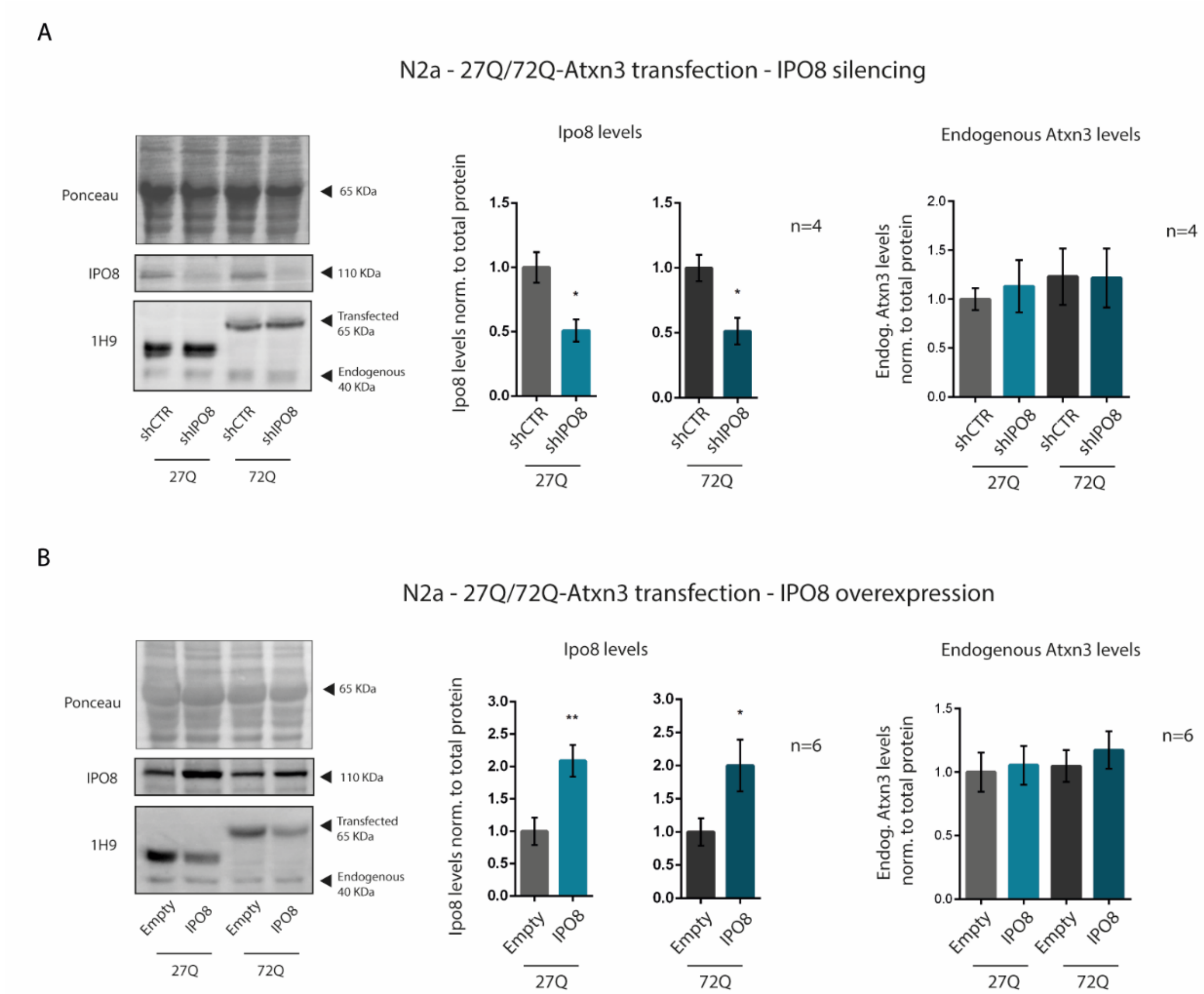


**Fig. S5 – Total protein analysis upon IPO8 modulation:** (A) SDS-PAGE analysis of N2a protein lysates transfected with LV-PGK-27Q or -72Q-Atxn3 and co-transfected with shCTR or shIPO8 for quantification of: Ipo8 protein levels, confirming a significant downregulation of Imp8 that is significant both in cells transfected with 27Q (shIPO8: 0.5095±0.0858 vs: shCTR: 1.000±0.1196, p=0.0158) and 72Q (shIPO8: 0.5135±0.1031 vs. shCTR: 1.000±0.1001, p=0.0148); endogenous Atxn3 levels, showing no statistical difference between conditions. (B) SDS-PAGE analysis of N2a protein lysates transfected with LV-PGK-27Q or 72Q-Atxn3 and co-transfected with Empty or IPO8 for quantification of: Ipo8 protein levels, confirming a significant overexpression of Imp8 that is significant both in cells transfected with 27Q (IPO8: 2.088±0.2463 vs: Empty: 1.000±0.2136, p=0.0085) and 72Q (IPO8: 2.002±0.3899 vs. Empty: 1.000±0.2095, p=0.0470); endogenous Atxn3

levels, showing no statistical difference between conditions. Values are normalized to the respective controls and displayed as mean ± SEM for A to F. *p< 0.05, **<0.01; [Unpaired T-test for Imp8 quantifications; two-way ANOVA with Sidak´s Post Hoc test for endogenous Atxn3 quantifications].

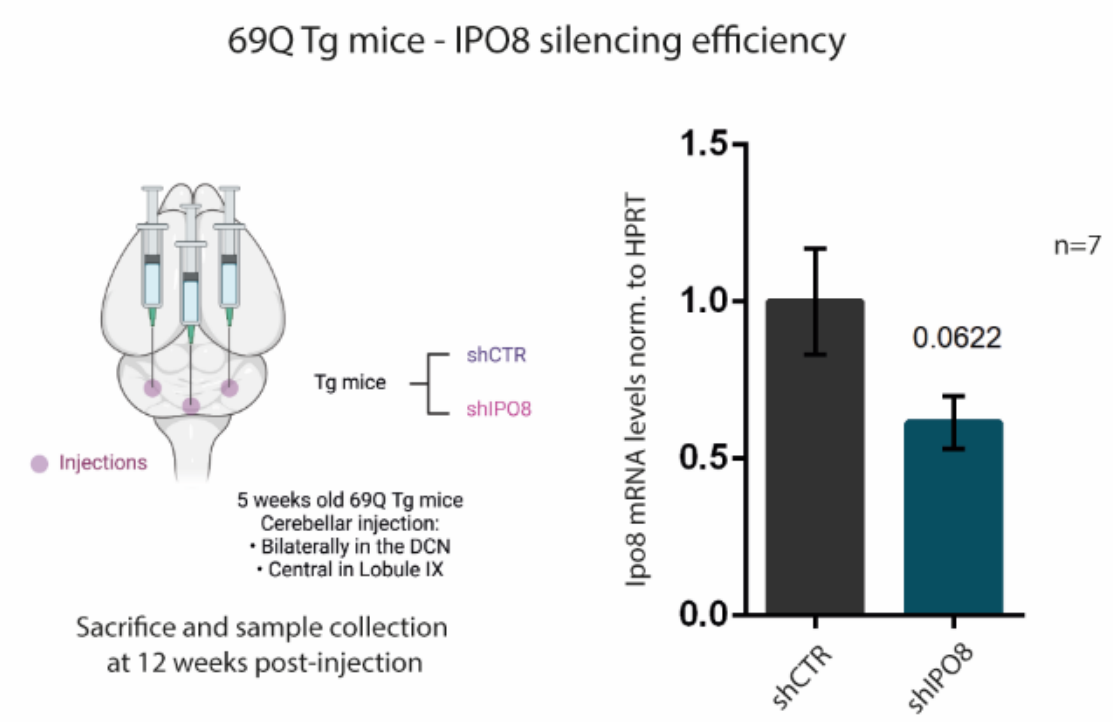


**Fig. S6 – qRT-PCR analysis for confirmation of Ipo8 silencing in cerebellar samples upon delivery of Lentivirus coding for shIPO8 relative to shIPO8 in 69Q Tg animals by cerebellar stereotaxic injection**: a strong tendency for the reduction of Ipo8 expression is observed in cerebella injected with lentivirus coding for shIPO8 relative to animals injected with shCTR encoding lentivirus (shIPO8: 0.6142±0.0836 vs. shCTR: 1.000±0.1697, p=0.0622); Data are represented as mean +/- SEM. Unpaired T-test.

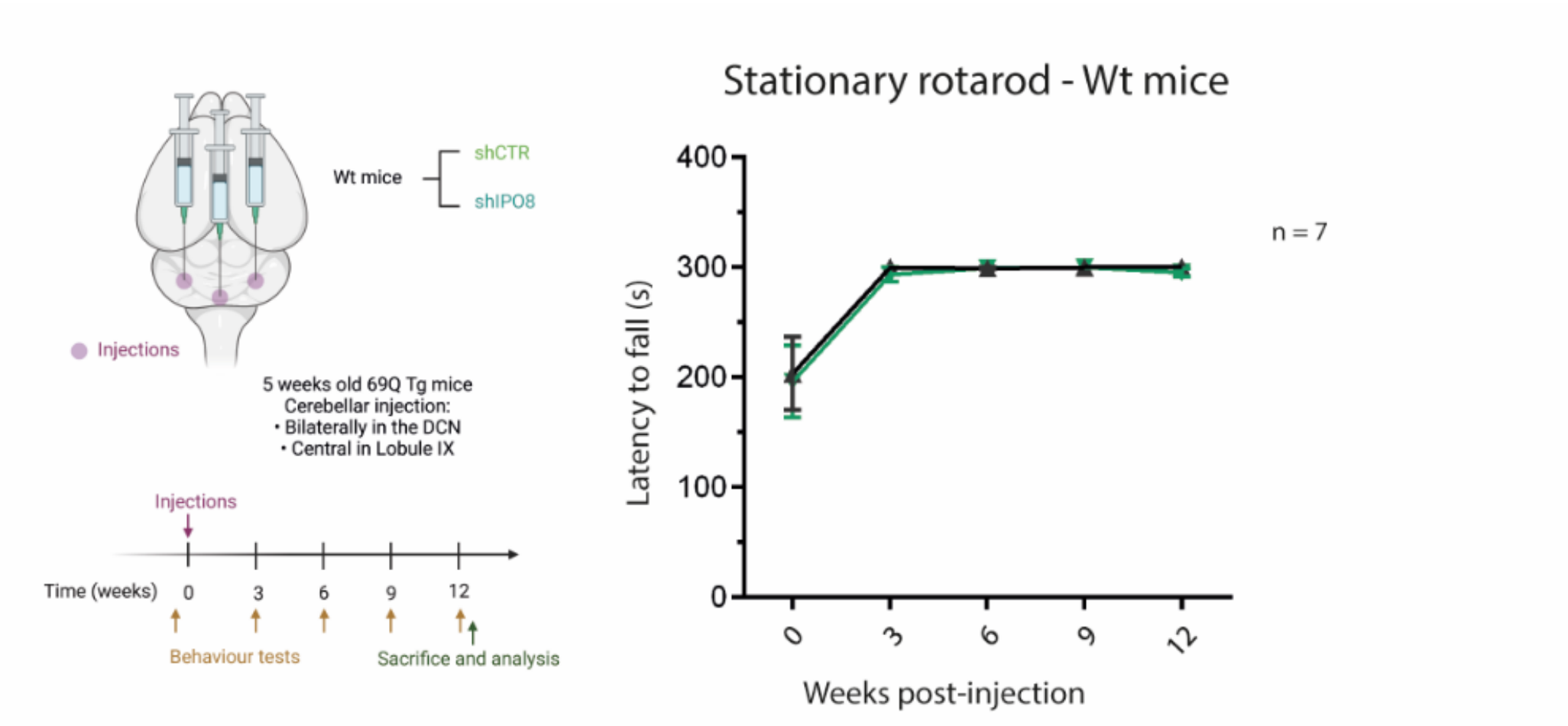


**Fig. S7 – Wild type animals were injected in cerebellum (bilaterally in the DCN and centrally in lobules IX-X) with either shCTR or shIPO8-coding lentivirus.** Behavioral testing was performed before the injections and every 3 weeks until 12 weeks post-injection; the stationary rotarod was performed in all time-points and no difference between the performances was observed, suggesting that no toxic effects are induced by the injection of shIPO8 relative to shCTR coding lentiviruses. Data are represented as mean +/- SEM.

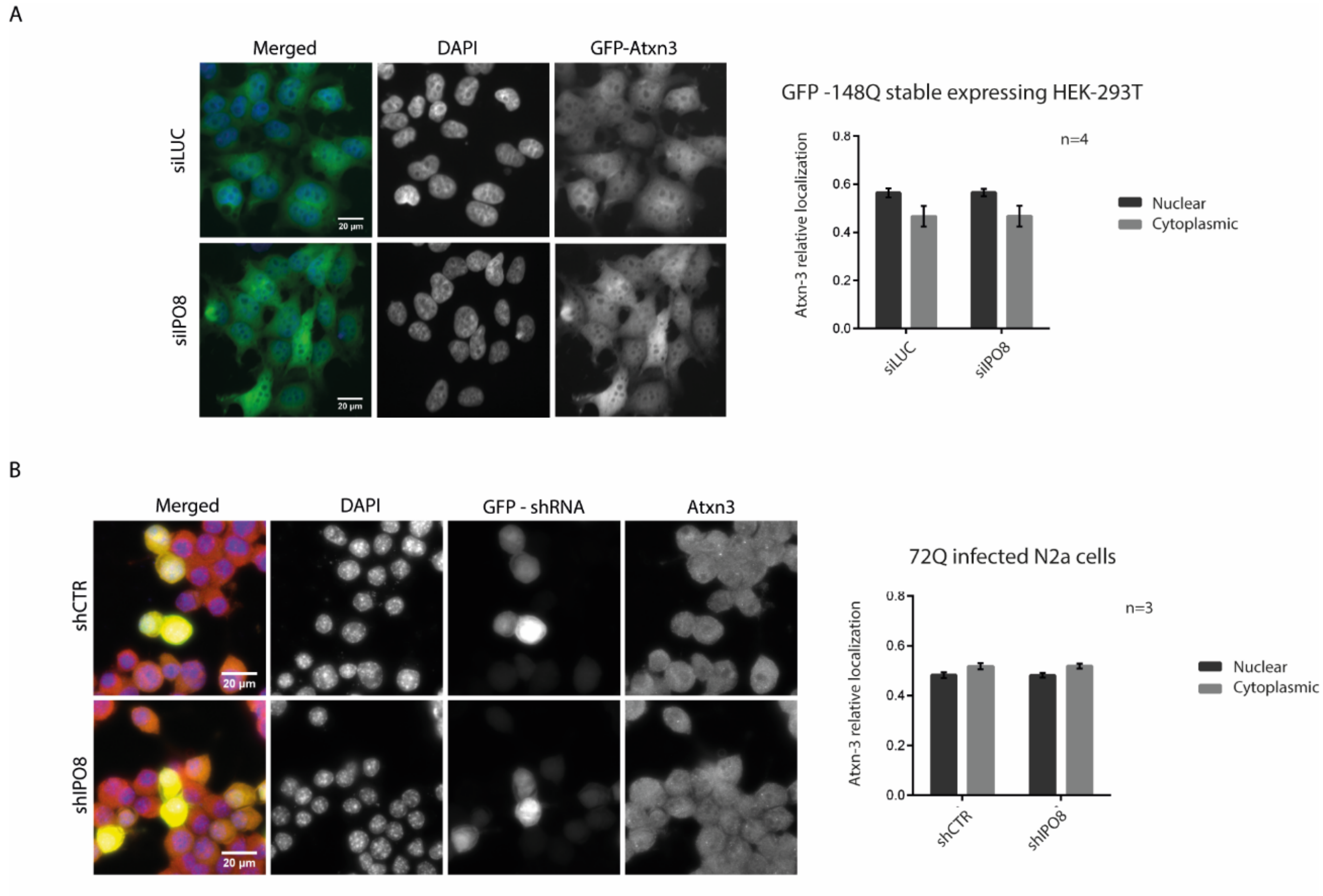


**Fig. S8– *Ipo8* silencing appears no to induce changes in Atxn3 subcellular localization: (A**) Representative microscopy pictures for evaluation of 148Q-Atxn3 subcellular localization in HEK-293T cells silenced for *IPO8* using siRNA and respective statistical analysis showing no differences in the nuclear or cytoplasmic Atxn3 mean intensities; Scale bars are corresponding to 20 µm. (**B**) Representative microscopy pictures for evaluation of 72Q-Atxn3 subcellular localization in transduced N2a cells silenced for *Ipo8* using shRNA and respective statistical analysis showing no differences in the nuclear or cytoplasmic Atxn3 mean intensities. Scale bars are corresponding to 20 µm. Values are displayed as mean ± SEM - two-way ANOVA with Sidak´s Post Hoc test for A and B.

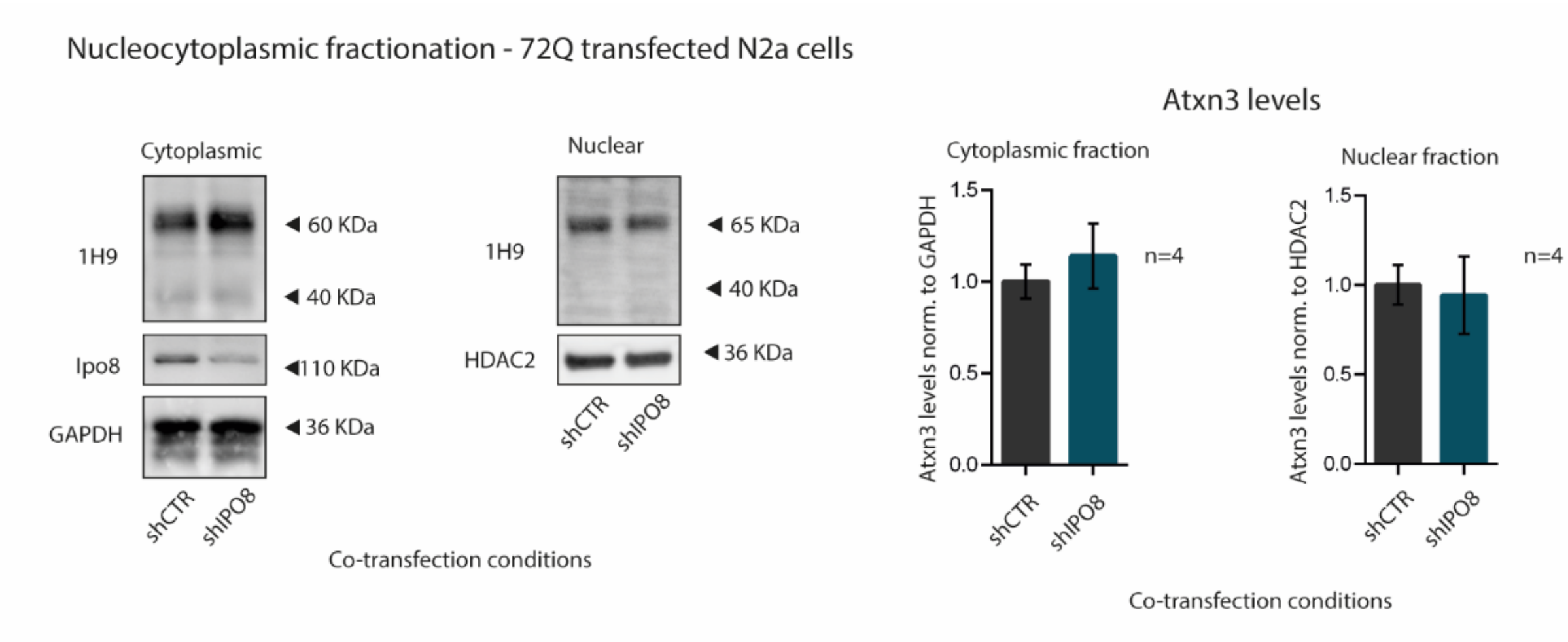


**Fig. S9 - Immunoblot for analysis of Atxn3 subcellular localization in 72Q transfected N2a cells -** cells co-transfected with shIPO8 or shCTR, show no significant differences neither on the cytoplasmic nor the nuclear levels of Atxn3. Values are normalized to the respective controls (GAPDH or HDAC2, respectively) and displayed as mean ± SEM; Unpaired T-tests.

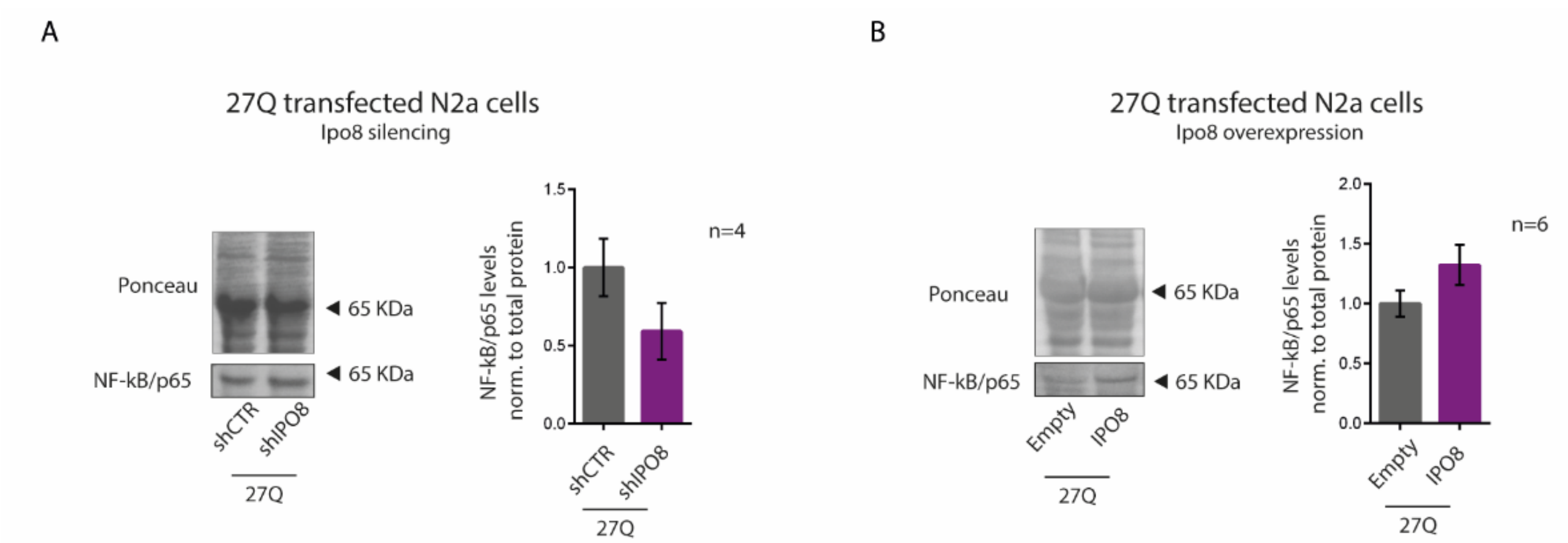


**Fig. S10 - Immunoblot for detection of total protein levels of NF-kB/p65 in 27Q transfected N2a in:** (A) cells show no significant differences on NF-kB/p65 levels upon co-transfected with shIPO8 when compared to the shCTR control condition; (B) cells co-transfected with IPO8 or Empty constructs, showing no significant differences on NF-kB/p65 levels in cells co-transfected with

shIPO8. Values are normalized to the respective controls and displayed as mean ± SEM; Unpaired T-tests.

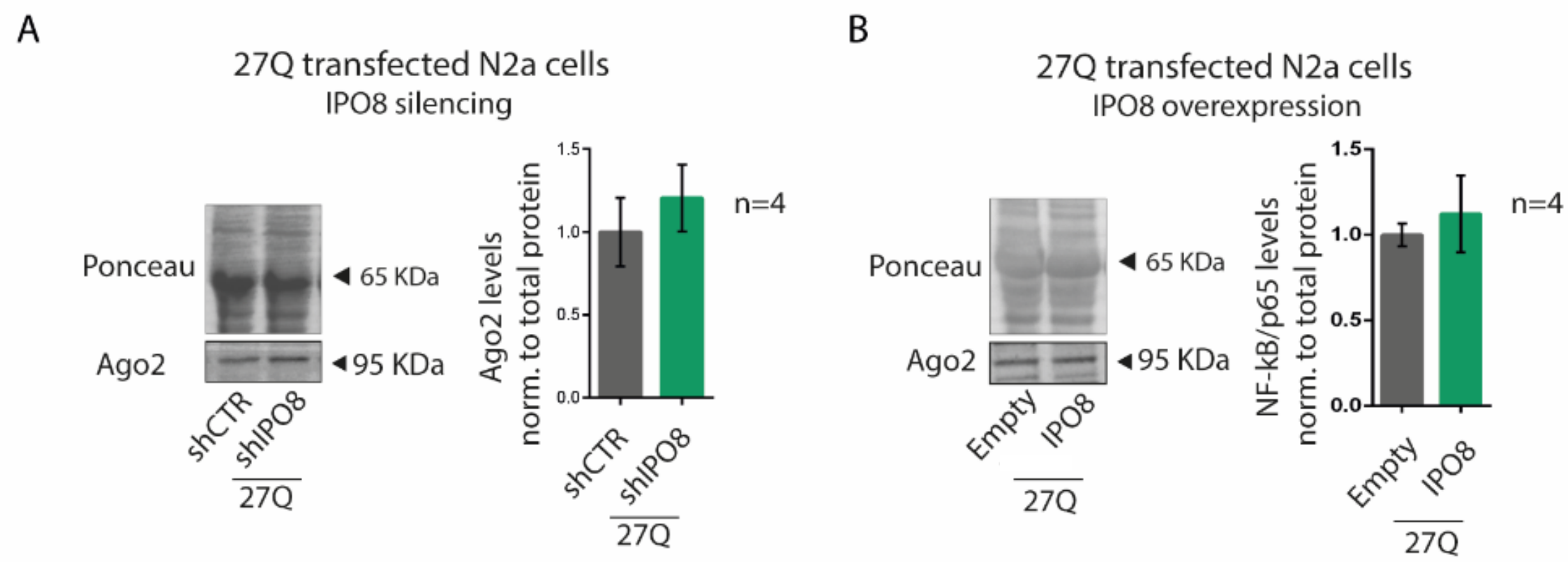


**Fig. S11 - Immunoblot for detection of total protein levels of Ago2 in 27Q transfected N2a in:** (**A**) cells co-transfected with shIPO8 or shCTR, showing no significant differences neither on Ago2 levels in cells co-transfected with shIPO8 when compared to the control condition; (**B**) cells co-transfected with IPO8 or Empty constructs, showing no significant differences neither on Ago2 levels in cells co-transfected with shIPO8 when compared to the control condition. Values are normalized to the respective controls and displayed as mean ± SEM; Unpaired T-tests.

**Table S2 - List of oligonucleotide sequences used for oligo-based cloning of constructs encoding for shRNAs targeting mouse Ipo8.**

| shRNA | Oligonucleotide sequence | |
|---|---|---|
| shCTR | 5’ | GATCCCCCAACAAGATGAAGAGCACCAATTCAAGAGATTGGTGCTCTTCATCTTGTTGTTTTTA |
| | 3’ | AGCTTAAAAACAACAAGATGAAGAGCACCAATCTCTTGAATTGGTGCTCTTCATCTTGTTGGGG |
| shIPO8_1 | 5’ | GATCCCCCCACCAGGAGAAGTGATATTTTTCAAGAGAAAATATCACTTCTCCTGGTGGTTTTTA |
| | 3’ | AGCTTAAAAACCACCAGGAGAAGTGATATTTTCTCTTGAAAAATATCACTTCTCCTGGTGGGGG |
| shIPO8_2 | 5’ | GATCCCCCTACTGTAACGTACGAAATTTTTCAAGAGAAAATTTCGTACGTTACAGTAGTTTTTA |
| | 3’ | AGCTTAAAAACTACTGTAACGTACGAAATTTTCTCTTGAAAAATTTCGTACGTTACAGTAGGGG |

**Mouse shRNA validation**

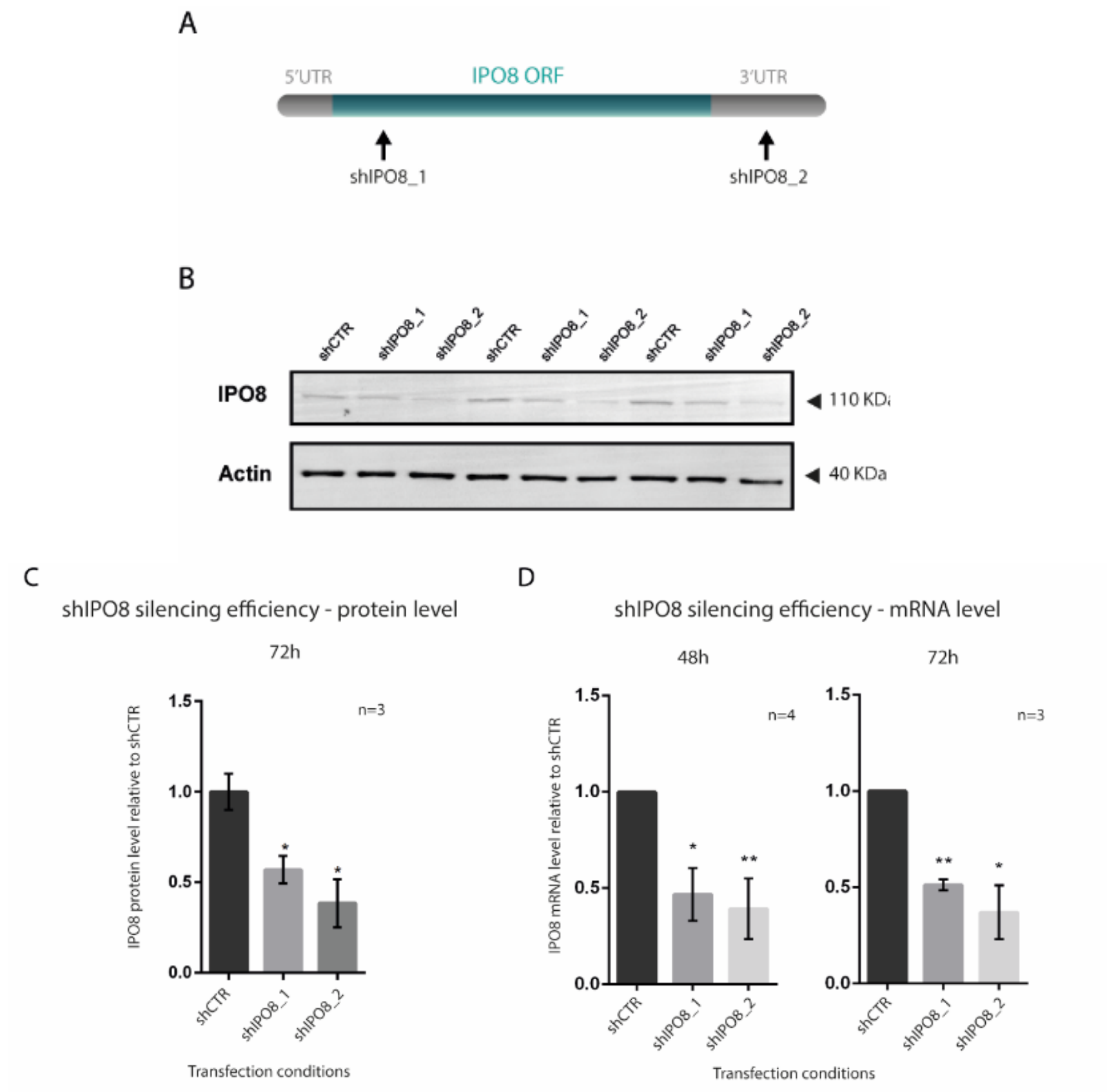


**Fig. S12 – Mouse *Ipo8* shRNA validation in N2a cells:** (**A**) Illustration of the targeting regions of shIPO8_1 and shIPO8_2; (**B**) SDS-PAGE analysis for quantification of Imp8 protein levels in N2a cells transfected with 1µg of shCTR, shIPO8_1 or shIPO8_2 collected 72h post-transfection; (**C**) Both shRNAs targeting *Ipo8* significantly reduced the protein levels of Imp8. However, shIPO8_2 showed a more robust and visible decrease in Imp8 protein (shIPO8_1: 0.5697±0.0760, *p*=0.0270; shIPO8_2: 0.3842±0.1337, *p*=0.0212; shCTR: 1.000±0.1007) incubation periods; (**D**) N2a cells were transfected with 1µg of shCTR, shIPO8_1 or shIPO8_2 and collected 48h or 72h post-transfection to be analyzed by real time qRT-PCR for *Ipo8* mRNA expression. Results show that both *Ipo8* shRNAs can significantly reduce *Ipo8* mRNA levels and no evident difference was found between 48h (shIPO8_1: 0.4679±0.1371, *p*=0.0303; shIPO8_2: 0.3924±0.0789, *p*=0.0045) or 72h (shIPO8_1: 0.5127±0.0282, *p*=0.0033; shIPO8_2: 0.3704±0.1406, *p*=0.0464) incubation periods. Data are represented as mean +/- SEM.

**Illustration of the expression constructs used**

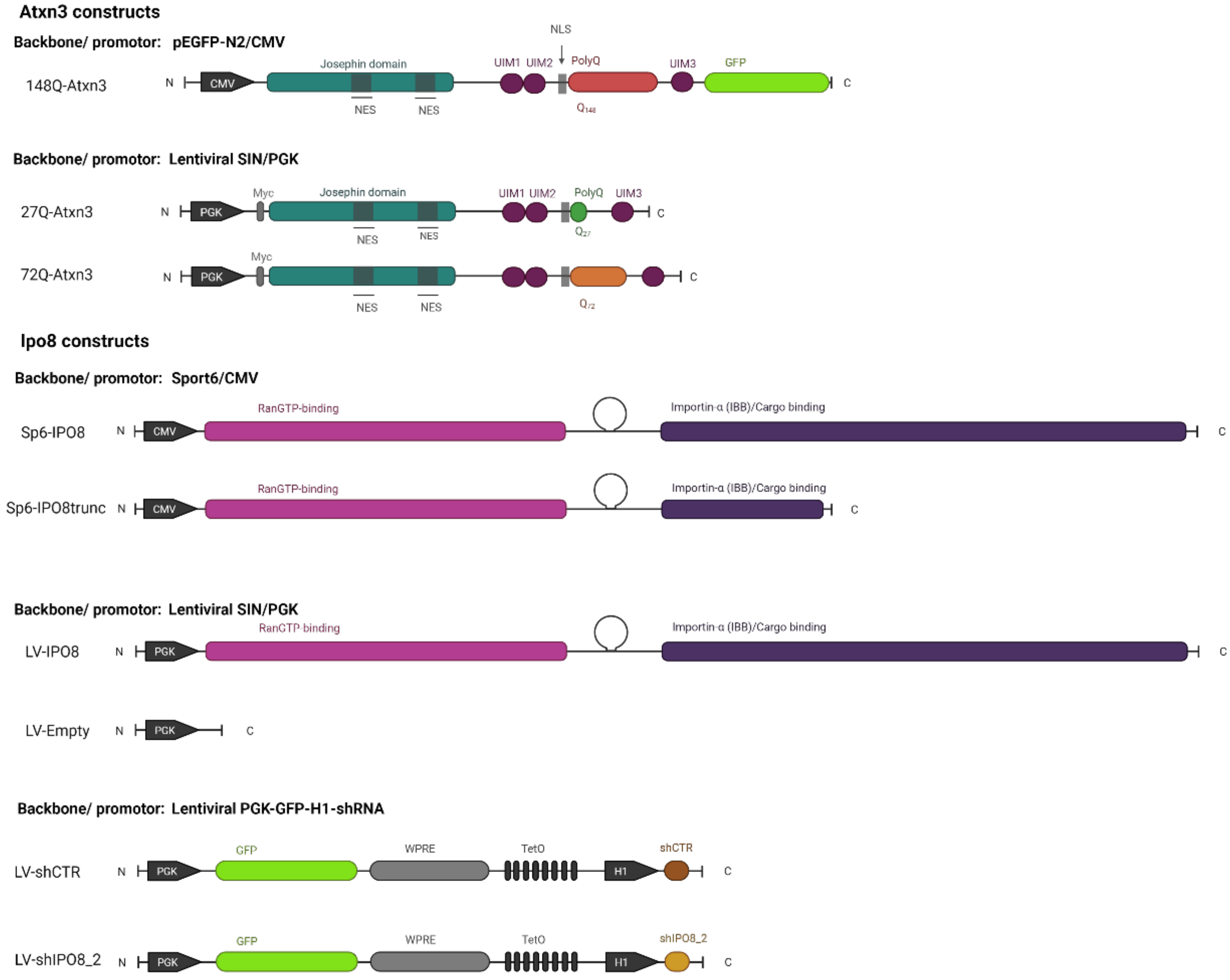


**Fig. S13 – Illustration of the expression constructs used for cell transfection and viral productions.**

Optimization of the fractionation protocol

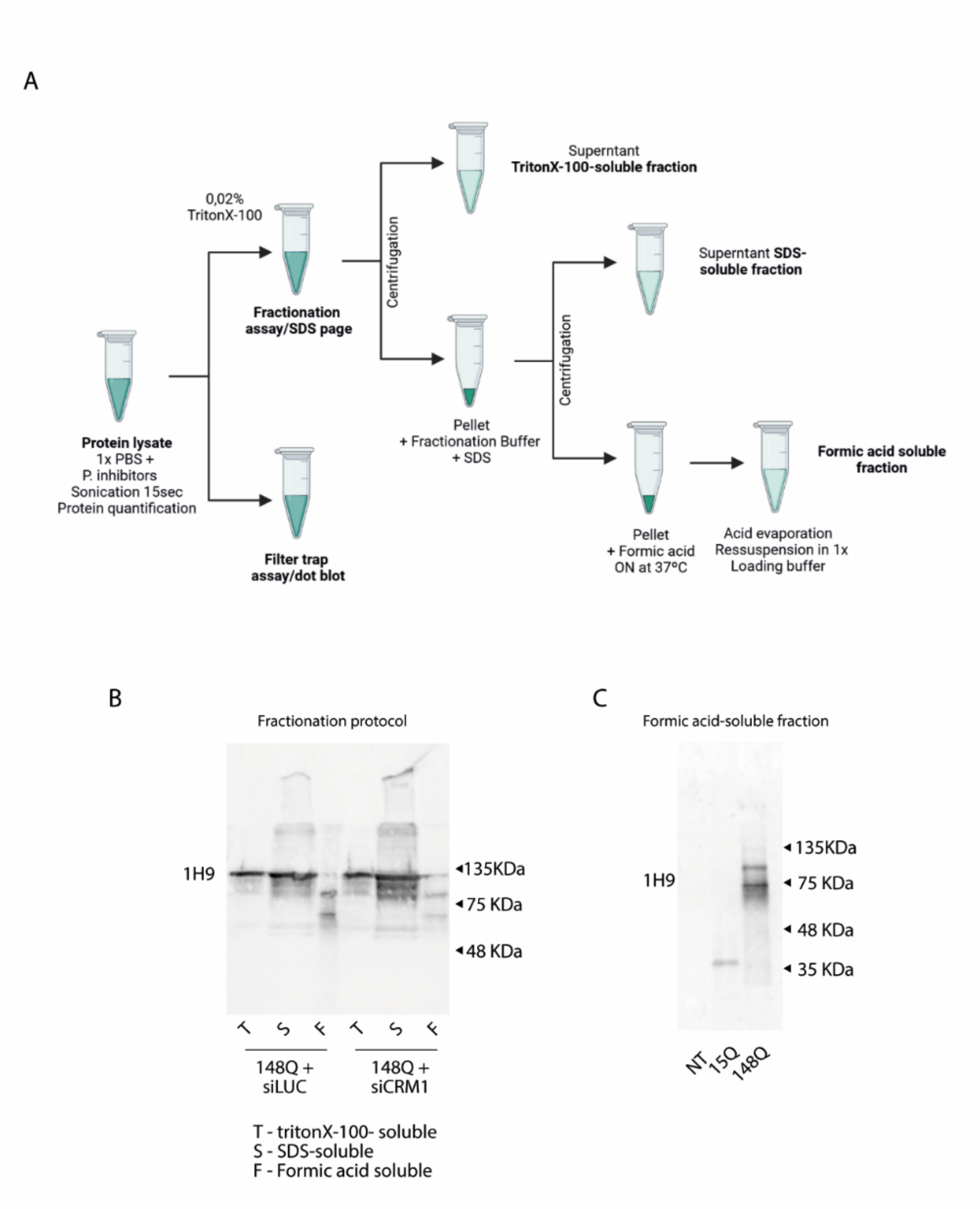


**Fig. S14 – Optimization and analysis of the fractionation protocol for Atn3 aggregation analysis.** (**A**) Adaptation of fractionation protocol from Koch et al. to start form samples collected in 1x PBS and lysed with ultrasonication, so that the same samples can be used to perform filter trap and fractionation protocol parallelly. (**B**) SDS-PAGE membrane representing the 3 protein fractions collected in the fractionation protocol: Triton X-100 soluble, SDS-soluble, and formic acid-soluble fractions. 148Q expressing HEK-293T cells co-transfected with siLUC vs. siCRM1 was used as proof of concept. (**C**) SDS-PAGE membrane showing the formic acid-soluble fraction collected form non-

transfected HEK-293T cells and cells expressing 15Q or 148Q-Atxn3. No formic acid-soluble Atxn3 was visible in non-transfected cells, a week signal was observed in 15Q expressing cells and as expected, a strong signal was observed in higher molecular weights for cells expressing 148Q-Atxn3.

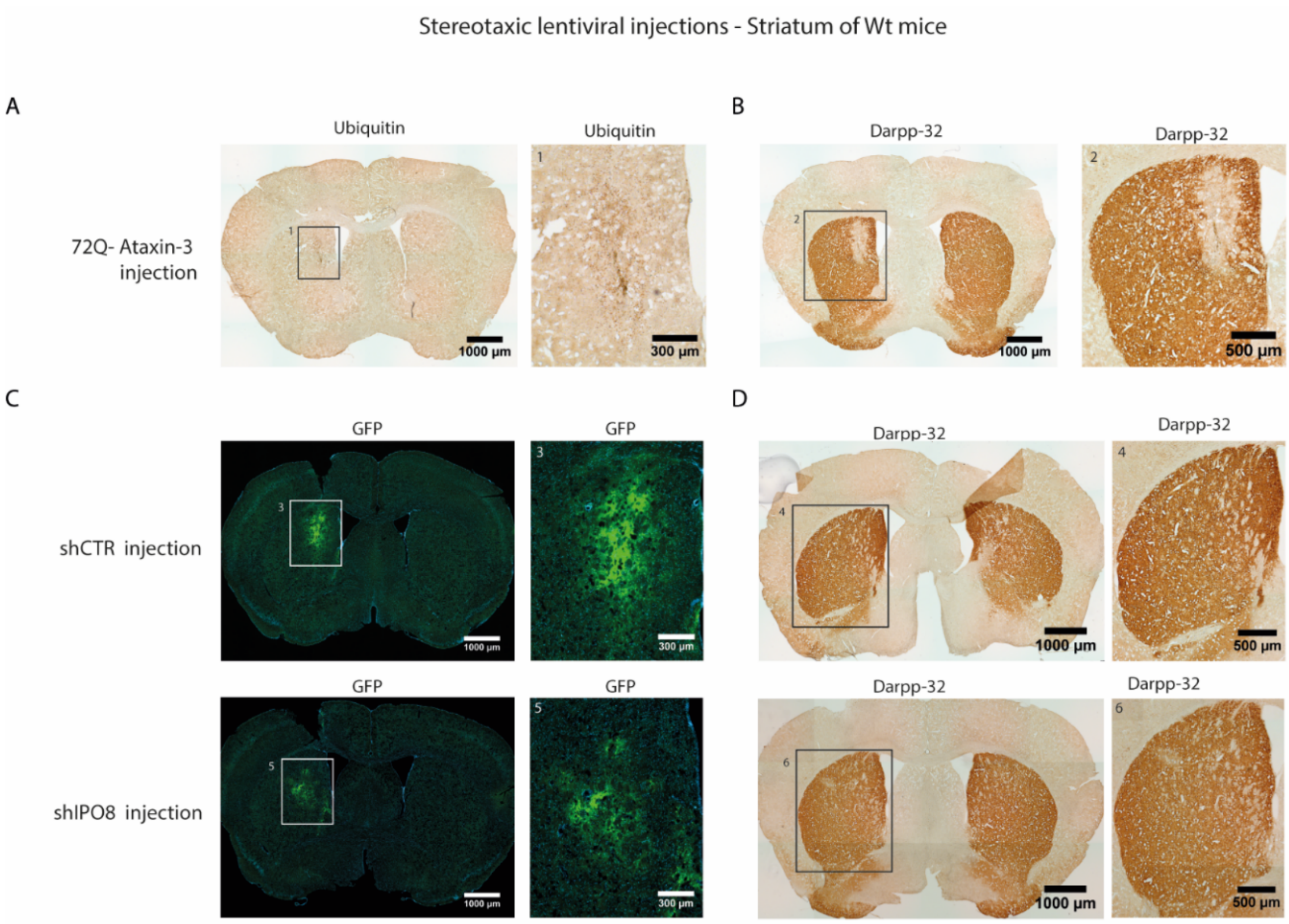


**Fig. S15 – Striatal stereotaxic injection for confirmation of lentiviral transgene expression and evaluation of toxicity:** (**A**) injection of lentivirus coding for 72Q-Atxn3 in the left striatum of a Wt mouse – 72Q-Atxn3 expression close to the injection site leads to the formation of Atxn3 protein inclusions that are ubiquitin positive, as previously reported;[3,4.] Scale bar of 1000µm and respective zoom with scale bar of 300 µm. (**B**) injection of lentivirus coding for 72Q-Atxn3 in the left striatum of a Wt mouse – 72Q-Atxn3 expression close to the injection site leads to neuronal dysfunction observed by the reduced expression of the DARPP-32 marker, as previously reported;[3,4] Scale bar of 1000µm and respective zoom with scale bar of 500 µm(**C**) injection of lentivirus coding for shCTR or shIPO8 in the left striatum of two individual Wt mice –

shCTR and shIPO8 can be assumed by the co-expression of GFP close to the injection site, which sequence is coded in the same lentiviral plasmid used for the lentiviral production as the shRNAs; Scale bars of 1000μm and respective zooms with scale bars of 300 μm (**D**) injection of lentivirus coding for shCTR or shIPO8 in the left striatum of two individual Wt mice – neither shCTR nor shIPO8 expression leads to neuronal dysfunction close to the injection site since no reduction on the expression of the DARPP-32 marker was observed. Scale bars of 1000μm and respective zooms with scale bars of 500 μm.

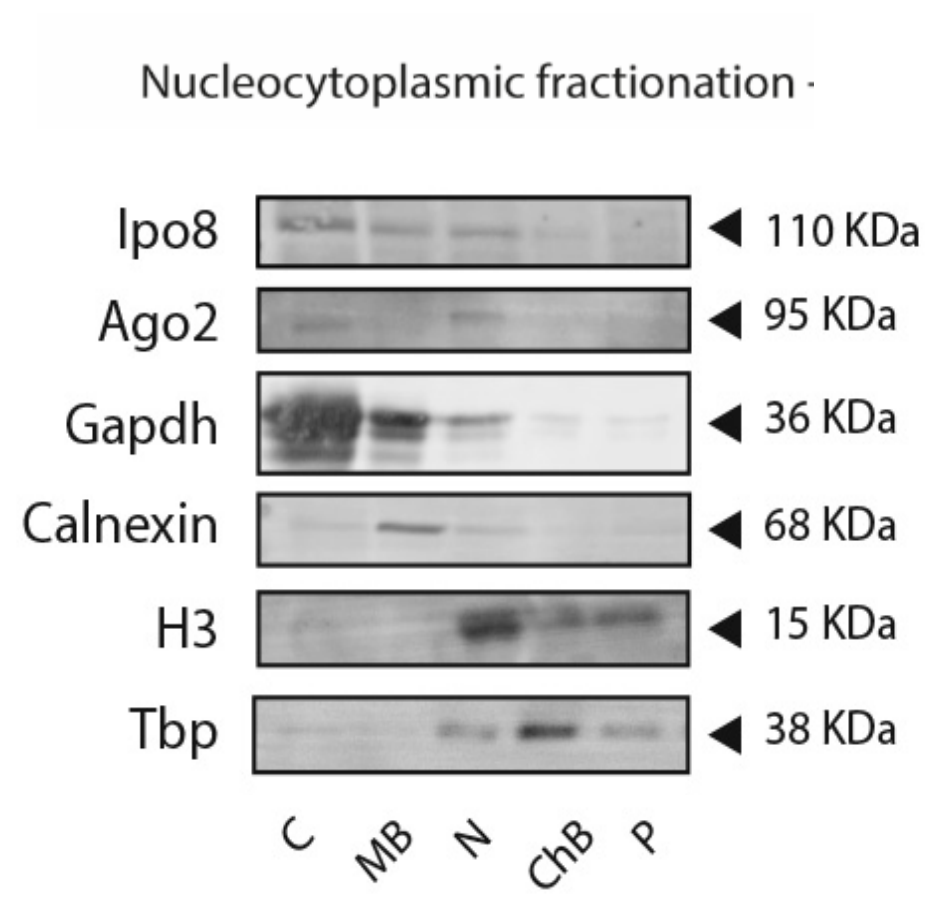


**Fig. S16 - Immunoblot for detection of protein markers for the different cellular fractions obtained when using the Subcellular Protein Fractionation Kit (see materials and methods) in cerebellar samples of wild type mice:** C- cytoplasmic soluble fraction; MB – membrane bound fraction; N – Nuclear soluble fraction; ChB – Chromatin bound fraction; P – pellet fraction (remaining cytoskeletal proteins).

**Table S3 -** List of Antibodies used in western blot protocols, respective dilutions, brand, and reference ID.

| Antibody | Host | Dilutions | Brand | Reference |
|---|---|---|---|---|
| CRM1 | Rabbit | 1:500 in 5% milk | Santa Cruz Biotechnology | sc-5595 |
| IPO13 | Mouse | 1:500 in 5% milk | Santa Cruz Biotechnology | sc-271218 |
| KPNA3 | Rabbit | 1:1000 in 5% milk | Abcam | AB137446 |
| Importin-8 | Mouse | 1:500 in 5% milk | Santa Cruz Biotechnology | sc-398854 |
| Ataxin-3, clone 1H9 | Mouse | 1:2000 in 5% milk | Merck Millipore | MAB5360 |
| Beta-actin, clone AC-74 | Mouse | 1:5000 in 5% milk | Sigma- Aldrich | A5316 |
| GAPDH | Mouse | 1:2000 in 5% milk | Sigma- Aldrich | MAB374 |
| HDAC2 (D6S5P) | Rabbit | 1:1000 in 5% BSA | Cell Signaling | #57156 |
| Nf-kB/p65, F-6 | Mouse | 1:1000 in 5% BSA | Santa Cruz Biotechnology | sc-8008 |
| phospho Nf-kB/p65 | Rabbit | 1:1000 in 5% BSA | Invitrogen | MA5-15160 |
| Anti-Mouse IgG, HRP | Sheep | 1:10000 in 5% Milk | GE Healthcare | NA931 |
| Anti-Rabbit IgG, HRP | Donkey | 1:10000 in 5% Milk | GE Healthcare | NA934 |
| anti-Mouse IgG, IRDye 680RD | Donkey | 1:10000 in 5% Milk | LI-COR | 926–68072 |
| anti-Mouse IgG, AP | Goat | 1:10000 in 5% milk | Invitrogen | #31328 |
| anti-Rabbit IgG, AP | Goat | 1:10000 in 5% milk | Invitrogen | #31340 |

**Table S4 -** List of custom designed oligonucleotides sequences used in quantitative RT-PCR analysis

| Primer | Forward | Reverse |
|---|---|---|
| *Human Crm1* | CCTTGGAAACATTGCTCAGA | GGCTTACACTCACACCAGCA |
| *Human Tnpo1* | TAAGACCCTGGTGCACCTCT | CCAATTTTCATCGCCAACTT |
| *Human Ipo13* | AGCTGGTCCACATCTTTGCT | CATCCAATCGTTCACACAGG |
| *Human Pdh* | GGTTTCCCATTCAAGACCTG | TGGTTTCCATGTCCATTGGT |
| *Human B-Actin* | TCCCTGGAGAAGAGCTACGA | AGGAAGGAAGGCTGGAAGAG |
| *Human Ywhaz* | TCCAGGGACAGAGTCTCAGC | TCCATGACTGGATGTTCTGTG |

**Table S5** - List oligonucleotide sequences used in PCR analysis for 69Q transgenic mouse genotyping.

| Primer | Forward | Reverse | Amp. size |
|---|---|---|---|
| *Human ATXN3* | ATGTACCCATACGATGTTCC | CTAGCGAGGGAATGAAGAAT | 450 bp |
| *Mouse B-Actin* | GGAGACGGGGTCACCCACAC | AGCCTCAGGGCATCGGAACC | 300 bp |

## Supplementary Data – Original Blots

*Figure 1A*

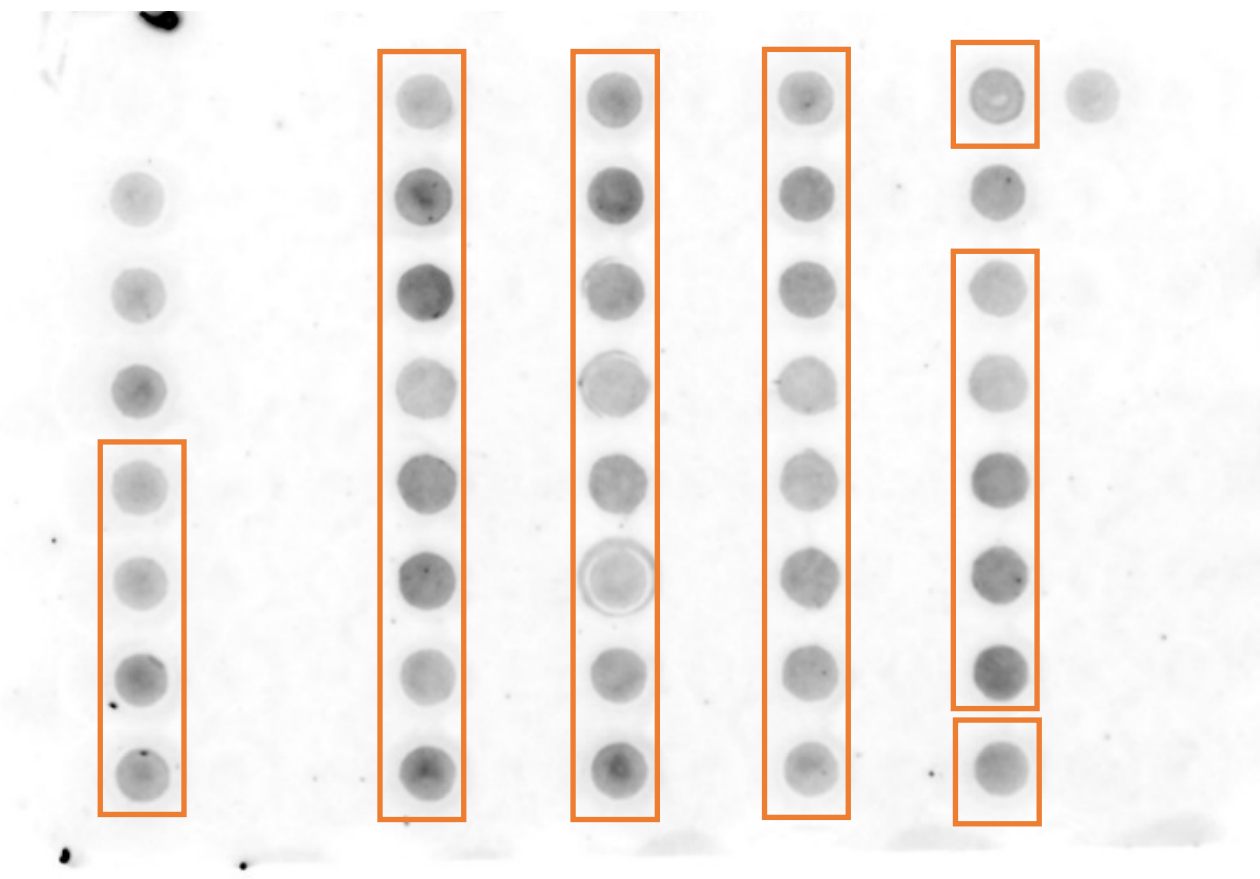

*Figure 1B*

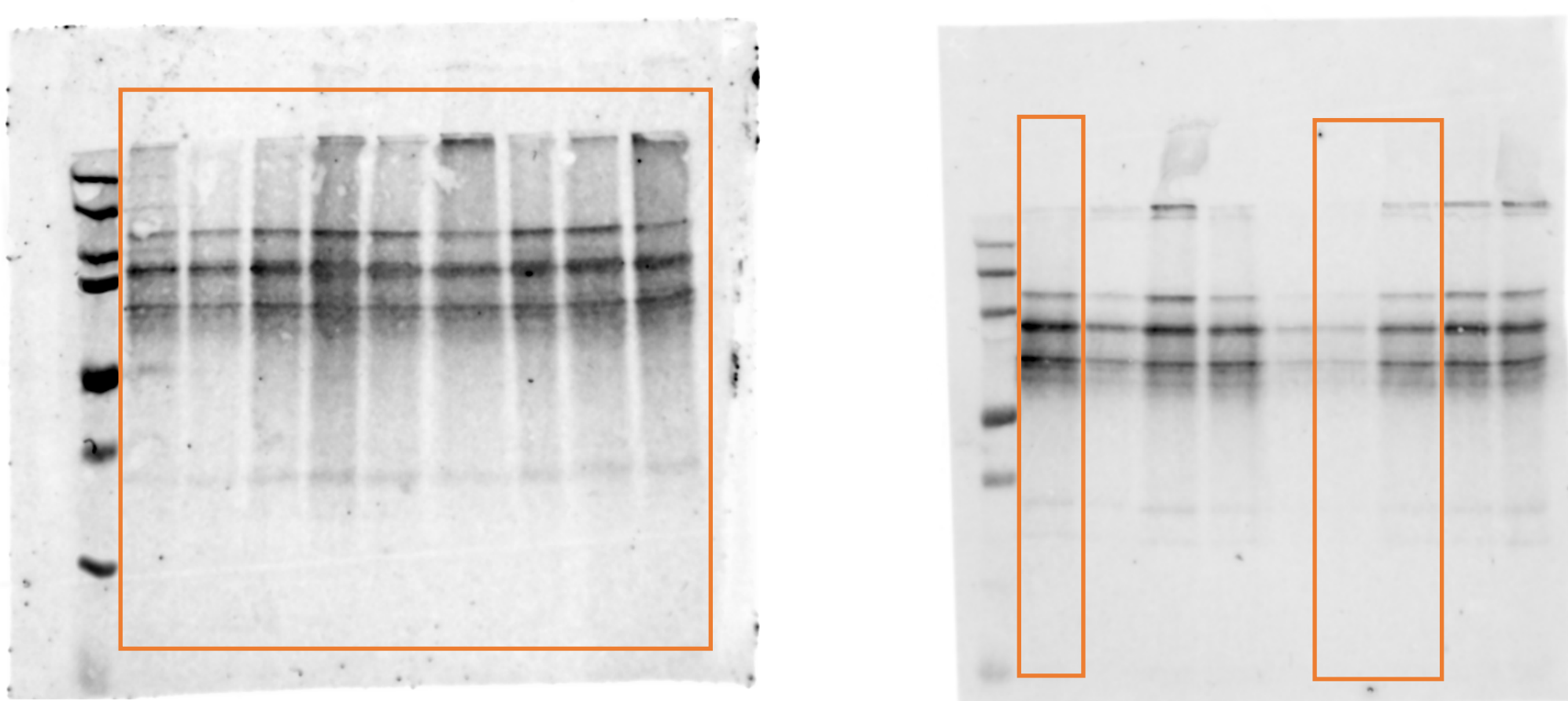

*Figure 1C*

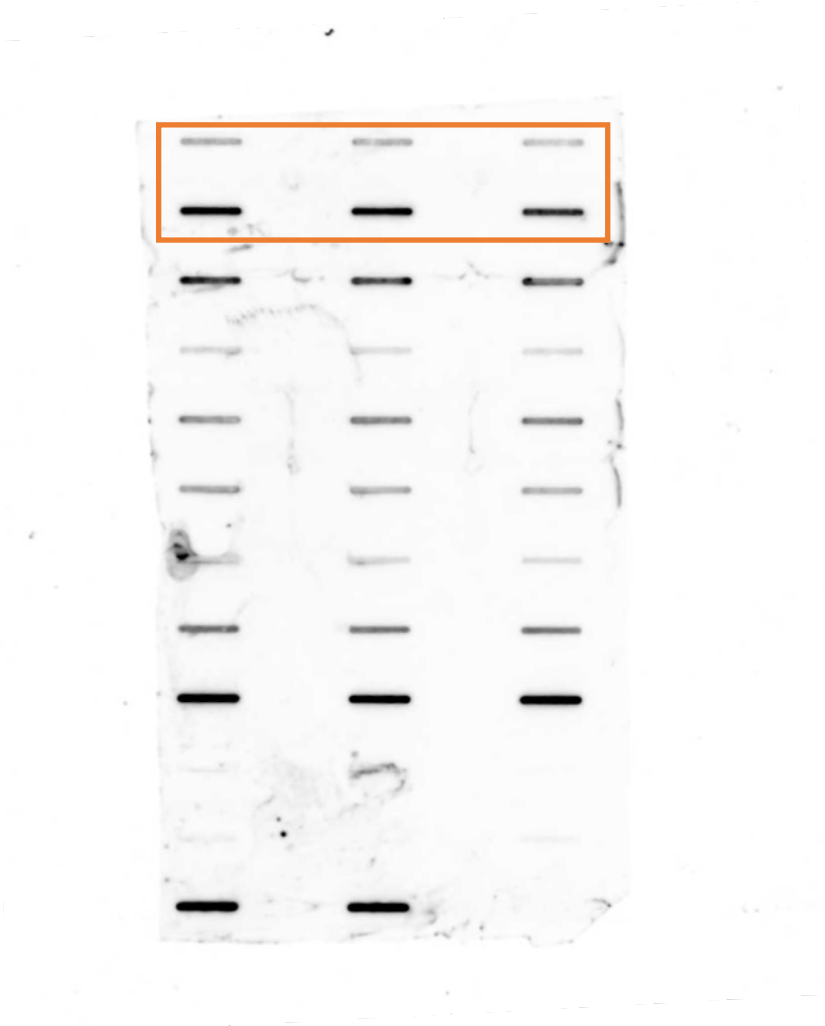

*Figure 1D*

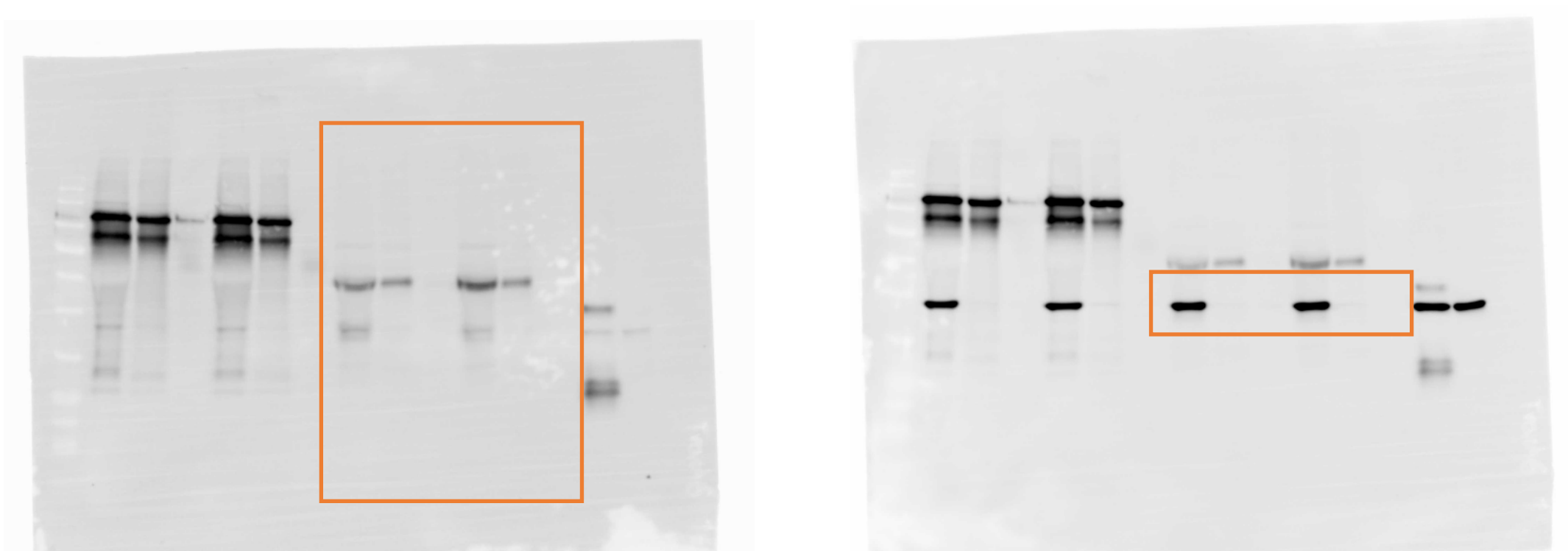

*Figure 1E*

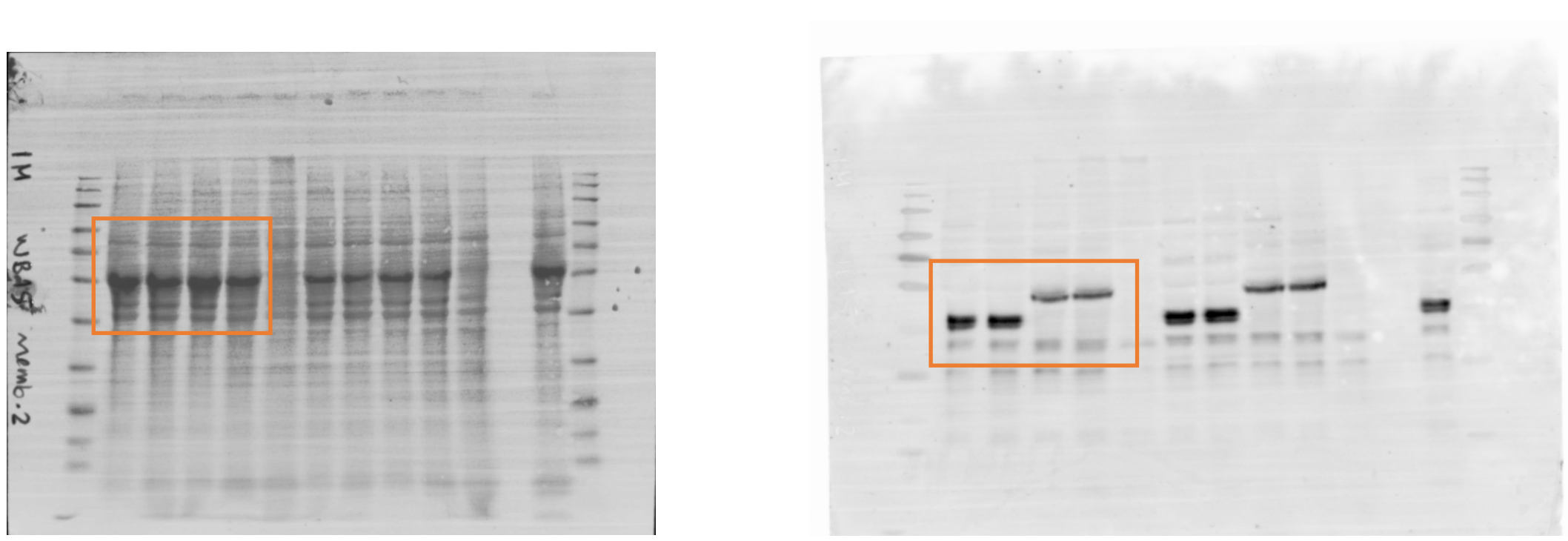

*Figure 1F*

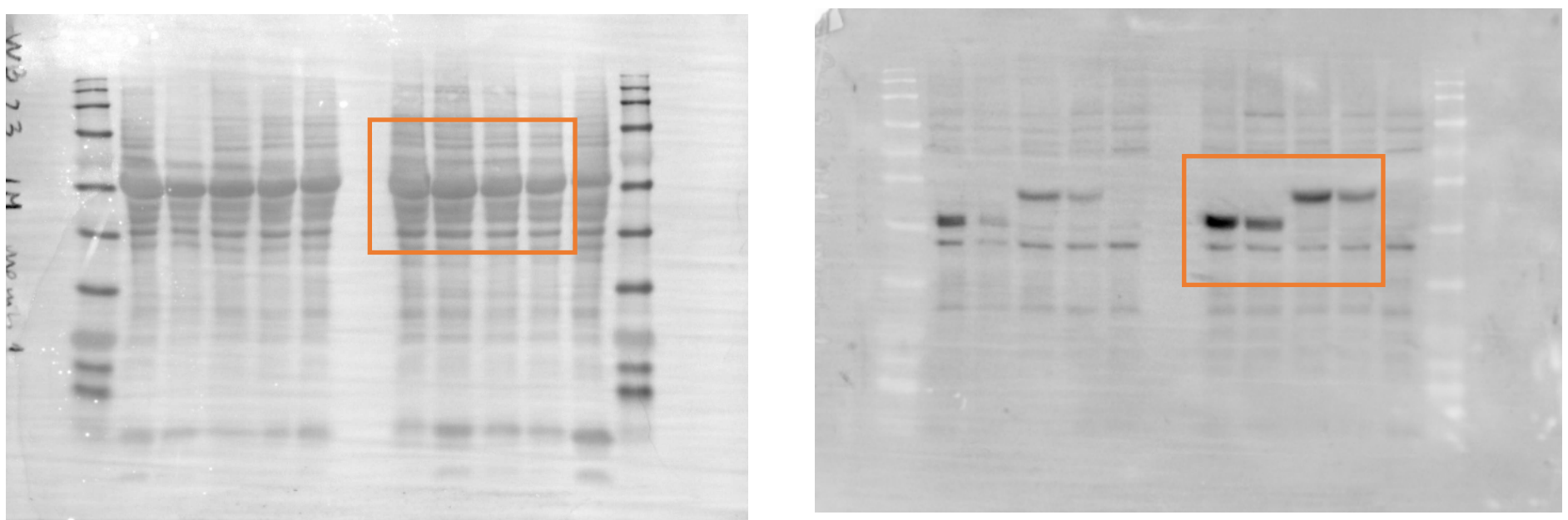

*Figure 2A*

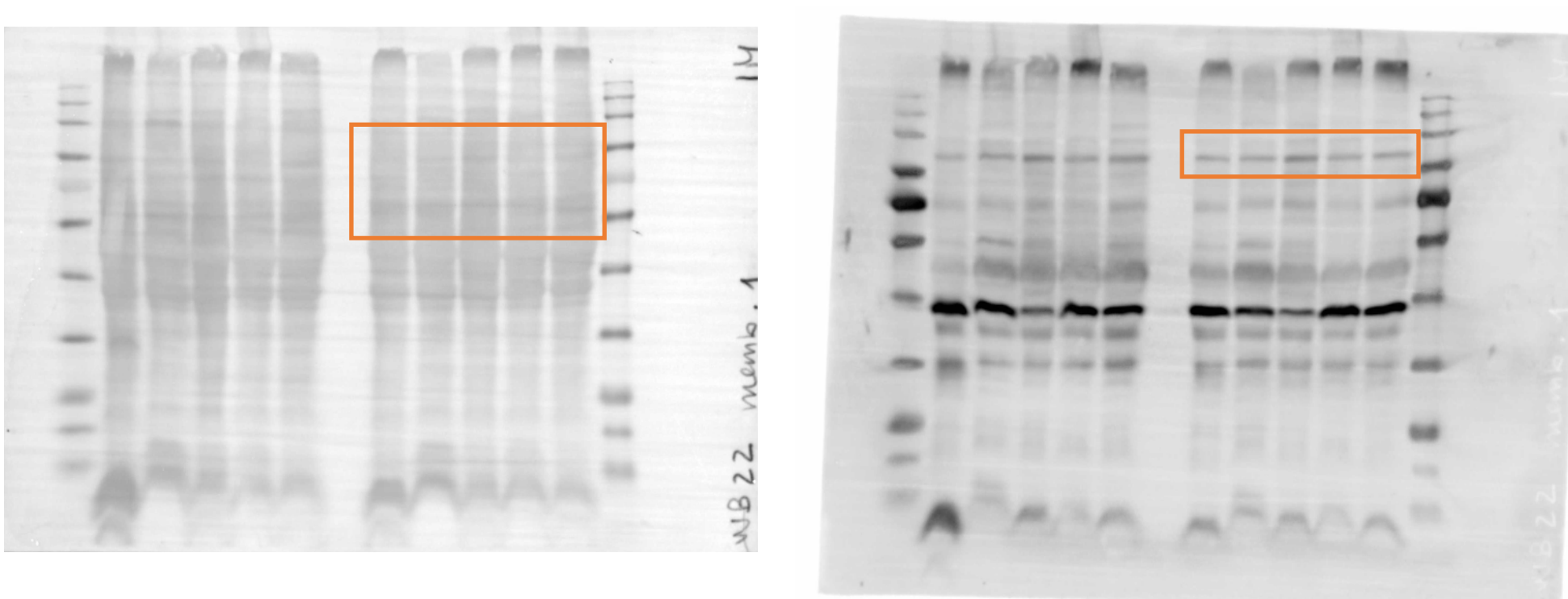

*Figure 2B*

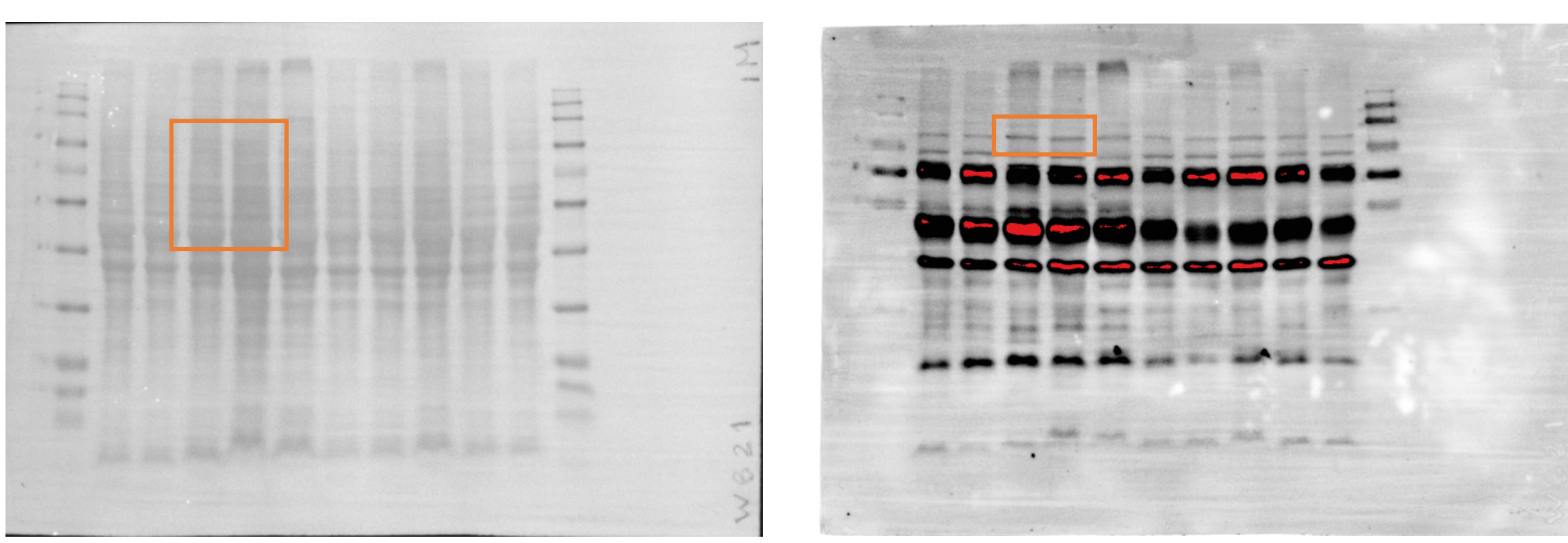

*Figure 2D*

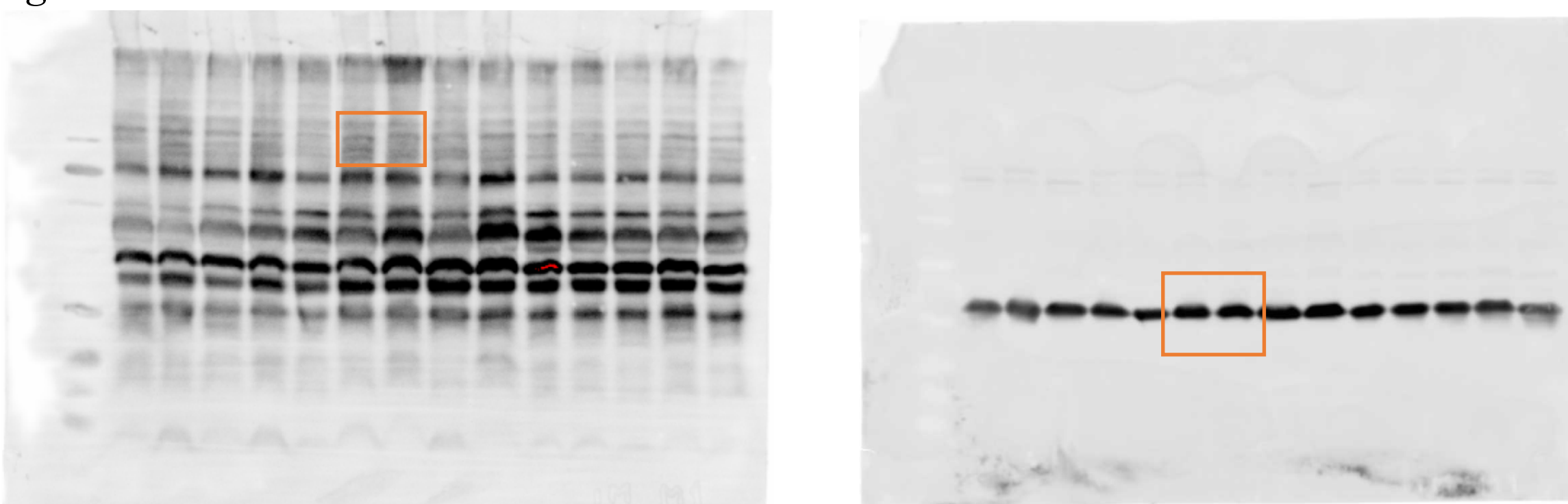

*Figure 5A*

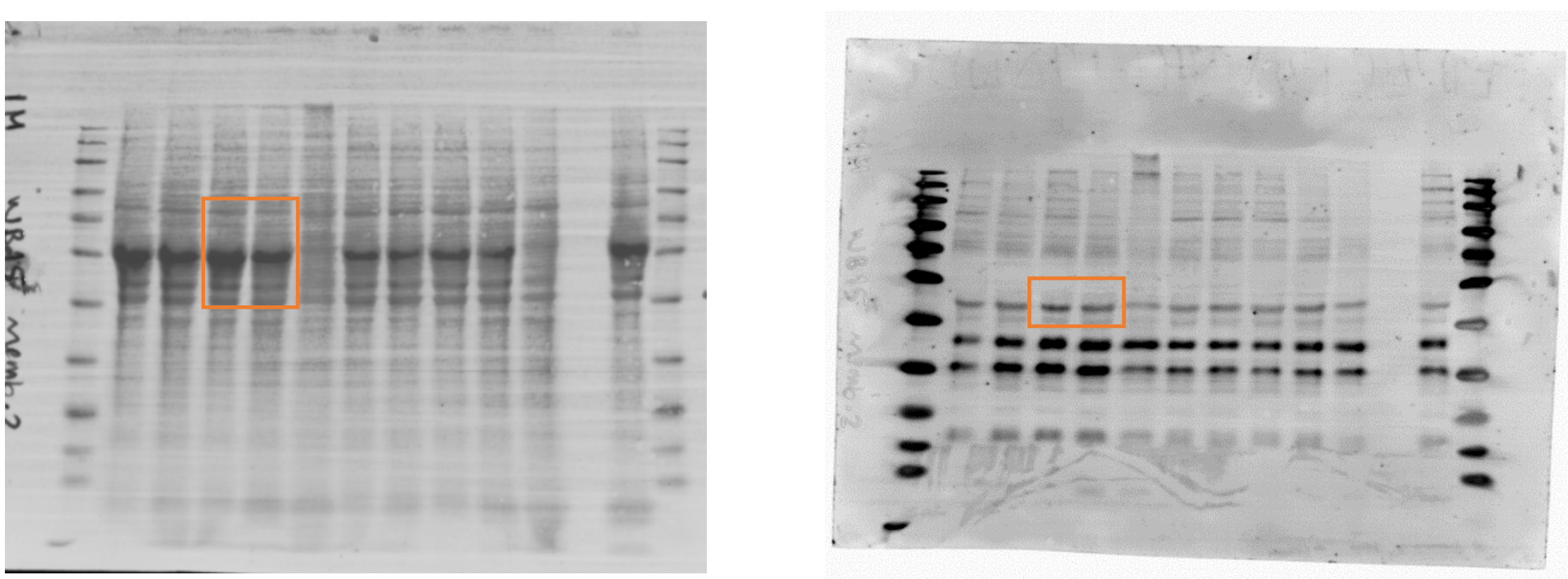

*Figure 5B*

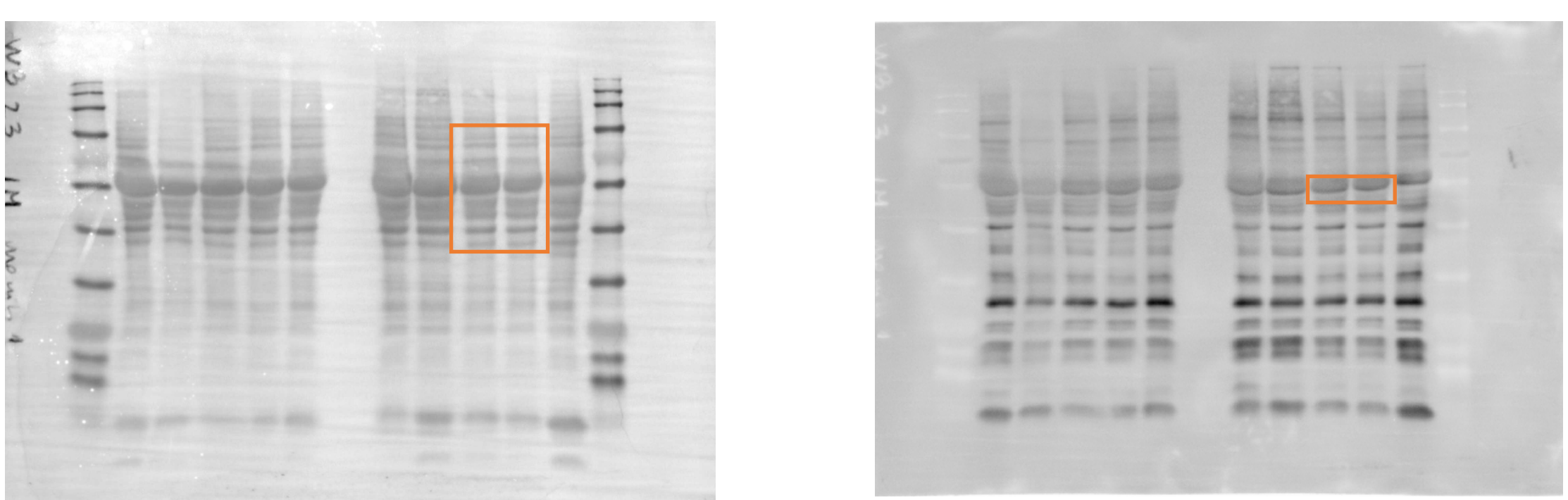

*Figure 5C - Cytoplasmic fraction*

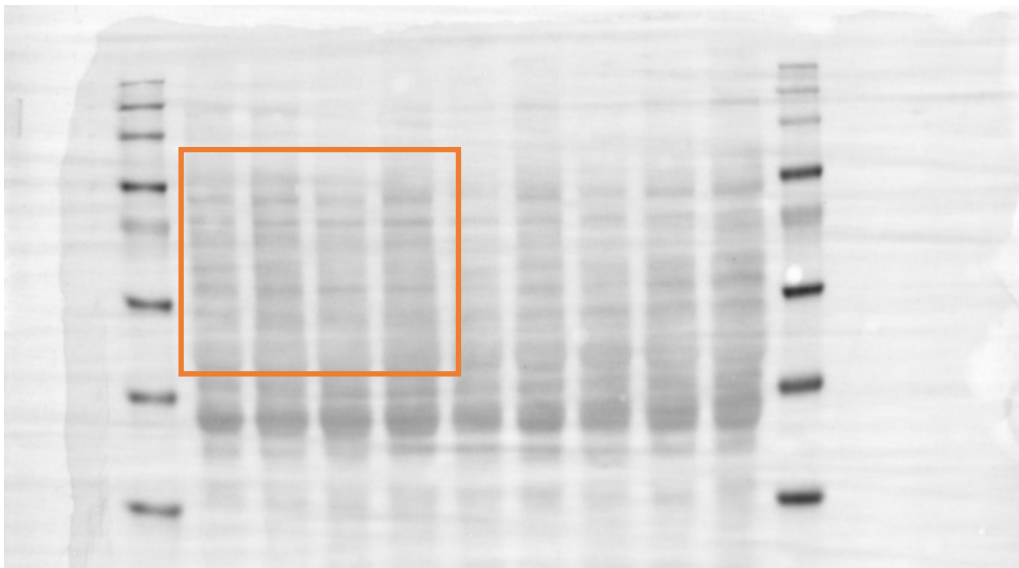

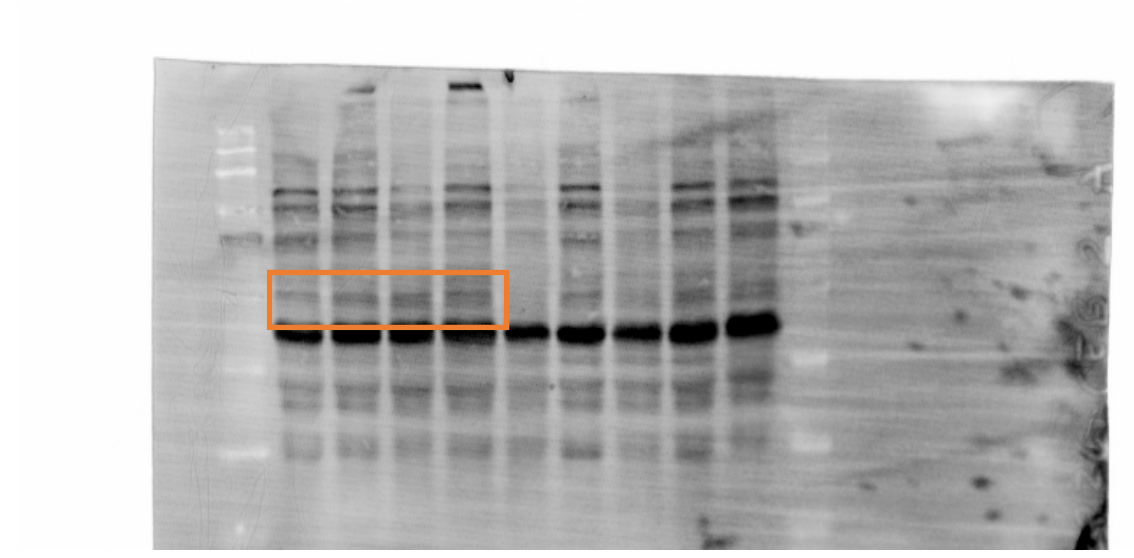

*Figure 5C - Nuclear fraction*

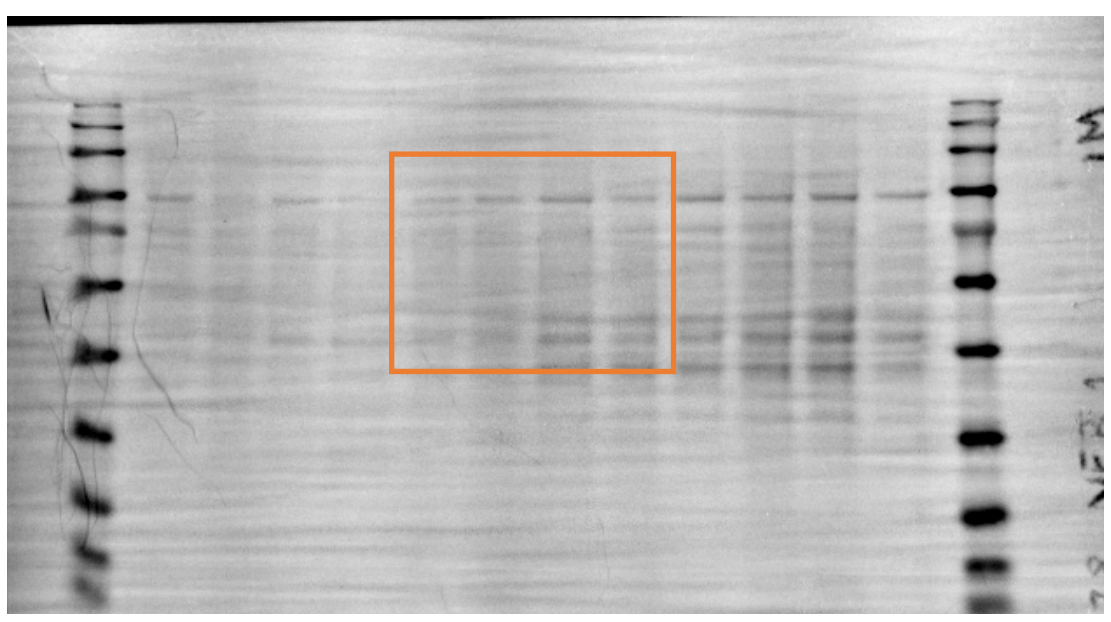

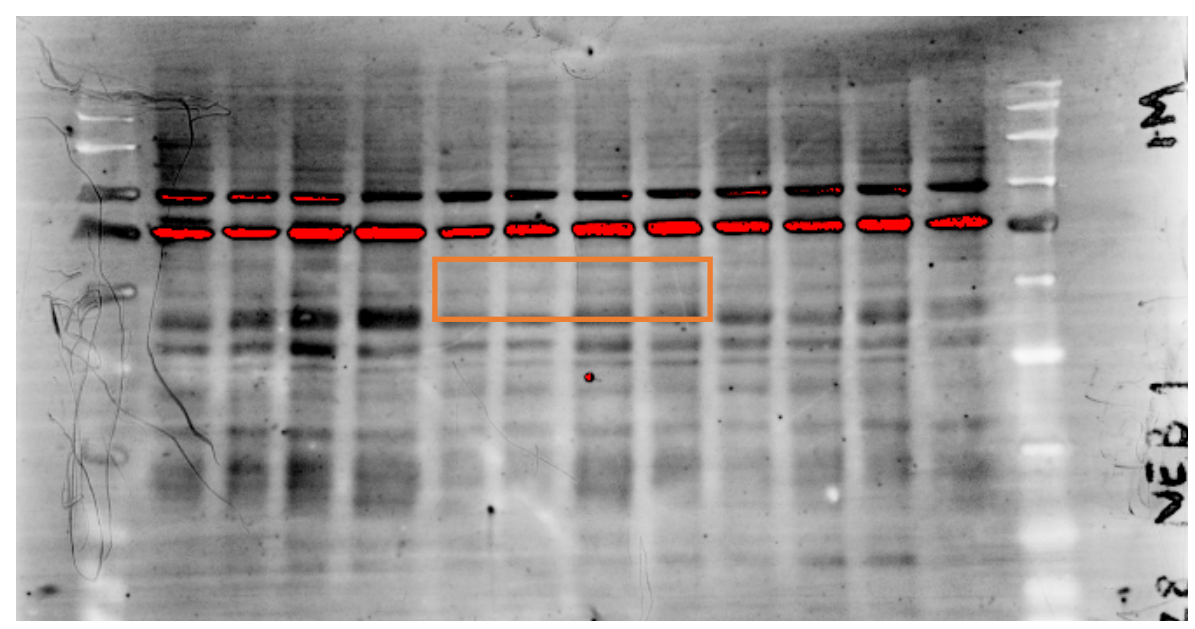

*Figure 6A*

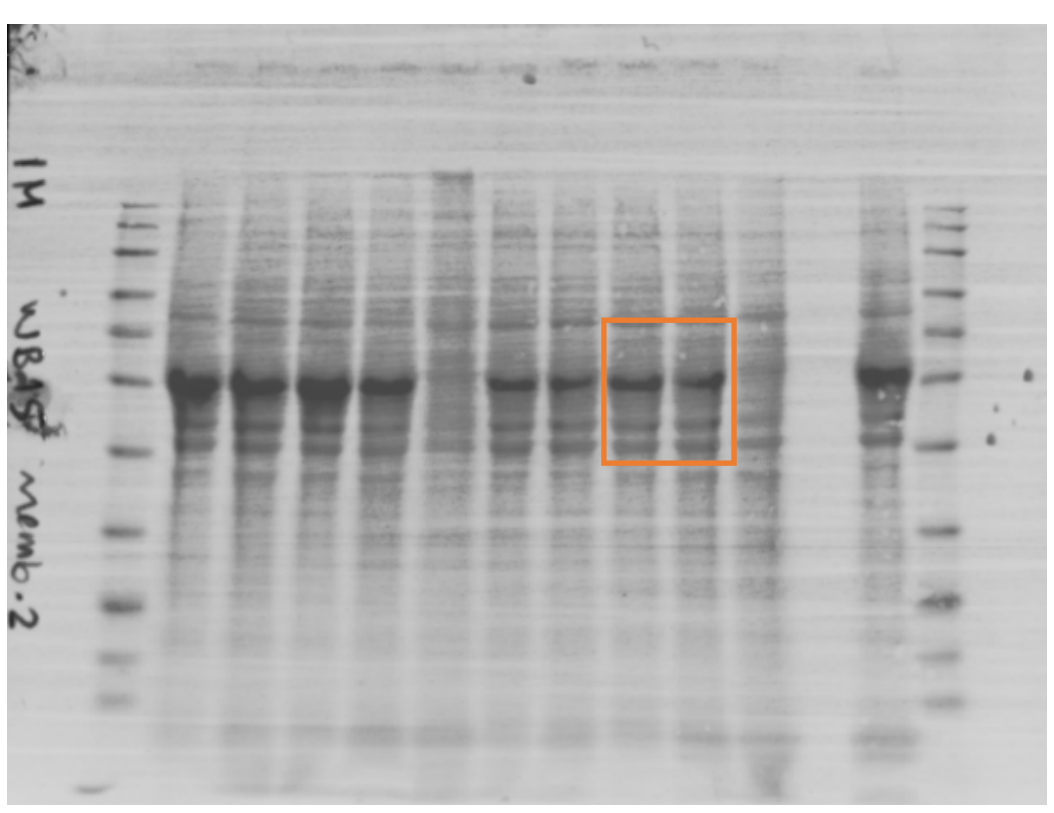

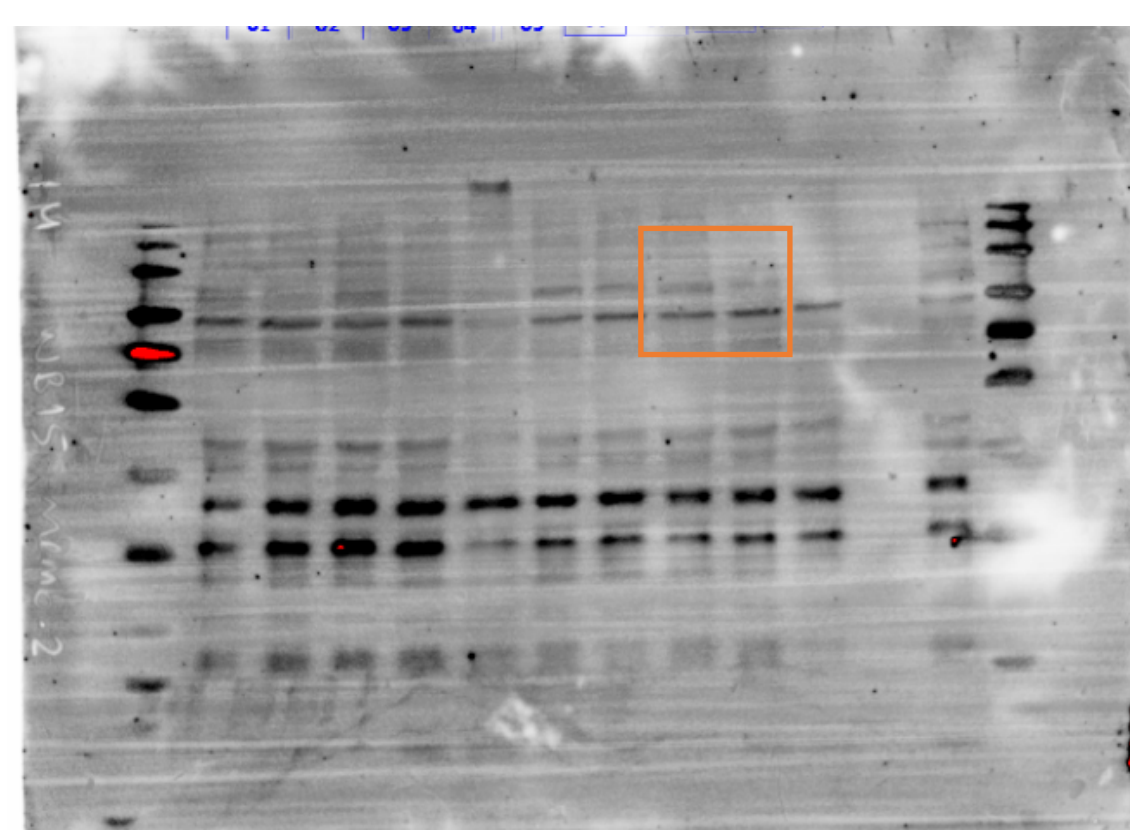

*Figure 6B*

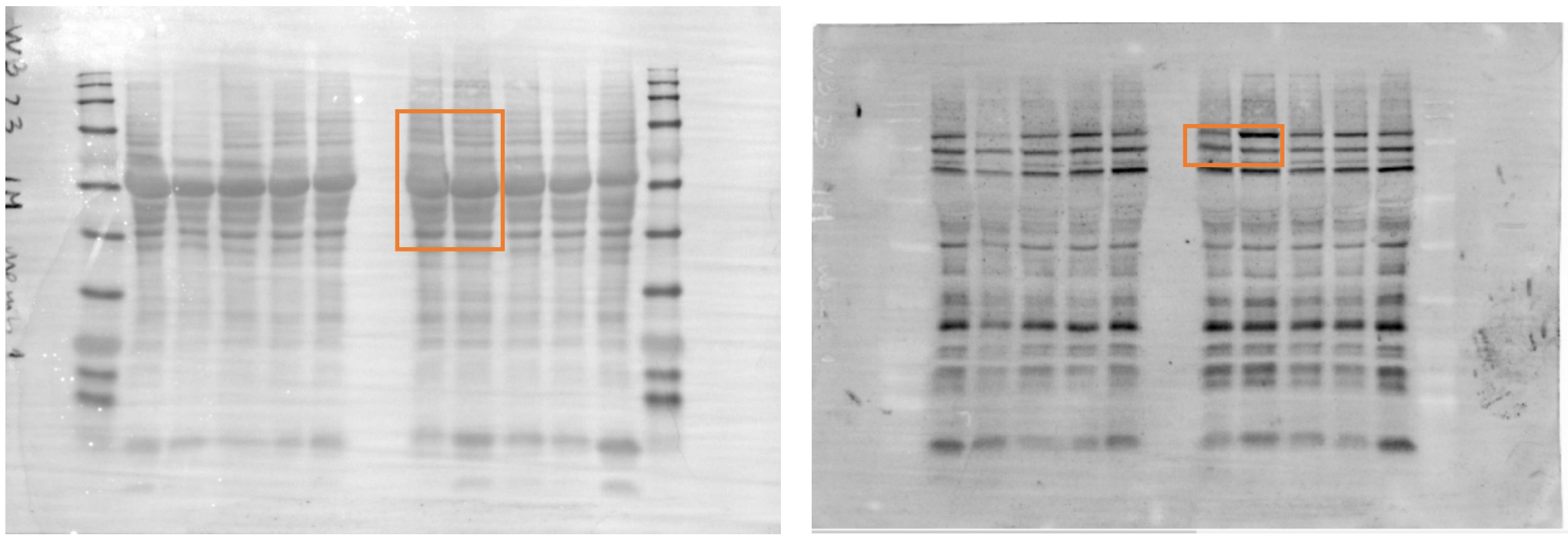

*Figure 6C - Cytoplasmic fraction*

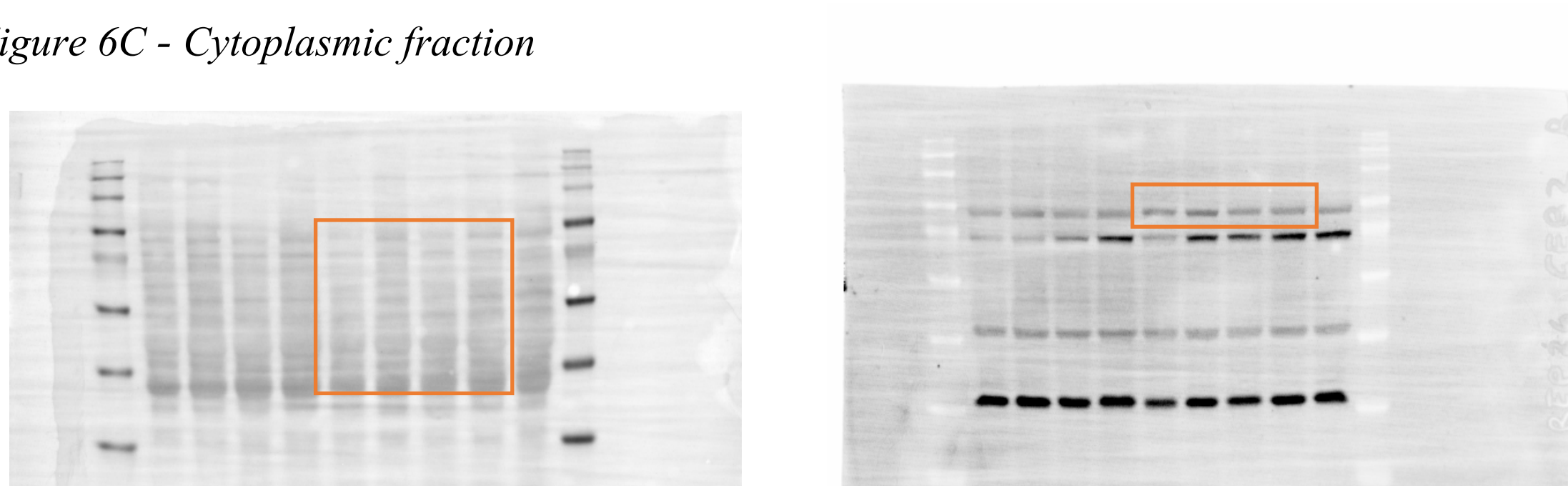

*Figure 6C - Nuclear fraction*

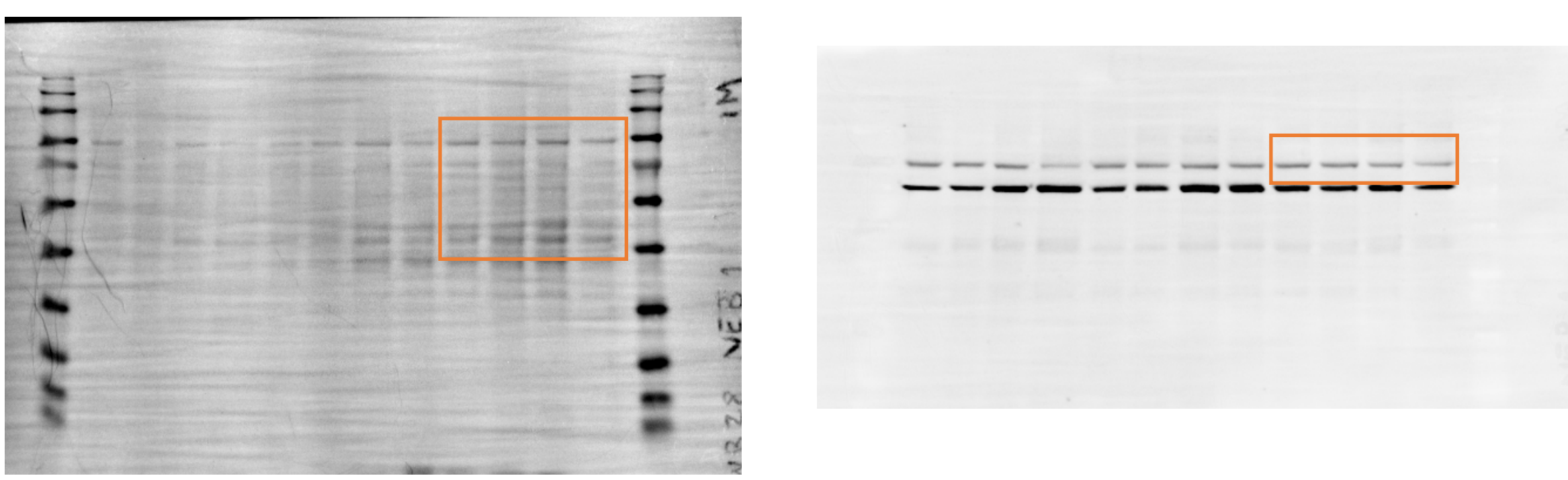

Supplement: Supplementary file 1 [file mmc1.pdf]
